# Human curriculum learning of a cue combination task

**DOI:** 10.1038/s41562-026-02452-1

**Published:** 2026-05-05

**Authors:** Qingtian Mi, Christopher Summerfield

**Affiliations:** Department of Experimental Psychology, University of Oxford, Oxford, UK

**Keywords:** Human behaviour, Human behaviour

## Abstract

Humans often learn better when problems are broken down into parts, but this phenomenon has eluded explanation at the computational level. Here we study how differing training curricula help or hinder learning in a classic probabilistic cue combination task. Training curricula that ‘divide and conquer’ by presenting one cue at a time facilitate later performance on test trials involving multiple cues. This effect is captured by a hybrid learning framework that arbitrates between two different learning strategies: a marginal updating process, which assigns credit to each cue independent of every other, and a joint updating process, which distributes credit across cues on the basis of their joint presence. We use this theory to generate new ‘skewed distribution’ multi-cue curricula that should and should not successfully promote human learning. It makes accurate predictions, demonstrating that we can use computational insights of learning to accelerate human probabilistic learning.

## Abstract (web summary)

When learning complex tasks, people often ‘divide and conquer’. Mi and Summerfield propose a computational theory explaining why breaking problems into simpler components enhances learning and show how this insight can be used to design curricula that accelerate learning.

## Main

Decision-making often requires the integration of information from multiple sources. For example, diagnosing an illness requires a clinician to account for many observable symptoms. This problem has been extensively studied in psychology, neuroscience and statistics and is well understood from both a descriptive and normative standpoint^1–9^. Using paradigms that expose agents to discrete cues or continuous noisy signals before a choice, empirical work has revealed that humans and other animals integrate information approximately optimally, weighting each cue by its reliability^10–13^ and trading off speed and accuracy to roughly maximize expected return^14–19^.

**Fig. 1:**
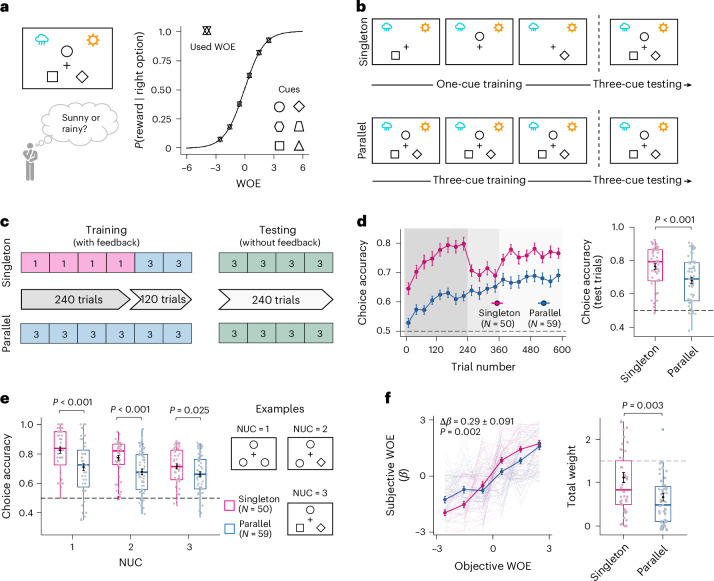
Behavioural task, design and empirical results of Exp. 1. **a**, Participants predicted the weather (sun or rain) on the basis of evidence provided by geometric cues (for example, circle, square and diamond). Each cue was assigned a WOE value that mapped onto the reward probability of a rightward choice via a softmax function (Methods). **b**, In the singleton condition, participants were trained with trials containing a single cue, while in the parallel condition, training involved trials with three cues. Test trials, which included three cues, were the same across conditions. For multi-cue trials, the cues were randomly presented at the three locations around the central fixation in each trial. Participants were randomly assigned to each condition. **c**, Both conditions included a training stage with feedback and a testing stage without feedback. Each square represents a block of 60 trials, with numbers indicating the number of cues per trial and colours denoting trial types (pink for single-cue trials, blue for trials with three unique cues and green for trials with three cues that may not be unique; Methods and Supplementary Fig. 2). **d**, Choice accuracy in Exp. 1. The left panel shows mean choice accuracy (that is, the probability of selecting the better option given the WOEs) plotted over the course of training and test trials. The dots represent the mean choice accuracy across participants, calculated over 30 consecutive trials. The error bars indicate the s.e.m. The dark grey, grey and light grey shaded areas indicate different stages of the task, as in **c**. The right panel shows box plots of mean choice accuracy averaged across test trials. Each dot indicates one participant, the black diamond represents the mean across participants, and the error bars represent the s.e.m. The black dashed line in each panel represents the chance-level choice accuracy (that is, 0.5). **e**, Mean choice accuracy averaged across test trials and grouped by the number of unique cues (NUC). **f**, Subjective WOE. Subjective WOEs for each cue were recovered using logistic regressions (Methods). In the left panel, recovered subjective WOEs are plotted as a function of objective WOEs, with pink and blue dots representing averages across participants in each condition and the error bars representing the s.e.m. Light pink and blue lines show individual participants’ data. In the right panel, box plots display the total weight, calculated from the reliability-weighted subjective WOEs (each cue weighted by the inverse of the squared standard error; Methods). The grey dashed line represents the total weight based on the objective WOEs. In the box plots in **d**–**f**, the central line denotes the median, and the bottom and top edges of the box indicate the 25th and 75th percentiles within a group. The whiskers extend to the minimum and maximum values within 1.5× the interquartile range from the box limits (or to the data extrema if no outliers are present). Points beyond the whiskers are shown as individual outliers. These definitions apply to all subsequent box plots. In **d**–**f**, there are 50 and 59 participants in the singleton and parallel conditions, respectively.

Strikingly, however, we know very little about how humans or other animals learn the appropriate weights associated with each source of evidence. Consider, for example, a well-studied task (based on the weather prediction task) in which geometric shapes (cues) offer independent evidence about whether an outcome will be ‘sun’ or ‘rain’^20–24^. The ground-truth probability of obtaining a reward after choosing one of these outcomes is determined by the summed weight of evidence (WOE) for each of *n* cues:

$${\mathrm{WOE}}_{\mathrm{sum}}=\log \left(\frac{p(\mathrm{sun}|{c}_{1},\,{c}_{2},\ldots {c}_{n})}{p({\mathrm{rain}|c}_{1},\,{c}_{2},\ldots {c}_{n})}\right)=\mathop{\sum }\limits_{i=1}^{n}{\mathrm{WOE}}_{i}$$

where WOE_*i*_ is the true weight assigned by the researcher to each individual cue (Fig. 1a and Methods). Here we asked a simple question: how does the curriculum by which trials are presented to the participant help or hinder the learning of the weight ${\hat{{\rm{WOE}}}}_{i}$ for each cue *i*? In cognitive science we currently lack general, computationally grounded theories of why given curricula may succeed or fail at promoting learning in biological agents. Confusingly, vanilla deep learning systems rarely benefit from temporally structured training regimes, depriving us of a model with which to simulate and understand biological curriculum learning^25–27^.

Nevertheless, in the current paper, we provide such a theory. The starting point for our research was the intuition that many tasks are learned most effectively by being broken down into parts (a ‘divide and conquer’ strategy). Indeed, both humans and monkeys have been reported to acquire the weather prediction task after first being taught about lone cues before progressing to multi-cue combinations^20,23^.

Using a variant in which up to three cues were presented from a total set of six, we compared the performance of two human groups who were respectively exposed to single cues during training (singleton curriculum) or trials involving combinations of three unique cues (parallel curriculum; Fig. 1b and Supplementary Fig. 1; see also Methods). After training, both groups received a brief practice phase on three-cue trials (with feedback). Subsequently, the participants were tested on blocks in which all trials contained three cues; however, these could comprise one, two or three unique cues (for example, a single cue could appear three times in the combination, yielding only one unique cue). On these test blocks, we withheld feedback to provide an outcome variable uncontaminated by further learning (Fig. 1c and Supplementary Figs. 1 and 2).

## Results

### The singleton effect: learning benefits from a divide-and-conquer strategy

The results of Experiment 1 (Exp. 1) are shown in Fig. 1d–f. As expected, participants in the singleton condition learned faster than those in the parallel condition (dark grey shaded area in Fig. 1d (left); interaction in logistic regression: *z* = 4.37; *P* < 0.001; Δ*β* = 0.0036; 95% confidence interval (CI), 0.0020 to 0.0052). However, the performance advantage of singleton training persisted into the test phase, even though participants in the singleton condition were less experienced with trials with three cues (light grey shaded area in Fig. 1d (left); see also Fig. 1d (right); *z* = 3.52; *P* < 0.001; *β* = 0.45; 95% CI, 0.20 to 0.70). Further analysis revealed that, remarkably, participants under singleton training exhibited an advantage on trials with one, two and three unique cues separately (Fig. 1e; see also Supplementary Fig. 4). This latter result is surprising because it contradicts the common observation of encoding specificity, that performance is best when test conditions match those that occurred during training^28^.

Moreover, participants’ choice accuracy increased and their reaction time (RT) decreased as the amount of decision evidence provided by the cues grew (Supplementary Fig. 3). Critically, the advantage of singleton learning remained significant after we controlled for the cue evidence (Supplementary Fig. 3a; *z* = 3.73; *P* < 0.001; *β* = 1.00; 95% CI, 0.48 to 1.52). By contrast, once cue evidence was accounted for, RT did not differ significantly between conditions (Supplementary Fig. 3b; *t*_105.3_ = 0.29; *P* = 0.772; *β* = −0.02; 95% CI, −0.14 to 0.10), indicating that the singleton advantage in accuracy is unlikely to be driven solely by slower responding.

Finally, under singleton training participants learned larger subjective WOE values than those under parallel training (Fig. 1f; left panel, interaction in linear regression: *t*_97.6_ = 3.15; *P* = 0.002; Δ*β* = 0.29; 95% CI, 0.11 to 0.47; right panel, two-sample independent *t*-test: *t*_97.2_ = 3.01; *P* = 0.003; Cohen’s *d* = 0.58; 95% CI, 0.19 to 0.96). The subjective WOEs were recovered via logistic regressions (Methods and Supplementary Fig. 18) and represent a best estimate of the weight that participants assigned to a given cue conditioned on their behaviour. Together, these results suggest that the weather prediction task is learned more effectively by dividing and conquering—training the cues one at a time.

### Robustness of the singleton effect and exclusion of alternative explanations

**Fig. 2:**
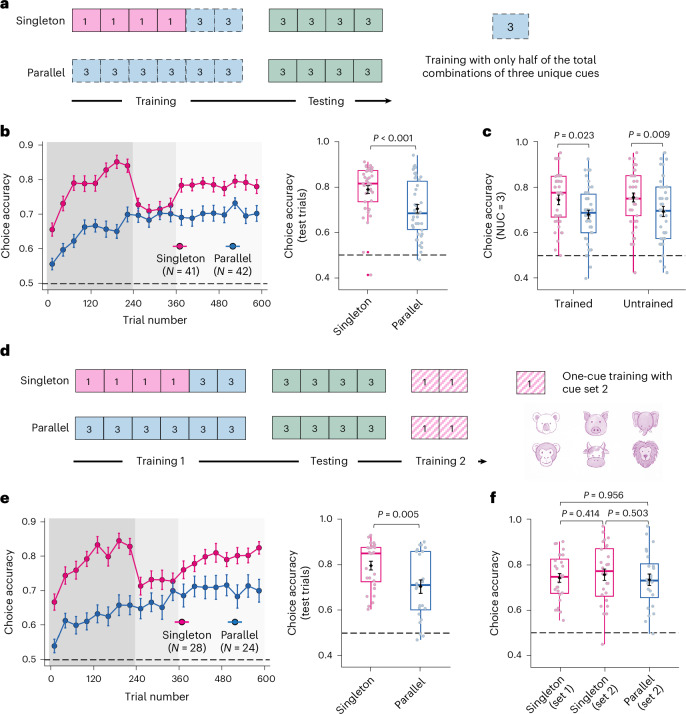
Design and results of Exp. 2 and 3. **a**, Design of Exp. 2. In both conditions, training trials with three cues included only half of the possible combinations of three unique cues (Methods). **b**, Choice accuracy in Exp. 2. Left, mean choice accuracy averaged across participants plotted against trials. Right, mean choice accuracy averaged across all test trials. The error bars represent the s.e.m. **c**, Singleton effect for trained and untrained cue combinations. The box plots illustrate the mean choice accuracy in test trials with three unique cues, grouped by whether the specific combination was included in training trials. In **b** and **c**, there are 41 participants in the singleton condition and 42 in the parallel condition. **d**, Design of Exp. 3. In both conditions, two additional blocks of singleton training with a new set of cues (set 2) were added to the end of the original experiment. The cover story and appearance for set 2 differed from those for set 1 (Methods). **e**, Choice accuracy for geometric cues (set 1). Left, mean choice accuracy averaged across participants plotted against trials. Right, mean choice accuracy averaged across all test trials. The error bars represent the s.e.m. **f**, Choice accuracy for set 2. The box plots display the mean choice accuracy for the extended singleton training with set 2 in both conditions, as well as singleton training with set 1 in the singleton condition. For set 1, only the first 120 trials are used as a baseline for comparison with set 2. In **e** and **f**, there are 28 participants in the singleton condition and 24 in the parallel condition.

We then sought to replicate the singleton effect and rule out potential alternative explanations. First, we confirmed this effect in two independent experiments using similar paradigms (Fig. 2a,b for Exp. 2: *z* = 3.28; *P* < 0.001; *β* = 0.45; 95% CI, 0.22 to ∞; one-tailed; Fig. 2d,e for Exp. 3: *z* = 2.79; *P* = 0.003; *β* = 0.55; 95% CI, 0.22 to ∞; one-tailed; Methods). These replications provide strong evidence that the learning advantage by divide and conquer is robust and replicable.

Second, we tested whether the singleton effect depended on the specific cue combinations during training. In Exp. 2, only half of the possible three-cue combinations were used for training, allowing us to compare test accuracy on trained versus untrained combinations (Fig. 2a and Methods). Strikingly, singleton training improved accuracy not only for trained combinations (Fig. 2c; *z* = 1.99; *P* = 0.023; *β* = 0.32; 95% CI, 0.060 to ∞; one-tailed) but also for novel, untrained ones (*z* = 2.39; *P* = 0.009; *β* = 0.34; 95% CI, 0.103 to ∞; one-tailed). Within each condition, accuracy did not differ between trained and untrained combinations (singleton: *z* = 0.72; *P* = 0.471; *β* = 0.06; 95% CI, −0.10 to 0.22; parallel: *z* = 0.87; *P* = 0.383; *β* = 0.07; 95% CI, −0.08 to 0.22). These findings suggest that the singleton effect generalizes beyond specific training experiences.

Third, we tested whether motivational differences could explain the singleton effect. One possibility is that participants in the singleton condition were more motivated to perform the task. In Exp. 3 we thus repeated Exp. 1 but included a trailing task, after the entire study, in which both groups relearned the weights associated with a new set of cues under singleton training (Fig. 2d and Methods). The results replicated the singleton effect (Fig. 2e), but performance in the trailing task did not differ between groups (Fig. 2f; cue set 2: *z* = 0.67; *P* = 0.503; *β* = 0.14; 95% CI, −0.28 to 0.56). This indicates that both groups were equally motivated to learn a new task. Given that motivation in sequential tasks tends to be partly correlated, it is possible that participants had similar motivations during the main experiment unless their motivations were fully reset in the new task.

More broadly, several other results also argue against motivational differences as the source of the singleton effect. First, RTs during testing did not statistically differ between conditions (Exps. 1–3: *t*_241.4_ = 0.52; *P* = 0.603; *β* = −0.02; 95% CI, −0.10 to 0.06). If participants in the parallel condition had been less motivated, they would probably have responded faster to complete the task more quickly. Second, performance differences between groups remained stable across the testing phase (light grey area in Supplementary Fig. 4a; *z* = 0.70; *P* = 0.486; Δ*β* = 0.0002; 95% CI, −0.00037 to 0.00077), inconsistent with declining motivation. Finally, computational modelling revealed no reliable differences between the two conditions, either in learning-rate decay when learning rates were allowed to decay during training (Supplementary Fig. 14b; *t*_221.2_ = −1.7; *P* = 0.090; *d* = −0.22; 95% CI, −0.47 to 0.03; Methods) or in the learning rates themselves for multi-cue trials when single-cue and multi-cue trials were allowed to have different learning rates in the singleton condition (*α*_+_: *t*_204.3_ = 1.24; *P* = 0.216; *d* = 0.16; 95% CI, −0.09 to 0.41; *α*_−_: *t*_241.5_ = −0.32; *P* = 0.742; *d* = −0.04; 95% CI, −0.29 to 0.21; Methods).

Finally, we tested whether the singleton effect generalizes beyond the current paradigm by introducing a new compositional task, ‘Pointer’. Participants predicted an avatar’s final location on a linear track composed of 21 discrete locations (coordinates −10 to 10) given a random starting location and a sequence of symbols (Methods and Supplementary Fig. 5a). Each symbol denoted one arithmetic operation that moved the avatar along the track (for example, the symbol denoting ‘+1’ moves the avatar from *n* to *n* + 1; Supplementary Fig. 5b). Operations were applied sequentially—the output of one symbol served as the input for the next. Once again, participants were randomly assigned to a singleton condition (one symbol per training trial) or a parallel condition (three symbols per training trial; Supplementary Fig. 5c,d). As expected, the singleton group outperformed the parallel group on identical three-symbol test trials (*z* = 4.67; *P* < 0.001; *β* = 1.77; 95% CI, 1.03 to 2.51; Supplementary Fig. 5e). These results show that the singleton advantage generalizes to a distinct learning context.

### Computational modelling: a hybrid learning mechanism

**Fig. 3:**
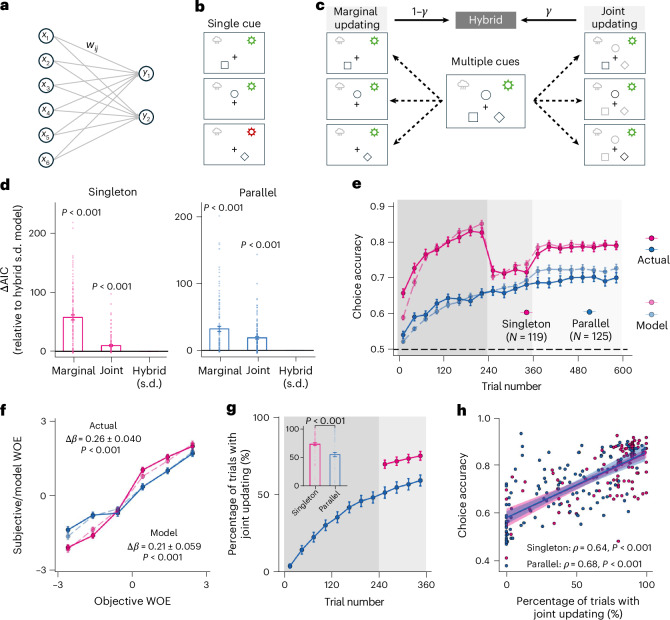
Computational modelling. **a**, Following previous studies^31,32,70^, participants are modelled as a perceptron learner who generates an output *y*_*j*_ on the basis of the summed evidence of presented cues *x*_*i*_ (Methods). The weight *w*_*ij*_, associated with cue *x*_*i*_ and output *y*_*j*_, is updated incrementally by the error between the observed outcome and the network prediction. **b**, Under singleton training, where only one cue is presented per trial, the outcome can be assigned unambiguously to that cue. **c**, Learning strategies under multi-cue training. In marginal updating, each cue is associated directly with the outcome without considering the other presented cues. In joint updating, weights are updated on the basis of the output predicted by the integrated evidence of all cues instead of a single cue. In the hybrid model, the marginal and joint updating strategies are combined, weighted by a free parameter *γ*. **d**, Model comparison. The bar plots display the ΔAIC value (mean ± s.e.m.) of each model, computed relative to the hybrid s.d. model (Methods). Lower values indicate better model fit. Notably, the hybrid model consistently yields lower AIC values than single-strategy models across Exps. 1–3. Each dot represents an individual participant. **e**, Recovered versus observed choice accuracy across trials. The dark solid lines show pooled accuracy from Exps. 1–3. The light dashed lines show predictive performance from the hybrid s.d. model, and similar patterns were observed when the model predictions were simulated from scratch without using participants’ actual choices^76^. **f**, Cue weights (WOE) recovered from participants’ choices in test trials (solid lines; subjective WOE) are compared with weights estimated from the hybrid s.d. model (dashed lines; model WOE). **g**, Proportion of multi-cue trials using joint updating, as estimated by the hybrid s.d. model, plotted over the training course. Marginal and joint updating yield identical results in single-cue trials and are therefore indistinguishable in these trials. The inset shows the mean across all multi-cue training trials. **h**, Relationship between learning performance and the proportion of joint updating trials. Each dot represents an individual participant, and the shaded areas around the fitted line reflect 95% CIs. In **d**–**g**, the error bars represent the s.e.m. In **d**–**h**, there are 119 participants in the singleton condition and 125 in the parallel condition.

Exps. 1–3 provide compelling evidence that divide and conquer is an effective strategy for learning the cue combination task. But why is this the case? Consider a ‘perceptron’ learner who updates the weights associated with each cue using gradient descent in a shallow neural network (Fig. 3a and Methods). This learner generates an output *y* on the basis of a logistic weighted sum of inputs *x*, updating the weights $\hat{w}$ on the basis of a supervision signal *r*. Under the standard perceptron learning rule (assuming a learning rate *α*), the weight associated with each presented cue is updated by ${\hat{w}}_{{ij}}={\hat{w}}_{{ij}}+\alpha (r-{y}_{j}){x}_{i}$, where (*r* − *y*_*j*_) represents the prediction error (PE) signal that drives learning.

The key difference between singleton and parallel training lies in how the observed outcomes are attributed to cues. In singleton training, where only one cue is present, credit assignment is straightforward (Fig. 3b). By contrast, in parallel training with multiple cues, the outcome reflects the integrated evidence of all cues, making credit assignment more challenging. One potential strategy is marginal updating, in which participants update the weight of each cue as if it alone determined the outcome^24,29–32^ (Fig. 3c (left) and Supplementary Fig. 6b (left)). For instance, if a sunny outcome follows the presentation of a square, a circle and a diamond, a marginal updating learner would adjust the weight of the diamond while disregarding the contributions of the other two cues. Alternatively, participants may adopt a joint updating strategy, assigning credit on the basis of the integrated evidence of all cues (Fig. 3c (right) and Supplementary Fig. 6b (right)).

The two learning strategies reflect a trade-off between learning precision and cognitive demand, thereby requiring participants to arbitrate between them under limited cognitive resources^33–35^. Specifically, marginal updating reduces cognitive cost by avoiding evidence integration across cues, but at the expense of learning precision: the learned WOE values reflect the marginal reward probability of each cue and may diverge substantially from the true value under multi-cue training (Supplementary Fig. 6c,d). In contrast, joint updating is assumed to be more cognitively demanding but ensures that cue weights converge on their true values across contexts (Supplementary Fig. 6c).

Inspired by previous studies^36–40^, we developed a hybrid learning framework in which learning reflects the combined influence of joint and marginal updating. Specifically, this framework assumes that participants do not rely exclusively on joint updating but often use simpler marginal updating when credit assignment is challenging in multi-cue settings. The relative contribution of each strategy is governed by a weighting parameter *γ*. A higher *γ* reflects greater reliance on joint updating (Fig. 3c and Supplementary Fig. 6a; see also Methods). We considered two forms of hybrid learning (Supplementary Fig. 6e): (1) a hybrid constant model where *γ* remains fixed across trials and (2) hybrid adaptive models where *γ* varies dynamically to balance the gain in learning precision against the relative cognitive cost of joint (versus marginal) updating (Methods), consistent with a resource-rational account^33–35^.

For clarity and interpretability, we used a threshold-based rule to implement the arbitration between different learning strategies in the hybrid adaptive models. Specifically, *γ* is set to either 0 (that is, marginal updating) or 1 (that is, joint updating) in each training trial, meaning the learning strategy switches between marginal and joint updating. This discretization is not central to the theory: a continuous mixture in which *γ* varies smoothly between 0 and 1 yields comparable fits and the same conclusions. We therefore retained the threshold-based formulation as a parsimonious and interpretable implementation of hybrid learning. The relative cognitive cost is estimated as a free parameter for each participant, while the gain in the learning precision for the joint (versus marginal) updating is quantified in two ways.

The first approach quantifies the gain as the difference between the PEs generated by marginal and joint updating (hybrid PE model; Supplementary Fig. 6e (middle)). Larger discrepancies indicate greater gain in learning precision under joint updating, thereby making joint updating more advantageous. Although theoretically rigorous, this approach is somewhat unrealistic as it requires computing the joint updating PE before choosing a strategy. The second approach offers a heuristic approximation: it calculates the gain as the standard deviation of the learned WOE values across the presented cues (hybrid s.d. model; Supplementary Fig. 6e (right)). As marginal updating attributes outcomes uniformly across cues, it introduces greater distortion in weight updating when WOEs vary widely (that is, a higher s.d.), overweighting weak cues and underweighting strong ones. Consistent with this idea, higher standard deviations strongly correlated with larger PE distances (Spearman’s *ρ* = 0.96, *P* ≈ 0; Supplementary Fig. 6g). Moreover, s.d. was behaviourally relevant: smaller standard deviations were associated with higher accuracy and faster responses (accuracy: *z* = −6.72; *P* < 0.001; *β* = −0.23; 95% CI, −0.30 to −0.16; RT: *t*_151.5_ = 15.92; *P* < 0.001; *β* = 0.21; 95% CI, 0.18 to 0.24).

The intuition behind the hybrid s.d. model is that the reliability of marginal versus joint updating depends on the disparity in the evidence strength of cues. When cue weights vary widely (that is, large s.d.), marginal updating can substantially mislead learning, whereas joint updating avoids such errors. For instance, in Fig. 3c, if a square and a circle support ‘sunny’ but a diamond strongly supports ‘rain’, a sunny outcome would cause marginal updating to generate a large, misleading PE for diamond, incorrectly weakening the diamond–rain association. In contrast, by integrating evidence across all cues, joint updating corrects this imbalance and yields more accurate learning. Conversely, when cue weights are similar (for example, s.d. ≈ 0), both strategies converge to nearly identical updates, making marginal updating preferable due to its lower cognitive cost.

### Hybrid learning model outperforms single-strategy models

We fitted participants’ trial-by-trial choices with each of the five models: marginal only, joint only, hybrid constant, hybrid PE and hybrid s.d. (Supplementary Table 1). Across conditions, both hybrid PE and hybrid s.d. models achieved lower Akaike information criterion (AIC) values than the hybrid constant and single-strategy models (Fig. 3d and Supplementary Fig. 6f), implying that participants dynamically adjusted their learning strategies. While the hybrid PE model fitted better in the parallel condition (Supplementary Fig. 6f), the hybrid s.d. model was more cognitively plausible and could capture behavioural patterns quite well (Fig. 3e,f), so we adopted the hybrid s.d. model for subsequent analyses unless otherwise noted. In fact, these two hybrid adaptive models yielded similar results, as they were highly correlated. Notably, the hybrid s.d. model continued to outperform single-strategy alternatives even when model complexity was more strongly penalized using the Bayesian information criterion.

The hybrid s.d. model includes four free parameters (Supplementary Table 1): two learning rates for positive and negative outcomes (*α*_+_ and *α*_−_; see Supplementary Fig. 7a,b for models with the same learning rate), one parameter capturing the evidence integration cost (*β*_1_; see Supplementary Fig. 7c,d for models without an integration cost) and one parameter reflecting the relative cognitive cost of joint versus marginal updating (*θ*). Model and parameter recovery confirmed that the hybrid s.d. model was distinguishable from alternatives and its parameters could be reliably estimated (Supplementary Fig. 8). Parameter comparisons revealed no group differences in *α*_−_, *β*_1_ or *θ*, but *α*_+_ was significantly larger in the singleton condition due to reduced credit-assignment demands in single-cue trials (Supplementary Fig. 9a–d; see Supplementary Fig. 9e–h for parameter-performance correlations). However, parameter differences across conditions alone could not explain the singleton advantage, which persisted even after we controlled for these differences (Supplementary Figs. 9e and 10).

What, then, drives the learning advantage of singleton training? One possibility is the reliance on joint updating during multi-cue trials. Singleton training allows participants to rely more on joint updating, thereby leading to a better learning performance. To test this, we computed the proportion of multi-cue trials with joint updating, as inferred from the hybrid s.d. model. This proportion increased gradually in both conditions but was consistently lower in the parallel condition (Fig. 3g; see Supplementary Fig. 11 for example participants). This lower prevalence of joint updating in the parallel condition is expected, as joint updating is assumed to be more cognitively demanding and may therefore be adopted primarily when cues are sufficiently distinguishable—a criterion more readily met after singleton training than during early parallel training. Across participants, a higher proportion of joint updating trials was associated with greater accuracy (Fig. 3h). Crucially, when we used condition and proportion of joint updating to predict choice accuracy in a single regression, the condition effect was eliminated (condition: *t* = −0.31; *P* = 0.755; *β* = −0.004; 95% CI, −0.029 to 0.021; joint updating: *t* = 14.82; *P* < 0.001; *β* = 0.27; 95% CI, 0.23 to 0.31). These results suggest that differences in strategy use—specifically, the greater reliance on joint updating in singleton training—explain the singleton effect.

We also tested a series of alternative explanations. First, in the singleton condition, behaviour could not be better explained by participants ceasing to learn during three-cue trials, nor by assuming different learning rates for single- versus three-cue trials (Supplementary Fig. 12). Second, in both conditions, the observed patterns could not be captured by models in which strategy choice depended only on trial number or reward outcome (Supplementary Fig. 13a). Third, learning behaviour depended on the specific assignment of updating strategies to each trial, not just the overall proportion of joint updating (Supplementary Fig. 13b). Finally, although extensions such as learning-rate decay or cue-specific attentional bias improved overall model fit, the hybrid learning model continued to outperform single-strategy models (Supplementary Fig. 14). We retained the hybrid model without extensions as the reference model for the main analyses, as it directly captures the core theoretical mechanism—the arbitration between marginal and joint updating—with a minimal and interpretable parameter set. While these extensions enhance quantitative fit, they do not alter the qualitative conclusions or the necessity of a hybrid learning mechanism.

The hybrid learning framework thus offered a solid foundation for predicting which curricula should enhance, impair or have no effect on human learning. We next tested these predictions in three independent experiments. Importantly, such predictions follow from the framework’s core theoretical claim—that learners rely partially on a simpler marginal updating strategy under cognitive resource constraints—rather than from any specific model implementation. The hybrid s.d. model serves to formalize and quantify this claim and to improve the alignment between predicted and observed learning dynamics.

### Designing new curricula to improve or hinder learning

We first asked whether the hybrid learning framework could be used to identify a condition in which test performance after parallel training would match or exceed that following singleton training. A key prediction of this theoretical framework is that learners initially rely more on marginal updating and gradually increase their use of joint updating over training. This implies that if the training curriculum is structured to make marginal updating more effective, participants should be able to acquire more accurate cue weights.

**Fig. 4:**
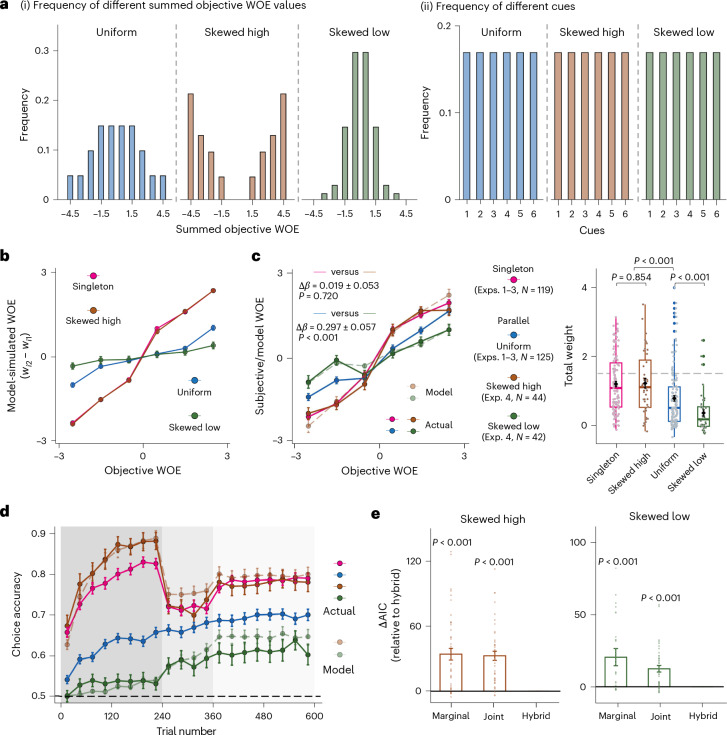
Changing the distribution of training stimuli to improve or hinder learning. **a**, Distribution of summed objective WOE values and cue frequencies for different training curricula. Left, the distribution of the summed objective WOE for training trials across the uniform (original parallel), skewed high and skewed low conditions (i). Right, the number of times each cue appeared in training trials for the three conditions (ii). **b**, Out-of-sample model simulated WOE value for each cue as a function of its objective WOE, based on simulations of the hybrid s.d. model. **c**, Left, subjective WOE values recovered by logistic regressions (solid lines) and model-learned WOE values derived from the hybrid s.d. model (dashed lines), plotted against the objective WOE. Right, total weight calculated from the subjective WOE values (Methods). Each dot represents one individual participant. **d**, Mean choice accuracy averaged across trials and participants, plotted as a function of trial number. The solid and dashed lines represent the actual and model-recovered choice accuracies, respectively. **e**, Bar plots displaying the mean ΔAIC value of each model, averaged across participants and plotted separately for each condition. Each dot represents an individual participant. In **b**–**e**, the error bars represent the s.e.m. In **b**–**e**, there are 119, 125, 44 and 42 participants in the singleton, parallel uniform, skewed high and skewed low conditions, respectively.

To evaluate this prediction, we searched for alternative distributions of cue combinations (and WOEs) in the parallel condition that could alter learning, while holding constant the overall frequency of each individual cue and equating these to the frequencies in the singleton condition (Fig. 4a and Methods). In other words, although the distribution of cue combinations varied across conditions, each cue appeared equally often in all training regimes. We used the hybrid s.d. model, with parameters independently derived from Exps. 1–3, to quantify how learning under these alternative curricula should unfold. These out-of-sample simulations indicated that increasing the prevalence of cue combinations with more extreme WOEs (the parallel skewed high condition) should improve performance, whereas emphasizing less extreme WOEs (the parallel skewed low condition) should impair performance relative to the standard uniform parallel condition (Fig. 4b).

We tested these predictions in Exp. 4. As expected, participants trained under the skewed high curriculum performed better at test (Fig. 4c,d), with accuracy exceeding that of participants trained in the parallel uniform condition (Supplementary Fig. 15a; *z* = 3.74; *P* < 0.001; *β* = 0.448; 95% CI, 0.25 to ∞; one-tailed) and indistinguishable from that under singleton training (*z* = −0.184; *P* = 0.854; *β* = −0.021; 95% CI, −0.24 to 0.20). By contrast, the skewed low curriculum produced the poorest performance, reliably below that in all other conditions (skewed low versus uniform: *z* = −3.30; *P* < 0.001; *β* = −0.393; 95% CI, −∞ to −0.20; one-tailed). These results were further confirmed by the recovered subjective WOE values for each cue (Fig. 4c). A direct comparison between the two skewed training conditions showed that participants in the skewed high condition performed better even on cue combinations for which they had received less training (Supplementary Fig. 15b; *z* = 3.83; *P* < 0.001; *β* = 0.448; 95% CI, 0.22 to 0.68). Together, these findings confirm the predictions of the proposed learning framework, demonstrating its potential to identify new curricula that accelerate human learning in multi-cue settings.

We next examined whether the hybrid learning model could capture participants’ learning dynamics under the skewed curricula. The results of model fitting again showed that the hybrid s.d. model provided a better account (Fig. 4e). The behavioural patterns recovered from this model also closely tracked the observed data in both the accuracy curves and subjective WOE values (Fig. 4c,d). In both skewed conditions, the proportion of joint updating increased gradually during training, with higher levels in the skewed high condition than in the skewed low condition (Supplementary Fig. 15c). This proportion was also positively correlated with learning performance in both conditions (Supplementary Fig. 15d). Interestingly, however, controlling for the proportion of joint updating did not fully explain the performance difference between skewed high and skewed low training (*t* = 4.80; *P* < 0.001; *β* = 0.10; 95% CI, 0.06 to 0.14). This suggests that the skewed high curriculum may provide an additional learning advantage, probably by making marginal updating itself more effective during training.

### Idealized parallel training cannot improve learning performance

**Fig. 5:**
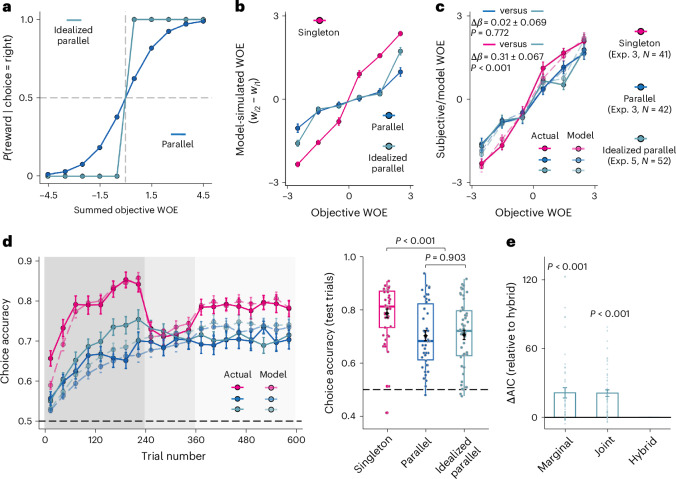
No improvement in learning performance under idealized parallel training. **a**, In idealized parallel training (blue-grey line), the feedback is determined by the sign of the summed objective WOE: a rightward choice is rewarded when the summed objective WOE value is larger than 0; otherwise, a leftward choice is rewarded. **b**, Out-of-sample model-simulated WOE values for each cue based on the hybrid s.d. model. **c**, Subjective WOE values recovered by logistic regressions (solid lines) and model-learned WOE values derived from the hybrid s.d. model (dashed lines), plotted against the objective WOE. **d**, Left, mean choice accuracy averaged across trials and participants, plotted as a function of trials. The solid and dashed lines represent the actual and model-recovered choice accuracies, respectively. Right, mean choice accuracy averaged across test trials for each condition separately. **e**, Bar plots displaying the mean ΔAIC value of each model, averaged across participants in the idealized parallel condition. Each dot represents an individual participant. In **b**–**e**, the error bars represent the s.e.m. In **b**–**e**, there are 41, 42 and 52 participants in the singleton, parallel and idealized parallel conditions, respectively.

Previous studies have shown that ‘idealized’ training, in which feedback is provided deterministically, can facilitate learning about noisy or probabilistic signals^41,42^. In our task, deterministic feedback corresponded to the sign of the summed objective WOE (the idealized parallel condition; Fig. 5a and Methods). However, our theoretical framework together with the simulations using the hybrid s.d. model showed that such idealized feedback would not enhance performance in the cue combination setting (Fig. 5b).

We tested this prediction in Exp. 5 with a new cohort. As anticipated, idealized parallel training did not improve test performance relative to the standard parallel condition (*z* = 0.12; *P* = 0.903; *β* = 0.017; 95% CI, −0.25 to 0.29) and remained reliably below singleton training (*z* = −3.26; *P* < 0.001; *β* = −0.434; 95% CI, −∞ to −0.22; one-tailed; Fig. 5c,d). Nonetheless, participants did show higher accuracy on trained combinations than on untrained ones (Supplementary Fig. 16), perhaps due to better memorization of the trained stimuli and their associated rewarded choices once stochasticity had been removed. As in other conditions, participants’ behaviour under idealized training was better captured by the hybrid s.d. model (Fig. 5e), whose recovered behavioural patterns closely mirrored the empirical observations.

### No learning differences between ascending and descending curricula

Finally, another prominent motif in curriculum design is the notion that training should ‘start small’, gradually progressing from simple to complex examples. Whether this applies to our cue combination learning, however, remains unclear. To address this, we introduced two new conditions (Supplementary Fig. 17a): one in which the number of cues gradually increased across training (from 1 to 2 to 3, the ascending condition) and one where it decreased (from 3 to 2 to 1, the descending condition). Although intuition might suggest an advantage for ascending training, the model simulations under the hybrid learning framework indicated no performance differences between the two (Supplementary Fig. 17b). Moreover, it anticipated that both curricula would yield learning comparable to singleton training and superior to parallel training (Supplementary Fig. 17b).

In Exp. 6, participants were trained under ascending, descending, singleton and parallel curricula. Consistent with the theoretical predictions, ascending, descending and singleton curricula produced comparable learning performance, each outperforming the parallel condition (Supplementary Fig. 17c,e). Model fitting further confirmed that across all four curricula, participants’ choices were better explained by the hybrid s.d. model than by single-strategy alternatives (Supplementary Fig. 17g). The behavioural patterns recovered from the model also closely matched the observed trajectories (Supplementary Fig. 17c–f).

## Discussion

How and why humans learn from differing curricula has been a prominent question in education research, with a particular focus on how the spacing of training items can enhance memorization^43^. But we lack general, computationally grounded theories of how and why curricula may work in basic task learning. Here we illustrate one simple case where humans benefit from a divide-and-conquer strategy—from breaking down a problem into its constituent parts and training each part individually. We describe a computational framework that can account for this effect with a hybrid learning mechanism arbitrating dynamically between two different learning strategies.

We use this computational framework to successfully predict the efficacy of various additional curriculum interventions. Specifically, it predicts that in our cue combination task, starting small (training lone cues before multi-cue arrays) and idealized training (switching from stochastic to deterministic feedback) are ineffective, while skewing cue combinations towards extremes is beneficial. These predictions were empirically confirmed. Together, our results demonstrate how computational insights on learning can guide the design of curricula that accelerate learning. However, our paradigm involves a simplified setting with a small, known set of cues, and it remains open whether these principles generalize to real-world learning involving unknown latent causes.

The use of a hybrid learning mechanism has been widely observed in contexts from non-social reinforcement learning to strategic decision-making^36–40^. In the classic two-stage learning task^37^, for example, learners arbitrate between model-free and model-based strategies. Importantly, prior work has shown that the weighting of different learning and control strategies adjusts dynamically with context, experience and the reliability of signals^38,39,44–47^ and has identified neural mechanisms supporting this arbitration process^38,39,47^. Our study builds on this literature by examining how training curricula shape such strategy arbitration during learning in a cue-combination task.

Specifically, we show how different curricula bias learners towards different regions of the strategy space by altering the relative benefits and costs of alternative learning strategies. From a resource-rational perspective^33–35^, learners should weigh the benefits of a learning strategy against its cognitive cost, selecting the one that maximizes expected utility under the resource constraints. Consistent with this view, we found that learners rely more on joint updating when its precision advantage outweighs its additional effort relative to marginal updating, and that curriculum structure systematically influences this trade-off. This framework thus helps explain why certain curricula are more effective than others and provides a principled basis for designing training regimes that improve learning outcomes.

We also considered other factors that may shape learning and interact with the proposed hybrid learning mechanism. Prior studies have shown that people allocate attention unevenly across features, assigning greater weight to cues that are more predictive of reward^24,48,49^. In line with these studies, incorporating attentional weights for each cue improved the model fit. Importantly, this extension does not conflict with the hybrid framework but can be naturally integrated into it. More broadly, a hybrid learning model that incorporates such elements—attentional bias or learning-rate decay—not only outperforms a simpler hybrid model but also surpasses single-strategy models that include these features. This underscores the value of hybrid learning as a general and flexible framework for understanding how people learn adaptively in multi-dimensional environments. However, our account in the current study is formulated at the computational level. Future work should investigate how curriculum effects are implemented at the neural level and how these mechanisms interact with broader cognitive systems involved in attention, memory and control^48,50^.

Whether the divide-and-conquer strategy established in our task can generalize to other forms of learning warrants further investigation. This approach has been shown to be an effective strategy across a wide range of domains^51–56^. A key advantage of this strategy lies in its compositional nature: by mastering individual components, learners can flexibly recombine them to address novel challenges or adapt to new contexts^56–59^. As seen here, single-cue training supports decisions about unseen multi-cue combinations. However, when subcomponents interact in non-additive or context-dependent ways, the benefit of a divide-and-conquer approach may be reduced^58,60,61^. In such cases, successful learning may require additional mechanisms—such as joint representation, integration through higher-order rules or explicit feedback—that allow learners to capture dependencies between components. Identifying when learners switch between decompositional and integrative strategies is an important direction for future research.

Curriculum design is central to efficient learning in fields ranging from human education to artificial intelligence^53^. Our study provides a computational account of human curriculum learning and demonstrates that it can be used to design new training regimes that improve learning. Looking forward, there is a unique opportunity to use machine learning methods to design new, data-driven training curricula that could accelerate the learning of both humans and artificial intelligence beyond what theory alone would predict^62–64^. Such a direction highlights the potential of integrating human and artificial learning research to advance both cognitive science and artificial intelligence.

## Methods

### Experiment 1

#### Participants

Participants were recruited via the online platform Prolific (https://www.prolific.com). The eligibility criteria included the following: age between 18 and 30 years, a Prolific approval rate above 90%, no colour blindness, no history of neurological or psychiatric illness and no prior participation in the previous versions of this study. Eligible participants were randomly assigned to different experimental conditions.

A total of 109 participants (*N* = 50 in the singleton condition and *N* = 59 in the parallel condition; 58 females, 50 males and 1 other; age, 25.2 ± 3.37 yr (mean ± s.d.)) were included in the data analyses in Exp. 1. Seventeen participants (13.5% of the total) were excluded from data analyses for meeting one or more of the following criteria: (1) providing the same response for more than 30 consecutive trials (half the total trials in a block), (2) having an average button press count exceeding 1.5 per trial in a block, (3) having RTs below 250 ms for more than 12 trials in a block (20% of the total trials in a block), (4) having low accuracy in sanity check trials or (5) reporting issues with stimulus display. Across all experiments, the exclusion rate ranged from 8.8% to 22.5%, and the mean exclusion rate of all experiments was 16.6%, comparable to other online behavioural studies^65–67^. None of the experiments were preregistered. No statistical methods were used to predetermine sample sizes, but our sample sizes are similar to those reported in previous studies^68,69^.

Ethical approval was obtained from the University of Oxford Central University Research Ethics Committee. All participants provided informed consent through an online form. We followed the same procedure and criteria as described above to recruit and exclude participants in all experiments in this study.

#### Weather prediction task

The participants completed a weather prediction task where they were asked to predict a binary outcome—either sunny or rainy—on the basis of visual cues represented by geometric shapes. On each trial, up to three cues were presented randomly in the three possible locations equidistant from the central fixation (see also Supplementary Fig. 1). Icons representing sunny and rainy options (that is, a sun and a rain cloud, respectively) were displayed on the top left and top right of the screen, with their positions fixed for each participant and counterbalanced across participants. On the basis of the presented cues, participants made their prediction by pressing either the left or right arrow key, corresponding to the respective weather icon. Correct predictions would lead to monetary rewards at the end of the experiment.

Each cue was associated with a WOE, which determined the probability of receiving a reward for selecting the ‘rightward’ choice using a softmax function:

$$P({\rm{reward}}|{\rm{right}})=\frac{{{{e}}}^{{{\rm{WOE}}}_{i}}}{1+{{{e}}}^{{{\rm{WOE}}}_{i}}}$$

We conducted all analyses in the frame of reference of ‘choose left’ versus ‘choose right’.

All experiments included the same set of six different cues, which were randomly assigned a WOE selected from the set [−2.5, −1.5, −0.5, 0.5, 1.5, 2.5], without replacement. These WOEs corresponded to reward probabilities of [0.08, 0.18, 0.38, 0.62, 0.82, 0.92], if participants made a rightward choice. The mapping between WOEs and cues remained constant throughout the experiment for each participant and was randomized across participants.

When multiple cues were presented in a trial, the overall reward probability for a rightward choice was determined by the summed WOE of all presented cues. Specifically, for a set of cues {WOE_1_, WOE_2_, …, WOE_*n*_}, the summed WOE (WOE_sum_) was calculated as:

$${\mathrm{WOE}}_{{sum}}=\mathop{\sum }\limits_{i=1}^{n}{\mathrm{WOE}}_{i}$$

The resulting reward probability for a rightward choice was then:

$$P({\rm{reward}}|{\rm{right}})=\frac{{{{e}}}^{{{\rm{WOE}}}_{{{sum}}}}}{1+{{{e}}}^{{{\rm{WOE}}}_{{{sum}}}}}$$

#### Experimental procedure

The current study investigated the influence of different training curricula on learning performance in the weather prediction task. Each experiment therefore consisted of two stages: a training stage and a testing stage.

During the training stage, participants received immediate feedback after each prediction to facilitate learning. The reward feedback was probabilistically determined by the WOEs associated with the presented cues, as described above. In the testing stage, no feedback was provided to ensure that participants’ performance reflected their learning from the training stage rather than ongoing feedback. This design allowed us to cleanly evaluate how different learning curricula impacted participants’ ability to perform the weather prediction task.

Before starting the task, the participants read detailed instructions explaining the basic set-up of the experiment. After the instructions, they completed a quiz to ensure comprehension of the task rules. Participants who failed the quiz were required to review the instructions and retake the quiz, with up to three attempts allowed. Those who failed all three attempts were unable to continue with the experiment. Participants who successfully passed the quiz proceeded to the formal experiment, where they were informed that they would earn rewards for correct predictions (that is, if the weather predicted by the participants was the actual weather predicted by the presented cue or cues in a trial). Specifically, they were paid on the basis of their performance in a randomly selected half of all training and test trials (£0.01 per correct prediction) in addition to a task completion fee of approximately £10 per hour.

#### Experimental conditions

The study consisted of multiple experiments. In Exp. 1, there were just two experimental conditions: singleton and parallel, differing only in the number of simultaneous cues presented on each trial during the initial training phase.

In the singleton condition (Fig. 1b,c (top)), participants were first trained with single-cue trials. This phase consisted of four blocks of singleton training (60 trials per block, 240 trials in total). Following this, participants completed two blocks (120 trials) of three-cue training to practise integrating evidence from multiple cues. It is necessary to provide at least some multi-cue training in singleton trials because otherwise participants cannot infer the combination rule for multi-cue trials (here, summation over WOEs). Finally, participants entered the testing phase, completing four blocks (240 trials) of three-cue trials without receiving feedback on their predictions.

In the parallel condition (Fig. 1b,c (bottom)), participants were trained with three cues presented simultaneously in every training trial. They completed six blocks of three-cue training (360 trials in total), bypassing the single-cue learning phase. The testing phase was identical to that in the singleton condition, with participants completing four blocks (240 trials) of three-cue trials without feedback.

#### Trial time course

A schematic representation of the weather prediction task and the timeline of each trial is shown in Supplementary Fig. 1.

Each trial began with a cross fixation displayed at the centre of the screen for 1 s. Following this, the cue or cues were presented around the central fixation, and icons representing sunny and rainy weather appeared at the top of the screen. The participants had up to 10 s to predict the weather on the basis of the presented cues by pressing either the left or the right arrow key, corresponding to the left or the right weather icon, respectively. If no response was made within the time limit, a timeout message was displayed, and participants were forced to enter the next trial. If participants failed to make a response for more than 15 trials, they were not allowed to continue the experiment.

Once participants made their choice, a black arrow appeared below the selected option for 0.5 s. In training blocks, participants received feedback about their prediction. If the predicted weather was correct, the chosen weather icon turned green for 1 s before the next trial began. If the predicted weather was incorrect, the chosen weather icon turned red for 1 s, followed by the unchosen weather icon turning green for 1 s to indicate the correct weather predicted by the cues.

In testing blocks, no feedback was provided. After the black arrow indicated the selected option for 0.5 s, participants proceeded directly to the next trial.

#### Experimental stimuli

The experimental stimuli used in the current study were geometric shapes and their combinations. Six different shapes—square, circle, triangle, rhombus, trapezoid and hexagon (Fig. 1a)—were used as the primitives. To create three-cue stimuli, three shapes were selected out of the six, with replacement. This process generated 56 unique combinations of three cues, categorized according to the number of unique cues (NUC) in each combination (Supplementary Fig. 2). Specifically, there were 6 combinations with NUC = 1 (all cues identical), 30 combinations with NUC = 2 (two unique cues with one repeated) and 20 combinations with NUC = 3 (all three cues unique).

In each block of singleton training, where only one cue was presented per trial, each of the six individual cues was repeated 10 times, resulting in 60 trials per block (Supplementary Fig. 2). For parallel training, which involved three cues per trial, each of the 20 combinations with NUC = 3 was repeated 3 times, also yielding 60 trials per block (Supplementary Fig. 2). Test trials, which consisted exclusively of three-cue combinations, were identical across singleton and parallel conditions. Each testing block included 10 trials with NUC = 1 (all 6 combinations, with 4 repeated), 30 trials with NUC = 2 (all 30 combinations) and 20 trials with NUC = 3 (all 20 combinations). This structure ensured consistency in testing conditions across both learning curricula.

### Experiment 2

In Exp. 2, 83 participants (*N* = 41 in the singleton condition and *N* = 42 in the parallel condition; 48 females, 34 males and 1 other; age, 24.2 ± 3.93 yr) were included in the final data analyses after 12 (12.6%) were excluded following the same criteria as in Exp. 1.

Exp. 2 aimed to test whether the advantage on singleton trials could generalize from trained to untrained cue combinations. The participants were trained with only half of the total combinations of three unique cues (10 out of 20 combinations) and then tested on both trained and untrained combinations (all 20 combinations). The overall experimental setup was otherwise identical to Exp. 1.

For each participant, the 20 combinations of three unique cues selected out of six were randomly divided into two groups. The participants were trained using combinations from one group, ensuring that each presented combination occurred twice as often as in Exp. 1. The testing phase was identical to that of the previous experiment, including all cue combinations of three unique cues, regardless of training exposure.

### Experiment 3

In Exp. 3, 52 participants (*N* = 28 in the singleton condition and *N* = 24 in the parallel condition; 25 females, 26 males and 1 other; age, 25.1 ± 3.60 yr) were included in the final data analyses after 13 (20%) participants were excluded following the same criteria as in previous experiments.

Exp. 3 aimed to replicate the singleton effect while testing alternative explanations for this effect. The basic set-ups of Exp. 3 were the same as Exp. 1, but after training and testing on the standard set of cues (set 1), we added two additional blocks of singleton training for both conditions, involving a new set of cues (set 2). In the extended blocks of training, participants predicted whether the temperature would be high or low on the basis of the presented animal head icons (Fig. 2d). Except for the physical appearance of the cues and the cover story, this extended phase was identical in every way to the first two blocks of training with set 1, including the presence of feedback. We compared choice accuracy averaged over these two blocks between conditions.

### Experiment 4

In Exp. 4, 86 new participants (*N* = 44 in the parallel skewed high condition and *N* = 42 in the parallel skewed low condition; 41 females, 44 males and 1 other; age, 24.8 ± 3.39 yr) were included in the data analyses after 25 (22.5%) were excluded following the same criteria as in previous experiments. For the purposes of analysis, these were pooled with data from 244 participants who had participated in Exps. 1–3 (*N* = 119 in the singleton condition and *N* = 125 in the parallel uniform condition) for comparison.

Exp. 4 involved two additional training curricula that were generated using the identified hybrid learning model. In one curriculum, participants were exposed more frequently to three-cue combinations with extreme summed WOE values, referred to as the parallel skewed high condition. In the other curriculum, where the model predicted an impaired learning performance, participants were trained more on three-cue combinations with less extreme summed WOE values, referred to as the parallel skewed low condition. The distributions of summed WOE values in these two conditions are shown in Fig. 4a, and the specific three-cue combinations used in each condition are detailed in Supplementary Data 1. We carefully designed the training stimuli in the skewed conditions to ensure that each cue was presented an equal number of times across both conditions. The basic set-ups for the skewed conditions were the same as for the parallel condition in Exp. 1, with the only exception that the training stimuli in the first four training blocks were modified as described above.

### Experiment 5

In Exp. 5, 52 new participants (idealized parallel condition; 33 females, 19 males and 0 other; age, 24.8 ± 3.60 yr) were used for analyses after 5 (8.8%) were excluded following the same criteria as in previous experiments. These participants were pooled with 83 participants from Exp. 2 (*N* = 41 in the singleton condition and *N* = 42 in the parallel condition) for comparison.

Exp. 5 aimed to investigate whether eliminating the reward stochasticity in parallel training could improve learning performance. To test this, we introduced the idealized parallel condition. In this condition, for the first four training blocks, the rightward choice was always rewarded when the summed WOE value of the three presented cues was greater than 0, and the leftward choice was always rewarded otherwise. Similar to Exp. 2, participants in the idealized parallel condition were trained with only half of the total combinations of three unique cues and tested with all combinations. We chose this design for two reasons: first, to assess whether idealized training would improve performance on the trained combinations, and second, to evaluate whether any improvement in trained combinations would transfer to untrained combinations. The test trials in Exp. 5 were consistent with those in other conditions and kept stochastic, ensuring a meaningful comparison between Exp. 5 and the other experiments. For consistency, we compared the results of Exp. 5 with those of Exp. 2.

### Experiment 6

In Exp. 6, 192 participants (*N* = 47 in the singleton condition, *N* = 47 in the parallel condition, *N* = 48 in the ascending condition and *N* = 50 in the descending condition; 93 females, 95 males and 4 others; age, 24.6 ± 3.76 yr) were included in the final data analyses after 42 (17.9%) were excluded following the same criteria as in the previous experiments.

Exp. 6 aimed to evaluate whether an ascending training curriculum, which begins with simpler trials and progresses to more complex ones, could outperform a descending curriculum, which starts with complex trials and moves to simpler ones. To test this, we introduced two additional training conditions: ascending and descending, along with the singleton and parallel conditions. In the ascending condition, the number of cues per training trial gradually increased over blocks (that is, from one to two to three), while in the descending condition, the number of cues per training trial gradually decreased over blocks (that is, from three to two to one). The training procedures for all four conditions are illustrated in Supplementary Fig. 17a.

The basic set-up for the singleton and parallel conditions remained consistent with Exp. 1, with one modification: two additional training blocks were added to each. Singleton training included two additional blocks of single-cue trials, and parallel training included two additional blocks of three-cue trials. These changes ensured that the total number of training trials matched that in the ascending and descending conditions, allowing for a direct comparison of learning performance. The extended training also tested whether increasing the number of trials in the parallel condition would enhance performance. The testing phase was identical to Exp. 1 and consisted of the same set of trials across all conditions. As a result, participants in Exp. 6 completed a total of 720 experimental trials (compared with 600 in the other experiments), with the entire experiment taking approximately 75 min to complete.

In the ascending and descending conditions, the first six blocks of training (60 trials per block) were identical but presented in reverse order. Specifically, participants in the ascending condition began training with one cue and progressed to two cues and then to three cues. In contrast, participants in the descending condition started with three cues, then moved to two cues and concluded with one cue. Each type of training (one-cue, two-cue and three-cue) was presented in two blocks. After these initial six blocks, participants in both conditions completed two blocks of three-cue trials, followed by four blocks of test trials, consistent across all conditions.

The structure of the one-cue training blocks in the ascending and descending conditions mirrored that of the singleton condition, while the three-cue training blocks followed the same format as the parallel condition. For the two-cue training trials, we generated 15 unique combinations $\left(\genfrac{}{}{0ex}{}{6}{2}\right)$ but excluded 3 combinations where the summed WOE value was 0, leaving 12 valid combinations. Each of these 12 combinations was repeated five times in each training block, resulting in 60 trials per block with two-cue trials.

### Estimation of subjective WOE

Following established methods^20^, we used logistic regressions to estimate participants’ subjective WOE on the basis of their choices in the test trials. Specifically, the logistic regression models predicted the probability of a rightward response as a function of the number of times each cue *i* appeared in a trial, denoted as *N*_*i*_, and the corresponding cue weight, *w*_*i*_:

$$P(\mathrm{choice}=\mathrm{right})=\frac{1}{1+{{e}}^{-{\sum }_{{{i}}=1}^{6}{{{w}}}_{{{i}}}{{{N}}}_{{{i}}}}}$$

The six fitted coefficients *w*_*i*_ represent the subjective WOE for each cue. We performed simulations to demonstrate that this method accurately recovered the underlying objective WOE used to generate the data (Supplementary Fig. 18). For each condition in each experiment, we pooled test trials from all participants and performed a mixed-effects logistic regression, estimating the subjective WOE for each participant by incorporating both the fixed effect and the random effect of each participant.

Two separate methods were employed to compare the subjective WOE across different conditions. First, we performed linear regressions, modelling subjective WOE as a function of the objective WOE, condition and their interaction. This allowed us to test whether there was a significant interaction between the objective WOE and condition in predicting the subjective WOE. Second, we compared the magnitude of subjective WOE across conditions. Specifically, for each participant, we first adjusted the recovered subjective WOE value for each cue by weighting it with the inverse of its squared standard error to account for the estimation reliability. Specifically:

$${w}_{i}^{{\rm{adj}}}={w}_{i}\times \frac{1/{{\rm{s}}.{\rm{e}}.}_{i}^{2}}{{\sum }_{k=1}^{6}1/{{\rm{s}}.{\rm{e}}.}_{k}^{2}}$$

where ${{\rm{s}}.{\rm{e}}.}_{i}^{2}$ is the square of the estimated standard error of *w*_*i*_. We then reversed the sign of ${w}_{i}^{{\rm{adj}}}$ for cues with objective WOE < 0 and summed the sign-adjusted ${w}_{i}^{{\rm{adj}}}$ across all presented cues:

$${w}_{\rm{total}}=\mathop{\sum }\limits_{i=1}^{6}\left({w}_{i}^{\rm{adj}}\times \rm{sign}({\rm{WOE}}_{i})\right)$$

We referred to this quantity as ‘total weight’. The sign adjustment ensures that weights contribute positively when they align with the correct normative direction and negatively when they are in the incorrect direction. We performed independent two-sample *t*-tests to compare the total weight between different conditions (for example, singleton versus parallel).

### Calculation of empirical WOE

We computed the empirical WOE for each cue on the basis of the reward probability conditioned on the presence of that cue in a training trial. Specifically, for each cue, we aggregated all training trials to determine the probability that a rightward choice would be rewarded when the cue was present. This probability, *P*(*r*|*i*), was calculated as follows:

$$P({r|i})=\frac{{\sum }_{k=1}^{N}{\delta }_{k}(r)\times {1}_{{s}_{k}}(i)/N({s}_{k})}{{\sum }_{k=1}^{N}{1}_{{s}_{k}}(i)/N({s}_{k})}$$

*N* is the total number of training trials. *δ*_*k*_(*r*) is an indicator function for whether the rightward choice in trial *k* is rewarded (*δ*_*k*_(*r*) = 1 if rewarded; otherwise, *δ*_*k*_(*r*) = 0). ${1}_{{s}_{k}}(i)$ is an indicator function that equals 1 if cue *i* is included in the stimulus *s*_*k*_ for trial *k*; otherwise, it equals 0. *N*(*s*_*k*_) represents the total number of cues presented in the stimulus *s*_*k*_, which adjusts the relative contribution of the cue to the conditional reward probability by accounting for the number of cues in one trial.

After calculating *P*(*r*|*i*), we transformed it into the empirical WOE (eWOE_*i*_) by computing the log-likelihood ratio of the conditional reward probability:

$${\rm{eWOE}}_{i}=\log \left(\frac{P(r|i)}{1-P(r|i)}\right)$$

This transformation captures the conditional strength of evidence provided by each cue in determining the likelihood of a reward.

### Computational modelling

Following previous studies^31,32,70^, we employed a single-layer perceptron to model the learning process in this study. This model, as illustrated in Fig. 3a, consists of an input layer (*x*_*i*_) that encodes the presented cues and an output layer (*y*_*j*_) representing the probability of selecting action *j*. The model frames the learning of evidence associated with each cue in the weather prediction task as a reinforcement learning problem, a framework that has been successfully applied in prior studies.

The activity of each input node (*x*_*i*_) corresponds to the presence of cue *i* in a trial: *x*_*i*_ = 0 if cue *i* is absent, *x*_*i*_ = 1 if only one copy of cue *i* is present and *x*_*i*_ = 2 if two copies of cue *i* are present. The model computes the integrated activity from the input nodes to each output node:

$${W}_{j}=\mathop{\sum }\limits_{i=1}^{6}{w}_{{ij}}{x}_{i}$$

*w*_*ij*_ is the connection weight between input node *i* and output node *j*. The probability of selecting option *j* (*y*_*j*_) is given by a softmax function of the integrated activity:

$${y}_{j}=\frac{{{\rm{e}}}^{{\beta W}_{j}}}{{\sum }_{u=1}^{2}{{\rm{e}}}^{\beta {W}_{u}}}$$

where *β* is the inverse temperature parameter, which reflects the stochasticity of participants’ choices relying on the integrated evidence. *β* is allowed to be different in single-cue and multi-cue trials to account for the cost of evidence integration from one cue to multiple cues (see Supplementary Fig. 7c,d for a comparison of models with and without integration cost). In the current study, we use *β*_1_ and *β*_3_ to refer to the inverse temperature for single-cue and multi-cue trials, respectively. For practical purposes, we fixed *β*_3_ to be 1 and only estimated *β*_1_ during model estimation to account for the possible collinearity between *β*_1_ and *β*_3_ if they were both allowed to change. Therefore, a larger *β*_1_ means a greater cost in evidence integration from single cue to multiple cues.

After feedback is provided, the connection weights (*w*_*ij*_) are updated using a gradient descent rule (for test trials without feedback, the weights are no longer updated):

$${w}_{{ij}}={w}_{{ij}}+\alpha \times {\delta }_{{ij}}{{{\times}}x}_{i}$$

Here, *α* is the learning rate, allowed to be different for positive and negative outcomes (*α*_+_ and *α*_−_, respectively; see also Supplementary Fig. 7a,b for a comparison of models with and without different learning rates for different outcomes). *δ*_*ij*_ represents the PE signal for each presented cue given the chosen option *j*, which is computed as the difference between the observed outcome *r* and the expectation *y*_*j*_:

$${\delta }_{{ij}}=r-{y}_{j}$$

The choice outcome (*r*) equals 1 if the selected option is rewarded and 0 otherwise. The expectation of *y*_*j*_ might be different depending on the specific learning strategy (see details below).

The model-learned WOE for each cue *i* is then calculated as the difference in weights associated with the two choice options:

$${{\rm{WOE}}}_{i}={w}_{i2}-{w}_{i1}$$

#### Model 1: marginal updating model

In marginal updating, each cue is assumed to form a direct association with the outcome, independent of other cues presented on the same trial (Supplementary Fig. 6b (left)). Accordingly, the PE for cue *i* is determined solely by its own prediction:

$${\delta }_{{ij}}^{M}=r-{y}_{{ij}}$$

where *y*_*ij*_ is the predicted reward probability for option *j* based on cue *i* alone:

$${y}_{{ij}}=\frac{{{{e}}}^{{\beta }_{1}{w}_{{ij}}}}{{\sum }_{u=1}^{2}{{{e}}}^{{\beta }_{1}{w}_{{iu}}}}$$

Here, *w*_*ij*_ represents the weight linking cue *i* to option *j*, and *β*_1_ is the inverse temperature governing choice stochasticity in single-cue trials. Because each cue generates its own prediction, the marginal updating PEs may differ across cues within a trial. This model contains three free parameters (*α*_+_, *α*_−_ and *β*_1_), with *β*_3_ fixed at 1.

Marginal updating is computationally efficient, as it avoids the integration of evidence across multiple cues, which consumes cognitive efforts. However, this efficiency comes at the cost of accuracy: when learning converges, the estimated WOE for each cue is systematically attenuated relative to the objective WOE values. This bias reflects the marginal reward probability of each cue given participants’ learning history, rather than the true causal influence of the cue (Supplementary Fig. 6c,d).

#### Model 2: joint updating model

In joint updating, cue weights are updated on the basis of the integrated evidence from all cues presented on a trial, rather than from each cue in isolation (Supplementary Fig. 6b (right)). The PE for cue *i* is therefore determined by the observed outcome relative to the prediction derived from the combined evidence of all cues:

$${\delta }_{{ij}}^{\rm{J}}=r-{y}_{j}$$

where *y*_*j*_ is the predicted reward probability for option *j* based on the integrated evidence of all presented cues, given by:

$${y}_{j}=\frac{{{{e}}}^{\beta {W}_{j}}}{{\sum }_{u=1}^{2}{{{e}}}^{\beta {W}_{u}}}$$

where *β* is the inverse temperature parameter, set to *β*_1_ for single-cue trials and set to *β*_3_ for multi-cue (three-cue) trials. *W*_*j*_ represents the integrated evidence for option *j*:

$${W}_{j}=\mathop{\sum }\limits_{i=1}^{6}{w}_{{ij}}{x}_{i}$$

Because the PE is derived from the integrated prediction, all presented cues share the same error signal. The joint updating model estimates three free parameters (*α*_+_, *α*_−_ and *β*_1_) in the singleton condition and two free parameters (*α*_+_ and *α*_−_) in the parallel condition, with *β*_3_ fixed at 1.

Relative to marginal updating, joint updating is assumed to be more cognitively demanding, as it requires evidence integration from multiple cues for weight updating. However, its advantage lies in learning precision: when learning converges, the model ensures that participants’ estimated WOE values match the true objective WOEs (Supplementary Fig. 6c).

#### Model 3: hybrid constant model

Hybrid learning models assume that participants rely on a mixture of marginal and joint updating, rather than adopting a single strategy. In the hybrid constant model, the contribution of each strategy remains constant throughout training. In particular, the PE signal of this model is computed as a weighted average of the marginal and joint updating PEs, with a fixed weighting parameter *γ*:

$${\delta }_{{ij}}=(1-\gamma )\times {\delta }_{{ij}}^{{\rm{M}}}+\gamma \times {\delta }_{{ij}}^{{\rm{J}}}$$

where ${\delta }_{{ij}}^{{\rm{M}}}$ and ${\delta }_{{ij}}^{{\rm{J}}}$ denote the PEs for marginal and joint updating, respectively. The free parameter *γ* is constant across all training trials, reflecting the relative weight assigned to joint updating. When *γ* = 0, the model reduces to pure marginal updating, and when *γ* = 1, it reduces to pure joint updating.

This form of hybrid updating, with a constant weighting parameter, has been widely reported in previous work^36,37^. The model includes four free parameters (*α*_+_, *α*_−_, *β*_1_ and *γ*), with *β*_3_ fixed at 1 in both the singleton and parallel conditions.

#### Model 4: hybrid PE model

The hybrid PE model allows the weighting parameter *γ* to vary dynamically depending on the discrepancy between marginal and joint updating PEs. Specifically, *γ* is modelled as an increasing logistic function of the distance between the two error signals, such that larger discrepancies make joint updating more likely. For simplicity, we implemented an extreme case in which participants use marginal updating when the PE distance is below a threshold and switch to joint updating otherwise. Formally, for each cue *i*, the PE *δ*_*ij*_ is given by:

$$\begin{array}{l}{\delta }_{{ij}}=\left\{\begin{array}{l}{\delta }_{ij}^{{\rm{M}}},\,{\mathrm{if}}\,\left|{\delta }_{j}^{{\rm{M}}}-{\delta }_{j}^{{\rm{J}}}\right|\le \theta \\ {\delta }_{ij}^{{\rm{J}}},\,{\mathrm{if}}\,\left|{\delta }_{j}^{{\rm{M}}}-{\delta }_{j}^{{\rm{J}}}\right| > \theta \end{array}\right.\end{array}$$

where ${\delta }_{j}^{{\rm{M}}}$ and ${\delta }_{j}^{{\rm{J}}}$ denote the sets of PEs under marginal and joint updating, respectively. For example, in three-cue trials, ${\delta }_{j}^{{\rm{M}}}$ represents the set of the marginal updating PE signals of the three cues.

$|{\delta }_{j}^{{\rm{M}}}-{\delta }_{j}^{{\rm{J}}}|$ indicates the Euclidian distance between PEs generated by marginal and joint updating. The threshold *θ* is a free parameter that reflects the relative cognitive cost of implementing joint updating compared to marginal updating and is hypothesized to be larger than 0 as joint updating is assumed to be more cognitively demanding. In total, this model includes four free parameters (*α*_+_, *α*_−_, *β*_1_ and *θ*), with *β*_3_ fixed at 1 in both conditions.

This model is inspired by the resource-rationality framework, which explains human cognition as the outcome of balancing the expected reward and cognitive effort and has been successfully used to model human higher-order cognition (for example, planning, reasoning, communication and so on)^33–35,71^. From this perspective, learners should select the strategy that maximizes expected utility under resource constraints. Here, marginal updating is computationally simpler but less precise, leading to biased WOE estimates. Joint updating, in contrast, recovers the objective WOE values but at higher cognitive cost. Therefore, participants are assumed to arbitrate between these two strategies by comparing their differences in learning precision with the relative cognitive cost. If the gain in learning precision for joint updating is greater than its additional cost in cognitive demand relative to marginal updating, then a rational learner should be more likely to select the joint updating; otherwise, they should choose marginal updating.

The gain in learning precision for joint updating relative to marginal updating is quantified as the distance between the PEs generated by the two learning strategies. A larger PE distance means that marginal updating incurs a greater loss in learning precision than joint updating. The additional cost in cognitive demand is quantified as a free parameter *θ*, which is estimated individually and represents the point at which the gain in learning precision from joint updating outweighs its additional cognitive cost, making joint updating the rational choice.

However, the hybrid PE model also raises a conceptual limitation. To evaluate the PE distance, learners would need to compute the joint updating PEs before deciding whether to use them—an arguably inefficient process. A more plausible account is that participants approximate this evaluation heuristically rather than explicitly computing both sets of PEs. Such considerations motivate alternative models, such as the hybrid s.d. model (described below), which relax this assumption while retaining the core idea of resource-rational arbitration between strategies.

#### Model 5: hybrid s.d. model

The hybrid s.d. model provides a heuristic approximation of the hybrid PE model by quantifying the relative difference in learning precision with the variability in cue weights. Specifically, this model assumes that participants assess the s.d. of the WOE values across all presented cues in each trial. When the s.d. exceeds a threshold *θ*, participants are assumed to use joint updating; otherwise, they rely on marginal updating. Formally, for each cue *i*, the PE *δ*_*ij*_ is given by:

$$\begin{array}{l}{\delta }_{{ij}}=\left\{\begin{array}{l}{\delta }_{ij}^{{\rm{M}}},\,\mathrm{if}\,{\rm{s}}.{\rm{d}}.(\mathrm{WOE})\le \theta \\ {\delta }_{ij}^{{\rm{J}}},\,\mathrm{if}\,{\rm{s}}.{\rm{d}}.(\mathrm{WOE}) > \theta \end{array}\right.\end{array}$$

where s.d.(WOE) denotes the standard deviation of the learned subjective WOE values across all presented cues. This measure is highly correlated with the PE distance used in the hybrid PE model (Spearman’s *ρ* = 0.96; Supplementary Fig. 6g) but is more straightforward to compute. This model contains four free parameters (*α*_+_, *α*_−_, *β*_1_ and *θ*), with *β*_3_ fixed at 1 in both conditions.

Evidence from prior work suggests that individuals’ decisions are influenced not only by the magnitude of available evidence but also by its variability^30,72,73^. At the neural level, representations of stimulus variance have also been observed during decision-making^73–75^. These findings lend support to the idea that learners may use the variability of cue weights as a proxy for the difference in learning precision. Consequently, although the hybrid s.d. model is less mathematically rigorous than the hybrid PE model, it may better capture the heuristics learners use in practice and therefore offer a more cognitively plausible account.

#### Model parameters in Exp. 6

For the ascending and descending conditions, we simplified model estimation by using the same number of parameters as in the singleton and parallel conditions. Although participants were trained with one-cue, two-cue and three-cue trials in these two conditions, we assumed identical positive and negative learning rates across all trial types, regardless of the number of cues presented. Likewise, the inverse temperature for two-cue trials was set equal to that for three-cue trials. Importantly, relaxing these assumptions—for example, allowing different learning rates for different trial types—did not alter the conclusion that the hybrid learning model better accounted for participants’ behaviour than single-strategy models.

### Alternative models

#### Models with distinct learning rates for single- and multi-cue trials

In this class of models, the learning rates for single-cue trials and multi-cue trials are allowed to differ. Specifically, in the singleton condition, four learning rates are implemented, with two for single-cue trials (that is, *α*_1,+_ and *α*_1,−_) and two for three-cue trials (that is, *α*_3,+_ and *α*_3,−_). Compared with models without distinct learning rates for different learning contexts, this model introduces two additional parameters.

#### Models with decay in learning rates

This class of models is identical to the original formulations except that the learning rates are assumed to gradually decline over the course of training. Specifically, the positive learning rate at trial *t* is defined as:

$${\alpha }_{+,t}={\alpha }_{+,0}\times {{{e}}}^{-\eta \times t}$$

where *α*_+,0_ is a free parameter representing the initial positive learning rate, and *η* is a free parameter capturing the rate of decay. The trial index is denoted by *t*, starting from 0. The negative learning rate is then computed analogously:

$${\alpha }_{-,t}={\alpha }_{-,0}\times {{{e}}}^{-\eta \times t}$$

where *α*_−,0_ represents the initial negative learning rate. Compared with models without decay, this class of models introduces an additional parameter (*η*) in both conditions.

#### Models with attentional weights

This class of models extends the original framework by introducing an attentional weight parameter for each cue to capture potential biases in attention. Following prior work^48^, attentional weights are incorporated at both the choice valuation and learning stages. Specifically, the integrated evidence for option *j* is computed as:

$${W}_{j}=\mathop{\sum }\limits_{i=1}^{6}{\varphi }_{i}{{\times }}{w}_{{ij}}{{\times }}{x}_{i}$$

where *φ*_*i*_ represents the attentional weight assigned to cue *i*. Similarly, after getting feedback, the weight associated with each cue *i* is updated by:

$${w}_{{ij}}={w}_{{ij}}+\alpha \times {\varphi }_{i}\times {\delta }_{{ij}}{\times x}_{i}$$

We also examined variants in which attentional weights were applied only at the choice valuation stage or only at the learning stage. These alternatives produced results equivalent to those obtained when attentional weights were included at both stages. For clarity, we therefore reported only the results from the latter implementation. Compared with models without attentional bias, this class of models introduces six additional parameters in both conditions.

### Simulations for marginal and joint updating

To assess the learning performance under different strategies, we simulated participants who relied exclusively on either marginal or joint updating during one-cue and three-cue training trials, respectively. These simulations were used to illustrate why marginal updating provides a lower learning precision than joint updating and were distinct from likelihood-based, trial-by-trial modelling used for inference. The procedure was consistent across trial types and employed the same parameter set. For each trial type, we simulated 50 participants over 100 training epochs. Training trials in each epoch matched those used in the empirical experiments (that is, the first four blocks of the singleton and parallel conditions).

The initial weight for the connections between each input node and each output node was set to 0. The learning rate (*α*) was set to 0.01 for both positive and negative outcomes and for all simulations. To ensure convergence, we monitored the loss, calculated as the mean squared error between model predictions and actual results. Simulations were considered converged if the loss showed no significant decrease. All simulations converged within 80 epochs. We then averaged the results from the final 10 epochs (91 to 100 epochs) to obtain the learned WOE values for each cue under each training regime.

The simulated results are presented in Supplementary Fig. 6c. Joint updating consistently recovered the objective WOE values of each cue regardless of the training contexts. By contrast, marginal updating successfully recovered objective WOE values only in one-cue training, while multi-cue training produced systematic deviations. In these cases, the WOE values learned by marginal updating reflected the marginal reward probability of each cue given the training history rather than the true objective values.

### Model estimation and comparison

To evaluate whether the hybrid learning models better explained participants’ behaviour than single-strategy models, we fitted all candidate models to each individual’s choices for all experimental conditions. Parameters were estimated using maximum likelihood estimation, which identified the set of values that maximized the log-likelihood (LL) of the observed choices:

$${\mathrm{LL}}=\mathop{\sum }\limits_{t=1}^{T}{\log}\,({y}_{c,t})$$

where *y*_*c*,*t*_ denotes the model-predicted probability of making choice *c* at trial *t*, and *T* is the total number of trials, including both training and test trials.

The initial parameter values before estimation were randomly sampled from predefined distributions: (1) the positive and negative learning rates (*α*_+_ and *α*_−_) from a uniform distribution on [0, 1], (2) the inverse temperature *β*_1_ from [0, 10], (3) the weight parameter *γ* in the hybrid constant model from [0, 1], (4) the cognitive cost threshold *θ* from [0, 1] in the hybrid PE model and from [0, 2.65] in the hybrid s.d. model, (5) the learning rate decay parameter *η* from [0, 0.5], and (6) the attentional weight parameter *φ* from [0, 1]. These ranges also defined the bounds on parameter values during estimation. To reduce the risk of local minima, we repeated the sampling-and-fitting procedure 500 times for each participant and each model, retaining the parameter set with the highest log-likelihood.

Model comparison was performed using the AIC value, which balances model fit against model complexity and has been widely used in previous studies. For each model:

$${\rm{AIC}}=-2\times {\rm{LL}}+2K$$

where *K* is the number of free parameters. Lower AIC values indicate a better account of behaviour. We compared AIC values across models using within-participant *t*-tests or repeated-measures analysis of variance to test whether the hybrid learning model has a smaller AIC value than the single-strategy models. We also compared models using the Bayesian information criterion (BIC), which more strongly penalizes model complexity. Specifically, for each model,

$${\rm{BIC}}=-2\times {\rm{LL}}+K\times {\rm{ln}}(n)$$

where *n* is the total trial number for each participant.

### Model simulations for Exps. 4–6

To test the generalizability of our modelling framework, we simulated participants’ learning performance in Exps. 4–6 using the hybrid s.d. model together with the parameters estimated from Exps. 1–3. This approach provided a stringent out-of-sample prediction, as both the model and parameter values were derived entirely from different participants and training curricula. To reduce the influence of the stochasticity when simulating the model performances under new curricula, we repeated each simulation 200 times with the same set of parameters and calculated the mean as the results.

In Exp. 4, performance in the skewed high and skewed low curricula was simulated using the median of the parameter values estimated from the parallel condition in Exps. 1–3. For example, the positive learning rate was set to the median of the corresponding estimates across all participants in the parallel condition of Exps. 1–3. As a baseline comparison, performance in singleton and parallel conditions was simulated using the median parameters estimated from the corresponding conditions in Exps. 1–3.

In Exp. 5, performance under the idealized parallel curriculum was simulated using parameters estimated from the parallel condition in Exps. 1–3. In Exp. 6, performance across all curricula was simulated using parameters estimated from both the singleton and parallel conditions in Exps. 1–3. For these simulations, the same positive and negative learning rates were applied across all trial types, regardless of the number of cues presented. Additionally, the inverse temperature for both two-cue and three-cue trials was fixed at 1.

### Pointer game

#### Participants

A total of 93 participants were included in the final analyses (singleton: *N* = 44; parallel: *N* = 49; 49 females and 44 males; age, 31.9 ± 9.45 yr) after 13 participants (12.3%) were excluded who met at least one of the following criteria: (1) had no response in more than 15% of trials in a block, (2) had an average RT below 500 ms in a block or (3) reported issues with stimulus display. The results were robust to these exclusions and were unchanged when all participants were retained.

#### Task

The participants completed a Pointer game, learning how picture symbols moved an avatar along a 21-grid horizontal track (coordinates −10 to 10; Supplementary Fig. 5a). Each symbol represented one arithmetic operation that moved the avatar from one position to another (for example, ‘+1’ moves the avatar from *n* to *n* + 1; Supplementary Fig. 5b). Operations were applied sequentially: the output of one symbol served as the input of the next. On each trial, the avatar started at a random position on the track, and one or three symbols appeared (by condition). The participants needed to report the final location of the avatar after the operations, by clicking the corresponding grid within 10 s. Failure to respond within the time limit triggered a time-out message before the next trial.

After each response, the chosen grid turned grey for 1 s. In the meantime, an orange arrow briefly connected the starting and reported locations. Feedback was then provided for the training trials for 2.5 s: the selected grid turned green if correct or red if incorrect, with the true target location highlighted in green. For test trials, no feedback about the correctness was provided.

The participants were informed that payment depended on performance in both training and testing. They earned £0.01 per correct response on a randomly selected half of all trials, in addition to a completion fee of approximately £10 per hour.

#### Conditions

The participants were randomly assigned to either a singleton or a parallel condition, differing only in the number of symbols shown per trial during the initial training phase (Supplementary Fig. 5c,d).

In the singleton condition, participants first completed three blocks of single-symbol training (32 trials per block; 96 trials total). They then performed one block of three-symbol training (32 trials) to practice sequential application of operations. Finally, they completed two test blocks of three-symbol trials without feedback (128 trials total).

In the parallel condition, participants completed four blocks of three-symbol training (32 trials per block; 128 trials total), with no single-symbol phase. The testing phase matched the singleton condition: two blocks of three-symbol trials without feedback (128 trials total).

#### Experimental stimuli

For each participant, four symbols were sampled at random from a set of 12 and served as primitives. Each selected symbol was randomly assigned one of the four arithmetic operations without replacement.

In each block of one-symbol training, each of the four individual symbols appeared eight times, resulting in 32 trials per block. In each of the three-symbol training blocks, we randomly sampled eight three-symbol sequences from the 24 possible permutations of three unique symbols (4 × 3 × 2) and repeated each sequence eight times (32 trials per block). Test trials consisted exclusively of three-symbol sequences and were identical across the singleton and parallel conditions. Each test block contained all the 64 three-symbol sequences, generated with replacement by four symbols (4 × 4 × 4).

### Reporting summary

Further information on research design is available in the Nature Portfolio Reporting Summary linked to this article.

## Supplementary information

Supplementary Information Supplementary Figs. 1–18 and Table 1.

Reporting Summary

Peer Review File

Supplementary Data 1 Three-cue combinations used in skewed high and low conditions in Exp. 4.

## Data Availability

The processed, anonymized human data in this study are available via the Open Science Framework at https://osf.io/gxv4z. There are no restrictions on data availability, and all relevant data files are provided in CSV format. No data with mandated deposition are included in this study.

## Code availability

The code used for the core data analysis in this study is available via the Open Science Framework at https://osf.io/gxv4z.
