# Supplementary material for: Human curriculum learning of a cue combination task
Source: Nat Hum Behav. 2026 May 5;10(8):1539–56. doi: 10.1038/s41562-026-02452-1 (PMC13485681; doi:10.1038/s41562-026-02452-1)
Supplement: Supplementary file 1 [file 41562_2026_2452_MOESM1_ESM.pdf]

nature human behaviour

Supplementary information https://doi.org/10.1038/s41562-026-02452-1

Human curriculum learning of a cue combination task

In the format provided by the authors and unedited

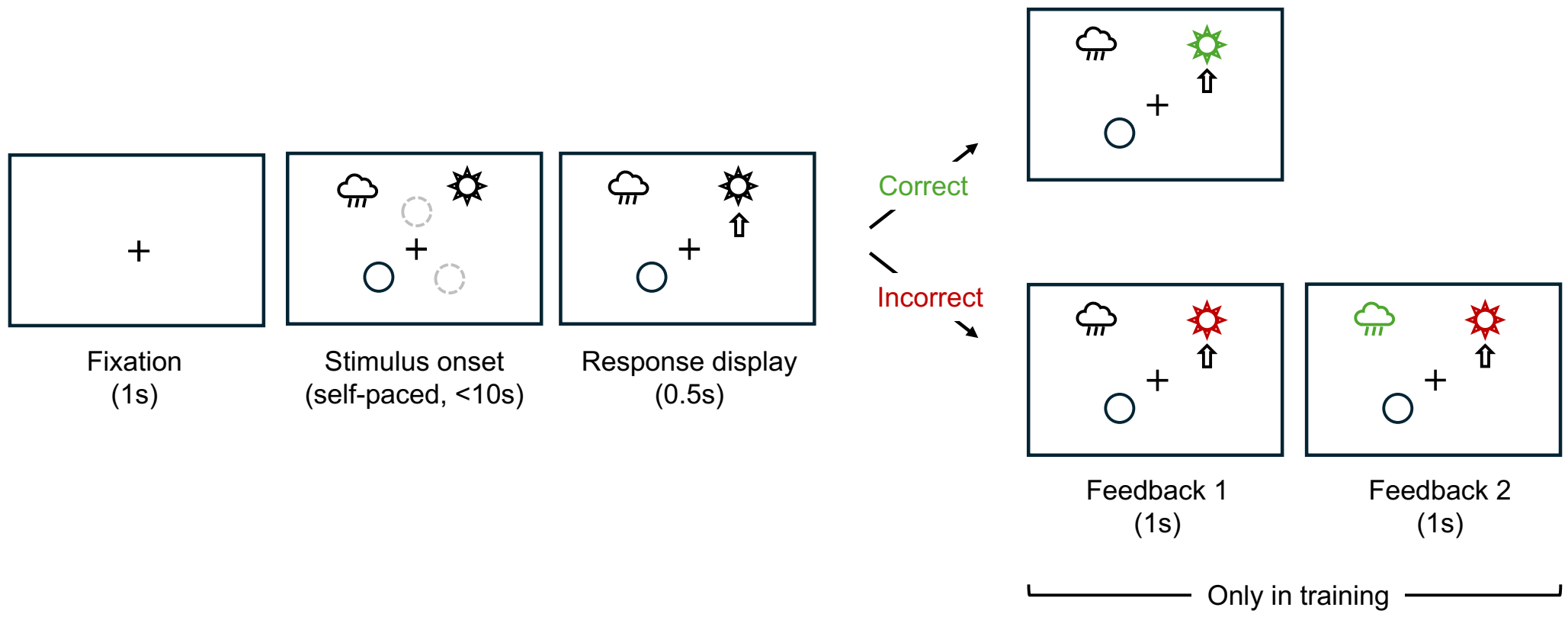

Fig. S1. **Time course of a trial during training and testing stages.** During feedback of training trials, the time course varies depending on whether the participant's prediction is correct or not. No feedback is provided during test trials. This example represents a singleton training trial. The two dashed circles shown at the stimulus onset phase in the figure indicate the other potential locations where the cue could appear. The two dashed circles are for illustration only and would not be displayed in actual experiments. For trials with three cues, the cues are randomly presented at the three locations around the central fixation in each trial.

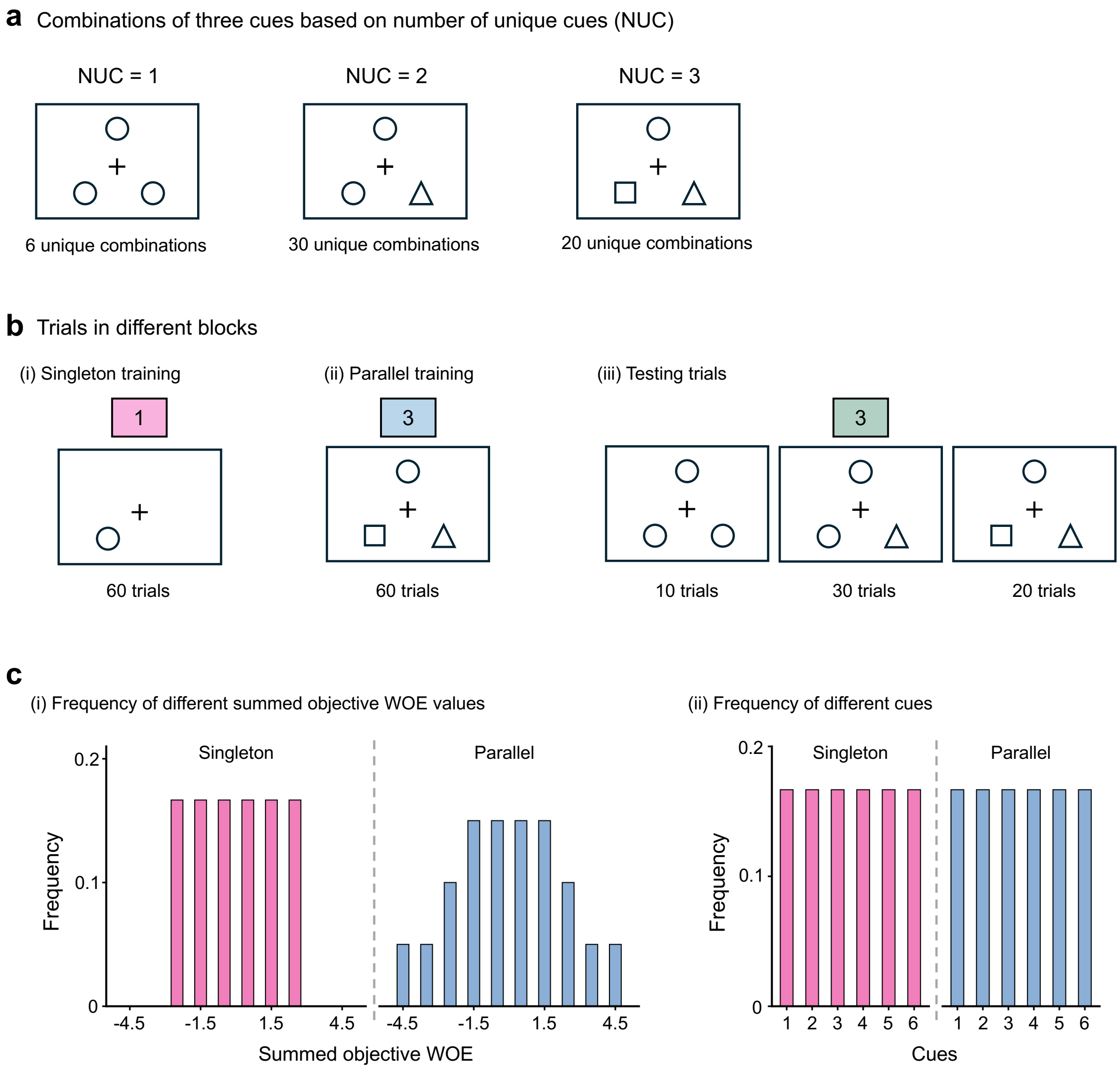

Fig. S2. Experimental stimuli in singleton and parallel conditions. (**a**) Examples of three cue combinations with 1, 2 and 3 unique cues, respectively. The number below each example indicates the total unique combinations for the corresponding trial type. (**b**) Trial structures in different training and testing blocks. The left, middle and right panels depict a block of singleton training trials, parallel training trials, and test trials, respectively. The number below each panel represents the count of each trial type within the corresponding block. For instance, in the right panel (testing block), a testing block includes 10 trials with 1 unique cue (NUC = 1), 30 trials with 2 unique cues (NUC = 2), and 20 trials with 3 unique cues (NUC = 3). (**c**) Distribution of summed objective WOE values and cue frequencies for singleton and parallel training. Left panel: The distribution of the summed objective WOE in training trials for singleton and parallel conditions, respectively. Right panel: The distribution of the frequency of each cue appearing in training trials for the two conditions.

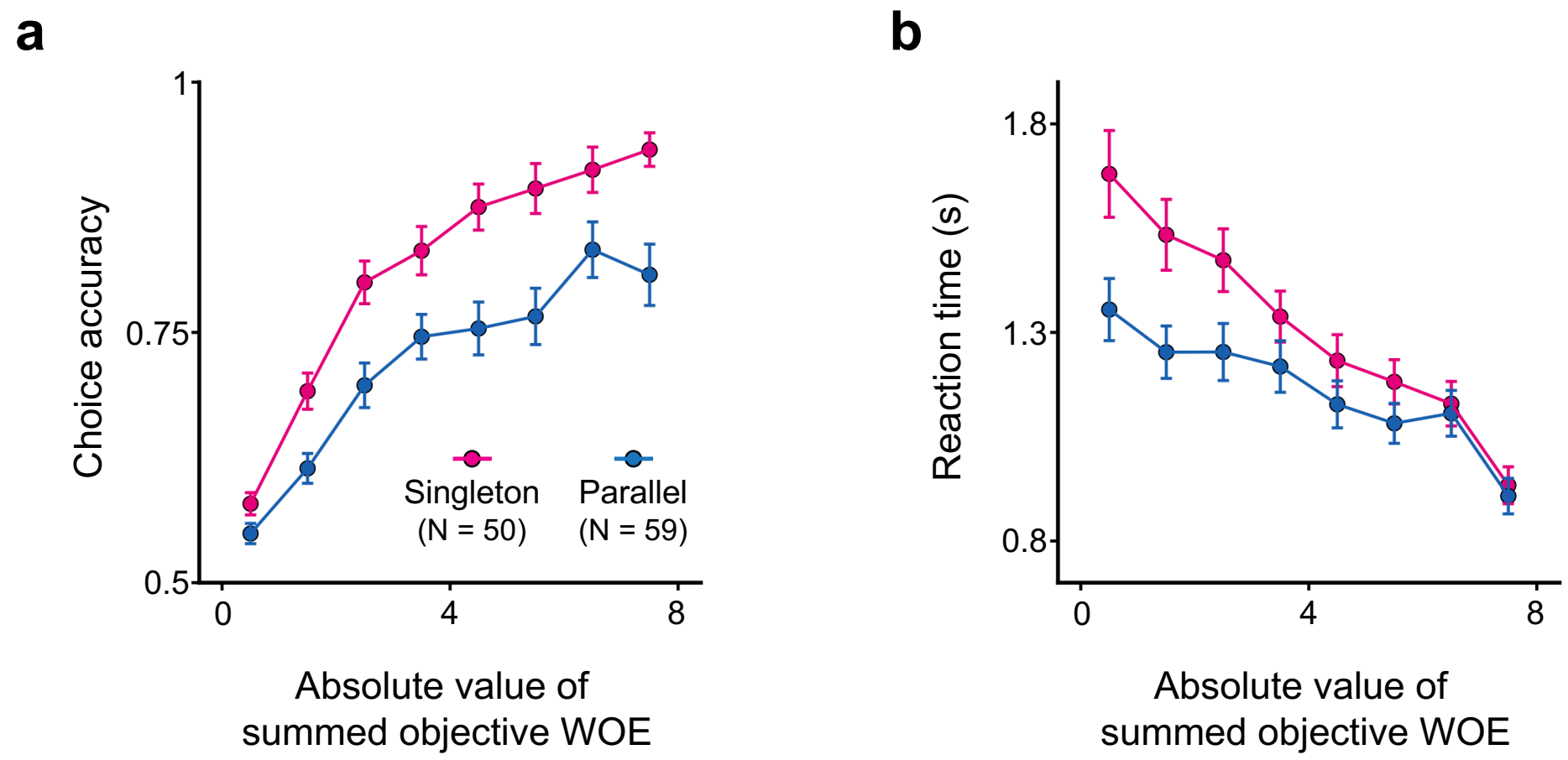

Fig. S3. Choice accuracy and reaction time in test trials of Exp. 1. (**a**) Choice accuracy and (**b**) reaction time plotted as a function of the absolute value of summed objective WOE across all cues presented per trial. Participants trained under the singleton condition showed higher choice accuracy than those trained under the parallel condition, even when the absolute evidence provided by the cues was matched ($z = 3.73$, $p < 0.001$, $\beta = 1.00$, 95% CI 0.48−1.52). Reaction times during testing were comparable across the two training conditions when task difficulty was accounted for ($t(105.3) = 0.29$, $p = 0.772$, $\beta = -0.02$, 95% CI -0.14−0.10), indicating that differences in choice accuracy were not solely driven by response speed. The error bars around the points indicate the standard error of the mean (SEM).

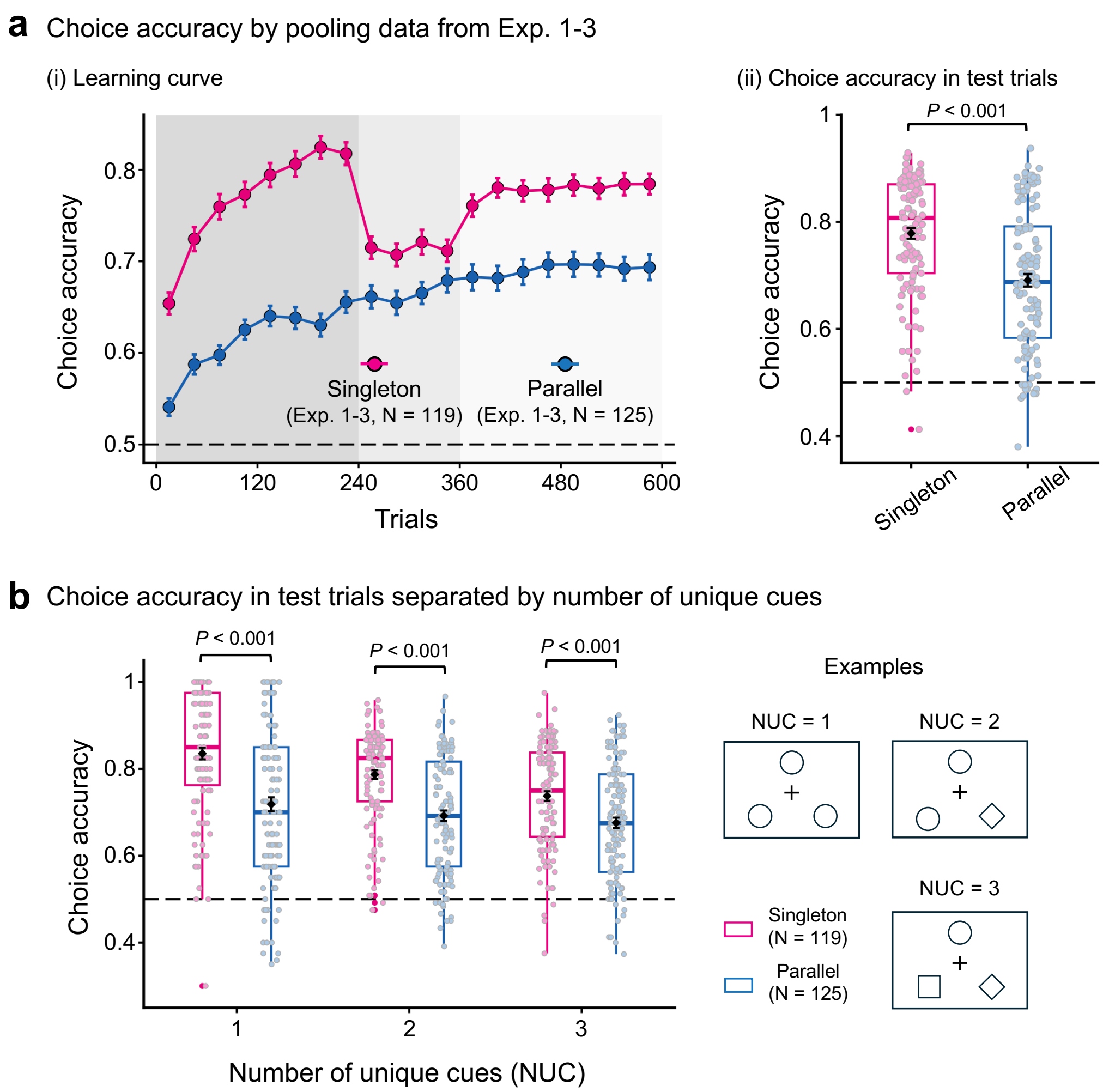

Fig. S4. Empirical results pooled from Exp. 1-3. (**a**) Choice accuracy in training and test trials. Left: Mean choice accuracy over the course of training and testing, shown separately for the singleton and parallel conditions. Each point reflects accuracy averaged over 30 consecutive trials per participant and then across participants within a condition. Error bars denote the SEM. Light grey, grey, and dark grey shaded areas indicate different task stages (240, 120, and 240 trials, respectively; see **Fig. 1c**). Right: Mean choice accuracy across all test trials, with each point representing one participant. (**b**) Choice accuracy by number of unique cues (NUC). Boxplots display mean accuracy across test trials, grouped by the number of unique cues presented per trial. For all box plots in the Supplementary Information, the central line denotes the median, and the bottom and top edges of the box indicate the 25th and 75th percentiles within a group. Whiskers extend to the minimum and maximum values within 1.5× the interquartile range from the box limits (or to the data extrema if no outliers are present). Points beyond the whiskers are shown as individual outliers.

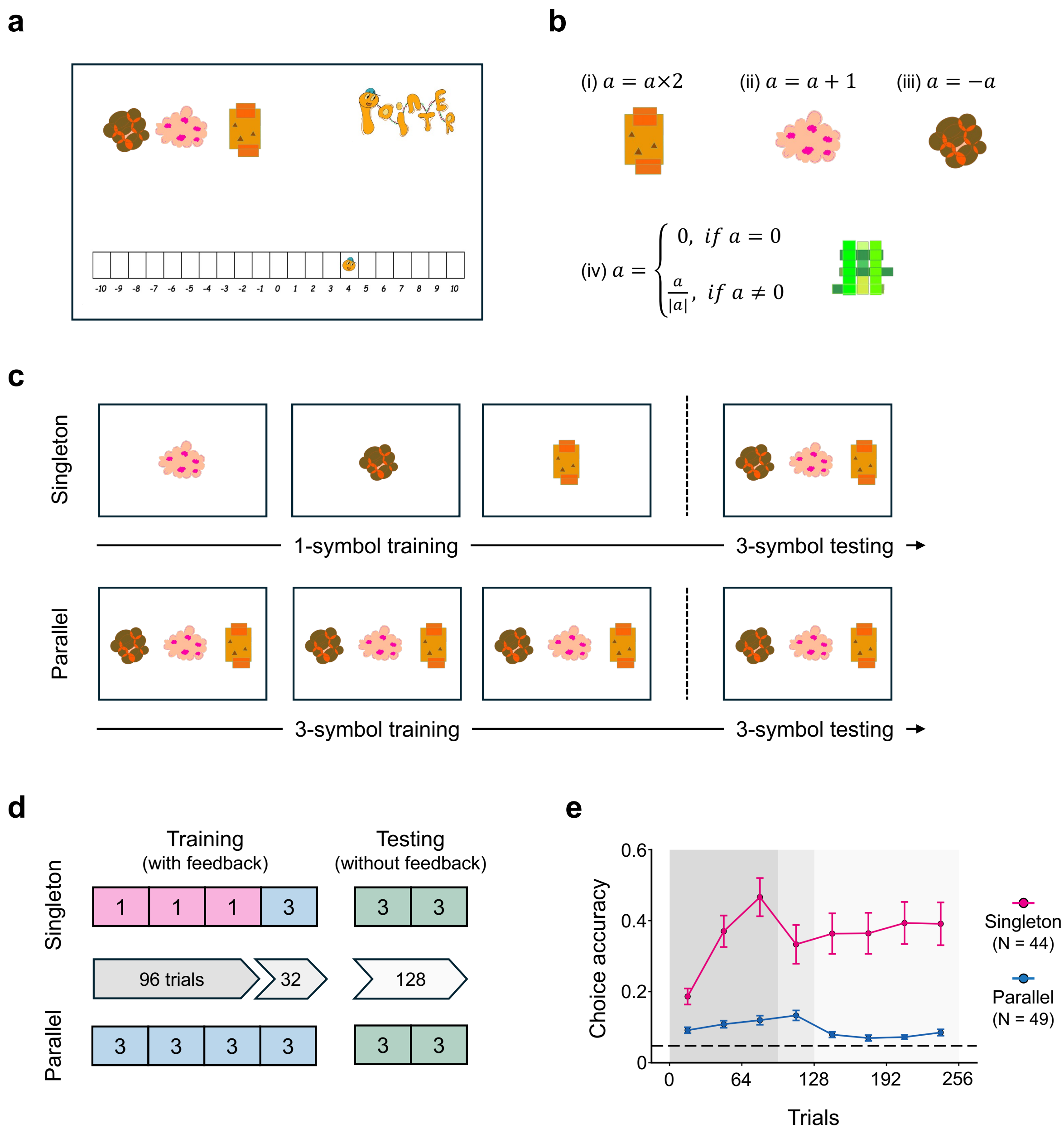

Fig. S5. Generalization of the singleton effect to another compositional task. (**a**) In pointer game, participants reported the final position of an avatar on a one-dimensional track, given a random starting position and a sequence of 'magic' picture symbols. Each symbol denoted an arithmetic operation that could change the location of the avatar. For a sequence of symbols, the output of the first symbol served as the input to the next. In this example, the final position of the avatar is -6 (intermediate position: -4, then -3, then -6 after the first, the second and the third symbol, respectively). (**b**) Arithmetic operations used in this study. For each participant, four symbols were sampled randomly from a set of 12; symbol–operation mappings were randomized across participants. (**c**) Illustration for the curricula. In the singleton condition, participants were trained with trials containing a single symbol during the initial training, whereas in the parallel condition, training involved trials with three symbols. Test trials, which included three symbols,

were identical across conditions. (**d**) Training procedure for singleton and parallel conditions. The two conditions differed only in the initial training phase. Numbers in cells indicate the number of symbols shown per trial. (**e**) Mean choice accuracy across training and testing for each condition. Each point reflects accuracy averaged over 32 consecutive trials per participant and then across participants within a condition. Error bars denote the SEM. Light grey, grey, and dark grey shaded areas indicate different task stages in (**d**).

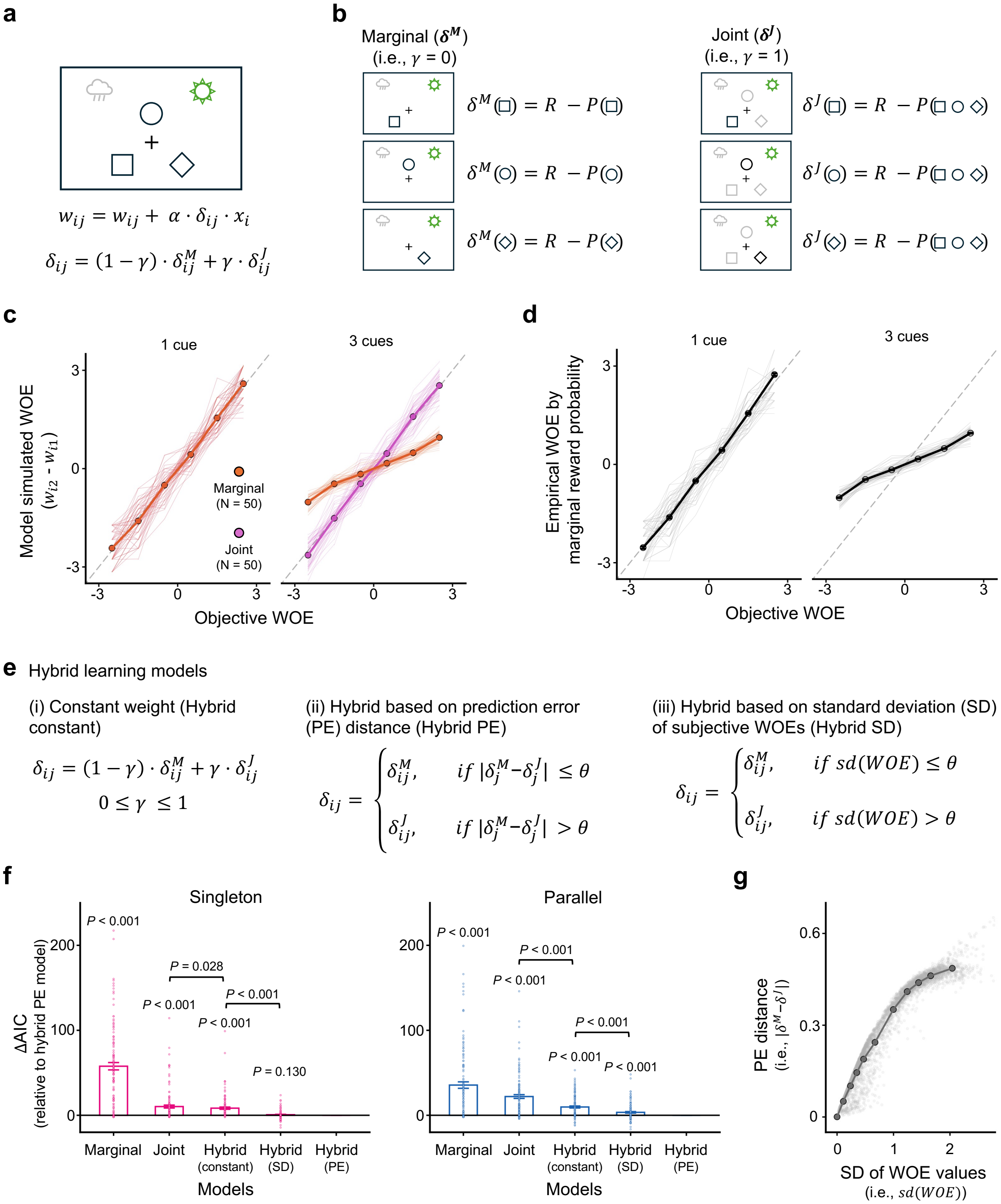

$$w_{ij} = w_{ij} + \alpha \cdot \delta_{ij} \cdot x_i$$

$$\delta_{ij} = (1-\gamma) \cdot \delta_{ij}^M + \gamma \cdot \delta_{ij}^J$$

$$\delta^M(\square) = R - P(\square) \qquad \delta^J(\square) = R - P(\square \bigcirc \diamond)$$

$$\delta^M(\bigcirc) = R - P(\bigcirc) \qquad \delta^J(\bigcirc) = R - P(\square \bigcirc \diamond)$$

$$\delta^M(\diamond) = R - P(\diamond) \qquad \delta^J(\diamond) = R - P(\square \bigcirc \diamond)$$

$$\delta_{ij} = (1-\gamma) \cdot \delta_{ij}^M + \gamma \cdot \delta_{ij}^J, \quad 0 \le \gamma \le 1$$

$$\delta_{ij} = \begin{cases} \delta_{ij}^M, & if\ |\delta_j^M - \delta_j^J| \le \theta \\ \delta_{ij}^J, & if\ |\delta_j^M - \delta_j^J| > \theta \end{cases}$$

$$\delta_{ij} = \begin{cases} \delta_{ij}^M, & if\ sd(WOE) \le \theta \\ \delta_{ij}^J, & if\ sd(WOE) > \theta \end{cases}$$

Fig. S6. Computational modelling. (**a**) After getting feedback, the connection weight w_{ij} associated with each presented cue i and the chosen option j is updated by an error signal δ_{ij}, which represents a weighted sum of the marginal (δ_{ij}^M) and joint (δ_{ij}^J) updating prediction errors. (**b**) Marginal updating computes the prediction error for each cue as the difference between the observed outcome and the prediction made by that single cue alone, even though the outcome is

determined by all presented cues. By contrast, joint updating computes the error as the difference between the observed outcome and the prediction made by the integrated evidence of all cues. (**c**) Simulation results for marginal and joint updating after convergence (Methods). With single-cue training, both marginal and joint updating recover the objective WOE values. However, with multi-cue training, only joint updating recovers the objective WOE values and marginal updating produces biased estimates. Each thin line represents a simulated participant; thick lines and dots represent means across participants. Variation across thin lines reflects task stochasticity. (**d**) Empirical WOE values as a function of objective WOE values for single- and three-cue trials. Empirical WOE values are derived from the marginal reward probability of each cue (Methods). WOE values recovered by marginal updating match the empirical WOE values, suggesting that marginal updating learns marginal probabilities. (**e**) Hybrid learning models. We tested three models combining marginal and joint updating (Methods). In the hybrid constant model, the relative weight of marginal and joint updating is a free parameter, kept constant across all training trials. In the hybrid prediction error (PE) model, participants arbitrate between marginal and joint updating based on the distance between their prediction errors, switching to joint updating when the distance exceeds a threshold. The hybrid standard deviation (SD) model provides a heuristic approximation, using the SD of the learnt WOE values of the presented cues as the arbitration signal. (**f**) Model fitting results. Mean ΔAIC values (relative to the hybrid PE model) are shown separately for the singleton (N = 119) and parallel (N = 125) conditions. Smaller ΔAIC values indicate better fit to participants' behavior. Error bars indicate SEM. (**g**) The relationship between PE distance and the SD of WOE values in three-cue trials, derived from the hybrid PE model and pooled from both conditions. Each small dot represents the binned average for one participant with the bin determined by the SD of WOE values; large dark dots represent means across participants.

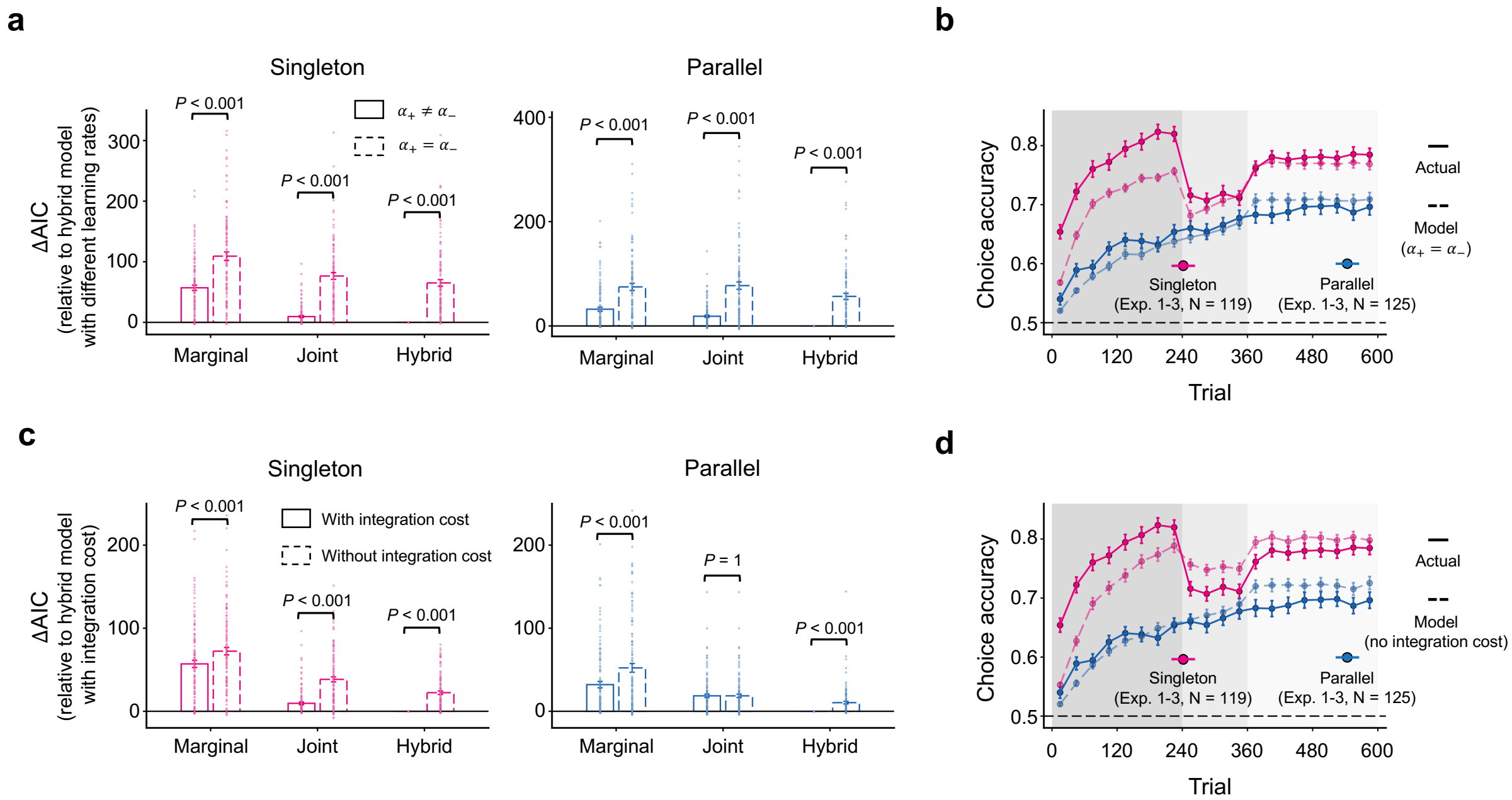

Fig. S7. Model estimation with constrained learning rates (a-b) and inverse temperatures (c-d). (**a**) To test whether participants applied distinct learning rates for positive and negative outcomes, we estimated models with a single shared learning rate and compared them to models with separate learning rates. In both singleton and parallel conditions, models with a shared learning rate yielded higher AIC values, indicating poorer fit. This supports the use of different learning rates for positive and negative outcomes. (**b**) Recovered versus observed choice accuracy across trials. Dark solid lines show pooled accuracy from Exp. 1–3; light dashed lines show predictions from the hybrid SD model with same learning rate for positive and negative outcomes. (**c**) To examine whether participants incurred an integration cost when moving from one-cue to multi-cue trials, we compared models that allowed separate inverse temperature parameters ($\beta_1 \neq \beta_3$) with those that constrained them to be equal ($\beta_1 = \beta_3$). Models without an integration cost fit worse than those allowing separate parameters, suggesting that integration costs are essential to capture participants' behavior. (**d**) Recovered versus observed choice accuracy across trials. Dark solid lines show pooled accuracy from Exp. 1–3; light dashed lines show predictions from the hybrid SD model without integration cost. In all figures, there are 119 and 125 participants in the singleton and parallel conditions, respectively. Error bars indicate SEM.

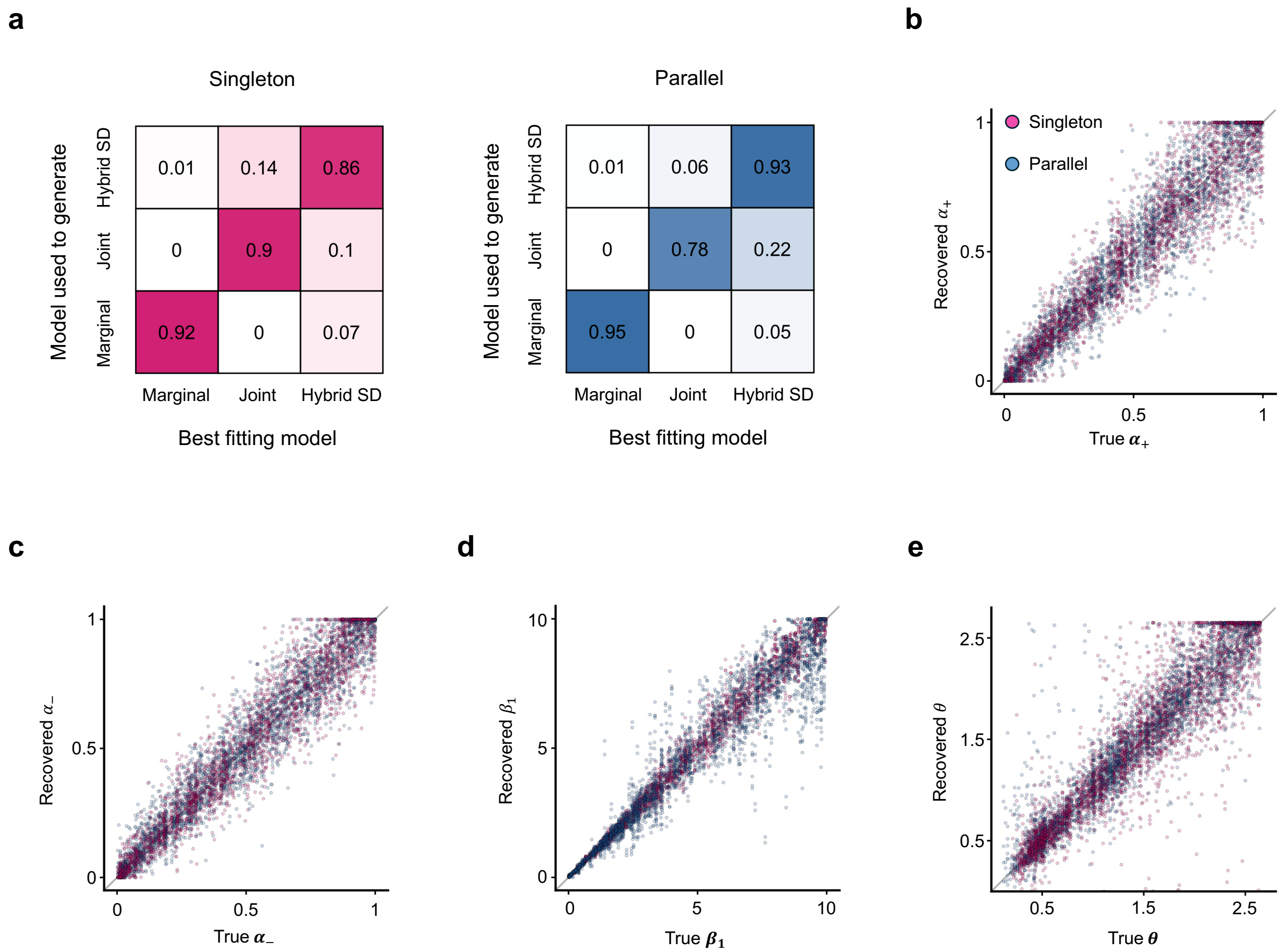

Fig. S8. Model and parameter recovery. (**a**) Model recovery. The model recovery focuses on the hybrid SD, pure marginal and pure joint updating models without relevant extensions (e.g., learning rate decay and attentional bias), since the central theoretical claim of this study is that participants rely on a hybrid learning mechanism rather than a single updating strategy. Specifically, we aimed to examine whether the hybrid updating model could be reliably distinguished from the single-strategy alternatives. Choices were first simulated under each model using parameter values randomly sampled from predefined distributions. The simulated data were then fitted with each candidate model, and the best-fitting model was identified based on the AIC value. For each participant, we ran five simulation-estimation loops with identical parameter values and repeated the full procedure five times (25 simulations total). Model recoverability was assessed by pooling data across all participants and computing the probability that each model produced the lowest AIC. Results are shown separately for singleton and parallel conditions. In both conditions, the model used to generate the simulated data was also most likely to be recovered, demonstrating that these models are distinguishable. The number in each cell represents the probability of each candidate model being the best model in capturing the behavioral choices simulated by one specific model of each row. To be noted, the sum of some rows is not identical to 1 because of rounding. (**b–e**) Parameter recovery results for hybrid SD model. We further tested whether the parameters of the hybrid SD model could be reliably estimated. Using the same procedure as for model recovery, we examined the correlation between true parameter values and

those recovered from model fitting. For all four parameters (α_+, α_-, β_1 and θ), the recovered values were strongly correlated with the true values, confirming reliable parameter identifiability. Each dot represents a data point from one participant; colors indicate experimental condition.

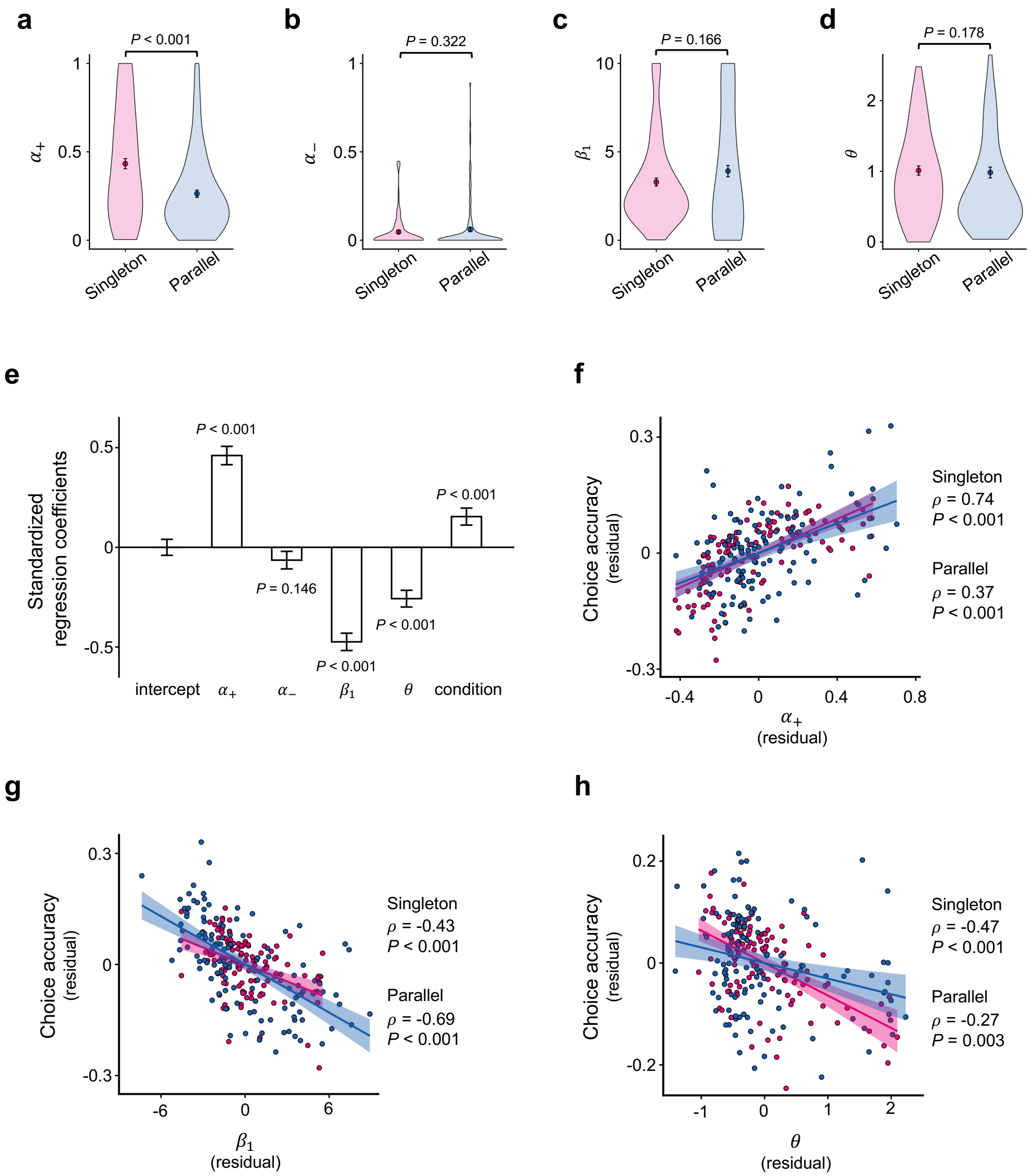

Fig. S9. Estimated parameters of the hybrid SD model. (**a–d**) Violin plots show the distributions of the four free parameters across participants, plotted separately for the singleton and parallel conditions. Dots and error bars indicate the mean and SEM across participants within each condition. Participants trained under the singleton condition exhibited a higher positive learning rate (α_+) compared to those in the parallel condition, while the other parameters (α_-, β_1 and θ) were comparable across conditions. (**e**) Regression analysis using estimated parameters and training condition to predict choice accuracy in test trials. The training condition was coded as 1 for singleton condition and 0 for parallel condition. Higher accuracy was associated with greater positive learning rate (α_+), lower integration noise (β_1), and lower cognitive cost for joint updating (θ). Critically, even after controlling for these parameter values, participants in the

singleton condition still outperformed those in the parallel condition, indicating that the learning advantage of singleton training is not solely explained by parameter differences but by the specific design of the training curricula. (**f–h**) Relationships between choice accuracy and individual parameters (α_+, β_1 and θ), shown for illustration of the regression results in panel **e**. Each dot represents an individual participant. The shaded areas around each fitted line indicate 95% confidence interval (CI). In all figures, there are 119 and 125 participants in the singleton and parallel conditions, respectively. Error bars indicate SEM.

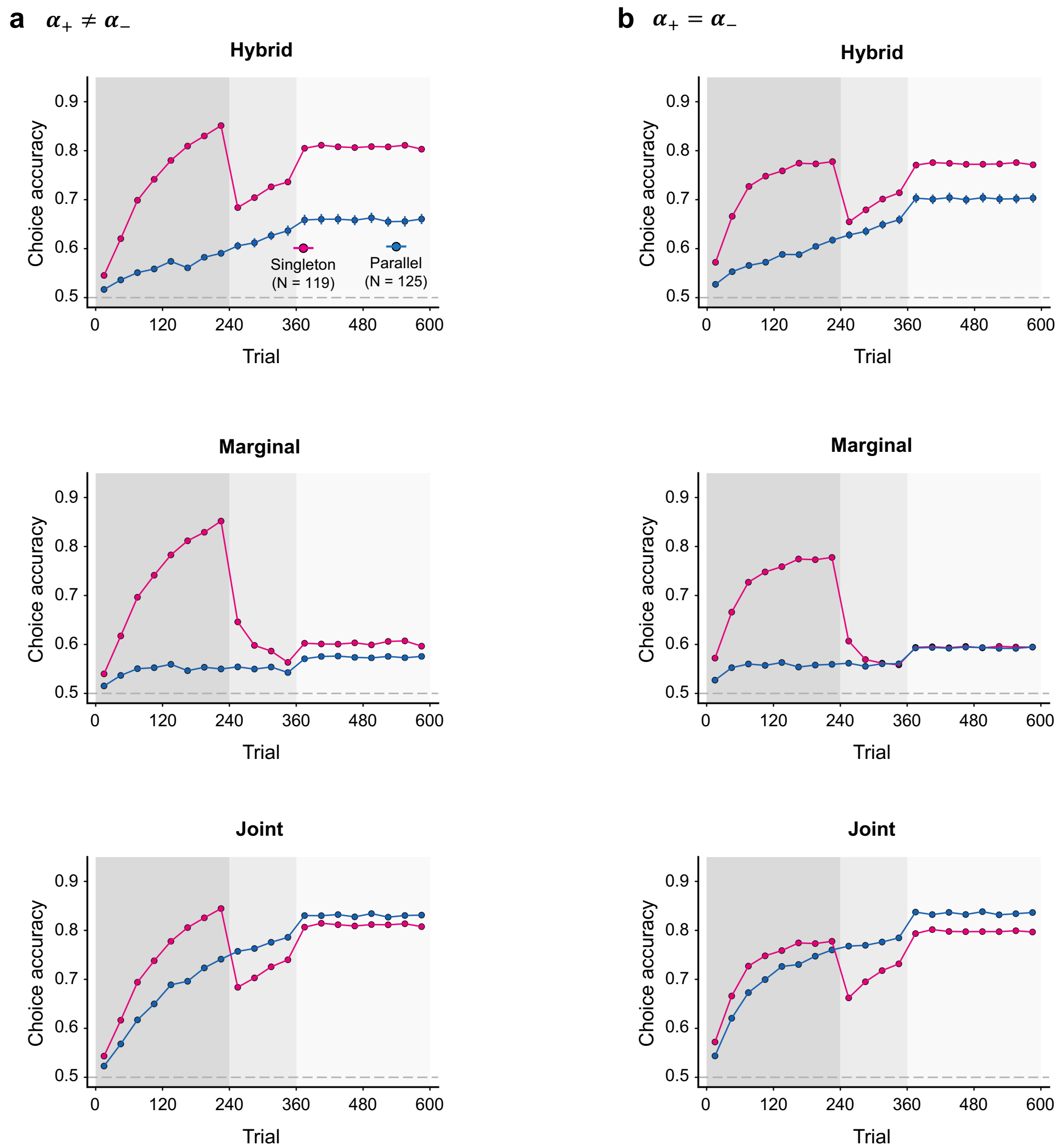

Fig. S10. Model simulations with identical parameter values across conditions. (**a**) To test whether the singleton advantage arises from parameter differences between conditions, we simulated participants' choices using identical parameter values for both singleton and parallel training. Specifically, we used the median of the parameter estimates derived from the hybrid SD model in the parallel condition for simulations. Even with matched parameters, the hybrid SD model robustly reproduced the singleton learning advantage (top panel). Notably, this effect was unique to the hybrid model: when simulations were run with either the marginal or joint updating model under the same parameters, the singleton advantage disappeared (middle and bottom panels). (**b**) To further rule out differences between positive and negative learning rates as a potential explanation, we repeated the simulations after equating the two learning rates. Specifically, we replaced the value of negative learning rate with that of the positive learning rate. Results mirrored those shown in **a**, confirming that the singleton effect cannot be solely attributed to asymmetries in learning rates. Dots represent the mean choice accuracy across participants, calculated over 30 consecutive trials.

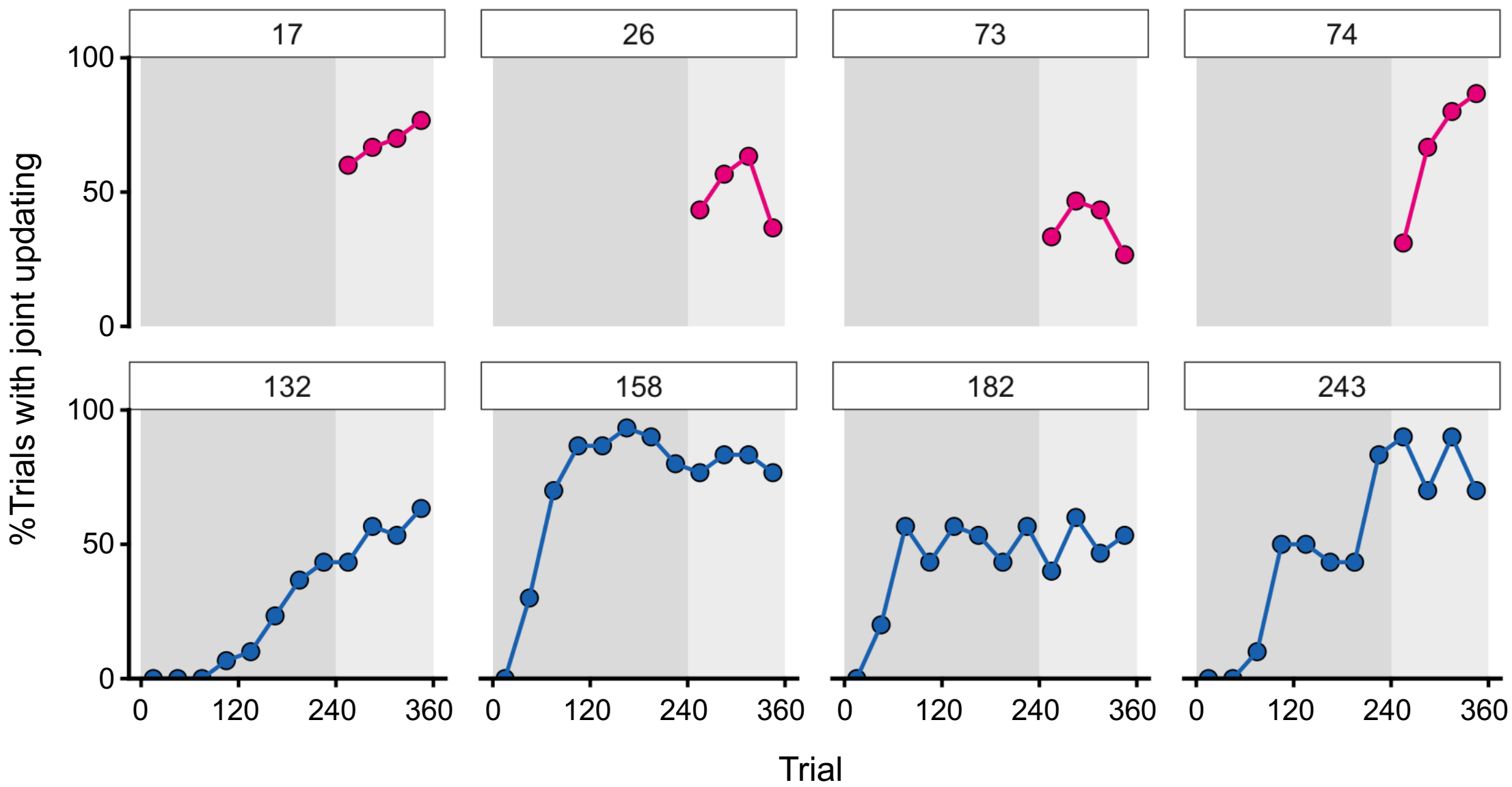

Fig. S11. The dynamics of learning strategies in example participants. Each panel illustrates how learning strategies evolve for one participant. The top row shows four example participants from the singleton condition, and the bottom row shows four example participants from the parallel condition. As the marginal and joint updating cannot be separable in one-cue trials, we only present the results from multi-cue trials. Each dot represents the average proportion of multi-cue trials (calculated over a moving window of 30 trials) in which the participant used the joint updating strategy. The number above each panel indicates the participant's ID.

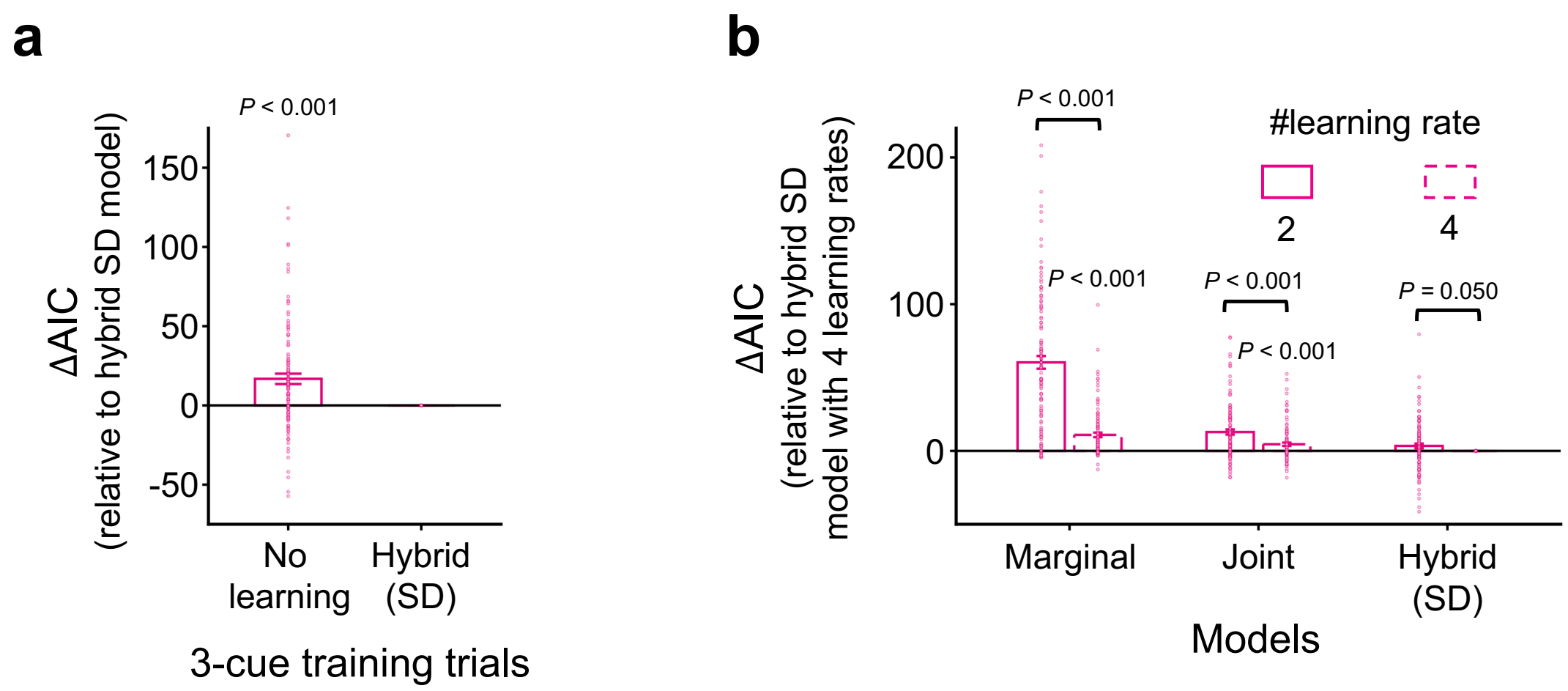

Fig. S12. Alternative models in the singleton condition (N = 119). (**a**) Mean ΔAIC values for models with and without learning during three-cue training trials. To test whether participants stopped learning once exposed to three-cue trials following one-cue training, we compared models that allowed versus prevented learning in three-cue trials. The hybrid learning model provided a better fit than the model without learning, suggesting that participants continued learning during three-cue trials. (**b**) Mean ΔAIC values for models with either shared or distinct learning rates for one-cue and three-cue training trials. To test whether participants used different learning rates depending on cue number, we compared models with four learning rates against models with two. Allowing distinct learning rates improved the model fit for marginal and joint updating models, but not for the hybrid SD model. For simplicity, we used the same learning rate for trials with different cue numbers, while noting that allowing different learning rates may improve estimation. Importantly, across all specifications, the hybrid model consistently outperformed the single-strategy models. In all figures, error bars denote SEM.

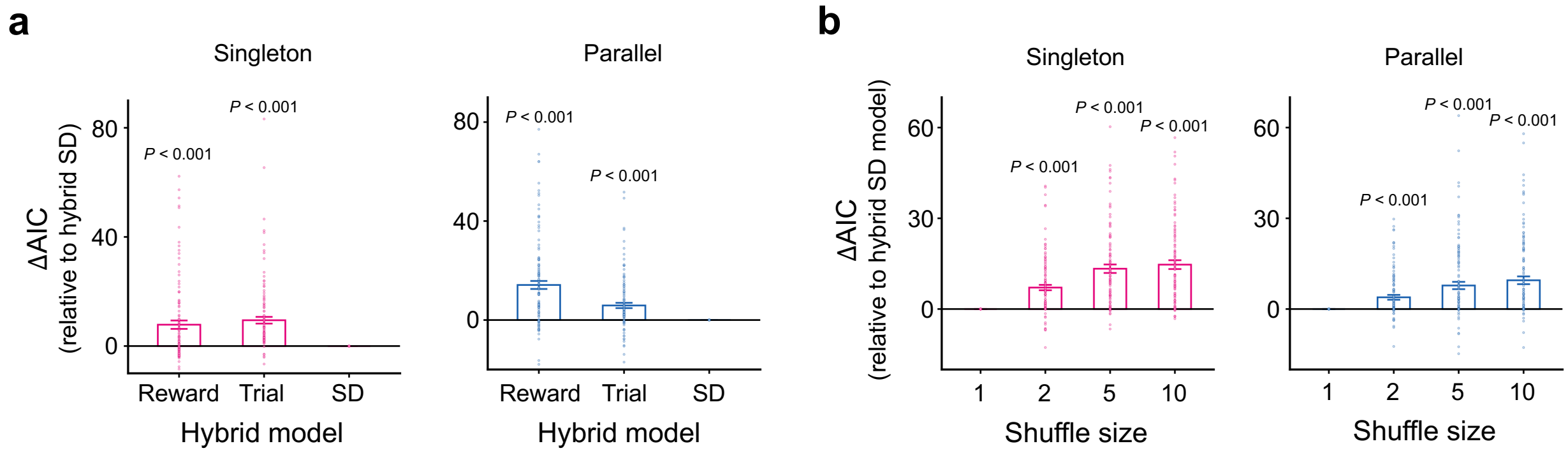

Fig. S13. Alternative explanations for the hybrid learning mechanism. (**a**) Mean ΔAIC value for models that determine the weight of marginal versus joint updating in different ways. The hybrid SD model showed that participants increased the weight of joint updating over the course of training. One possibility for this learning dynamic is that participants simply increased the weight of joint updating as a function of trial number. That is, $\gamma = \frac{1}{1+e^{-b_1 \cdot t + b_0}}$, where t is the trial number, b_1 and b_0 are two free parameters. Another is that the updating strategy was determined by whether the current trial was rewarded (i.e., joint updating in rewarded trials and marginal updating in unrewarded trials). Both these two possibilities could yield an increasing proportion of joint updating trials over training. To exclude these possibilities, we compared the model estimation results of these models with the hybrid SD models. The results showed that neither of these accounts outperformed the hybrid SD model. (**b**) Mean ΔAIC value for models with a shuffled mapping between the updating strategies and the specific trials. We also considered whether only the overall proportion of joint updating trials mattered, rather than the specific mapping of different strategies to trials. To test this, we preserved the trial-by-trial proportion of joint updating inferred from the hybrid SD model but randomly shuffled the assignment of marginal and joint updating strategy to trials within small temporal windows (sizes = 1, 2, 5, or 10 trials; with size = 1 corresponding to the original model). After each shuffling, we estimated the other parameters in the model (i.e., α_+, α_-, and β_1). If only the overall proportion mattered, shuffling should not reduce model fit. Instead, even minimal shuffling (window size = 2) impaired model performance, indicating that the trial-specific assignment of strategies is critical. These results suggest that participants adaptively chose updating strategies on a trial-by-trial basis, as captured by the hybrid SD model. In all figures, there are 119 and 125 participants in the singleton and parallel conditions, respectively. Error bars indicate SEM.

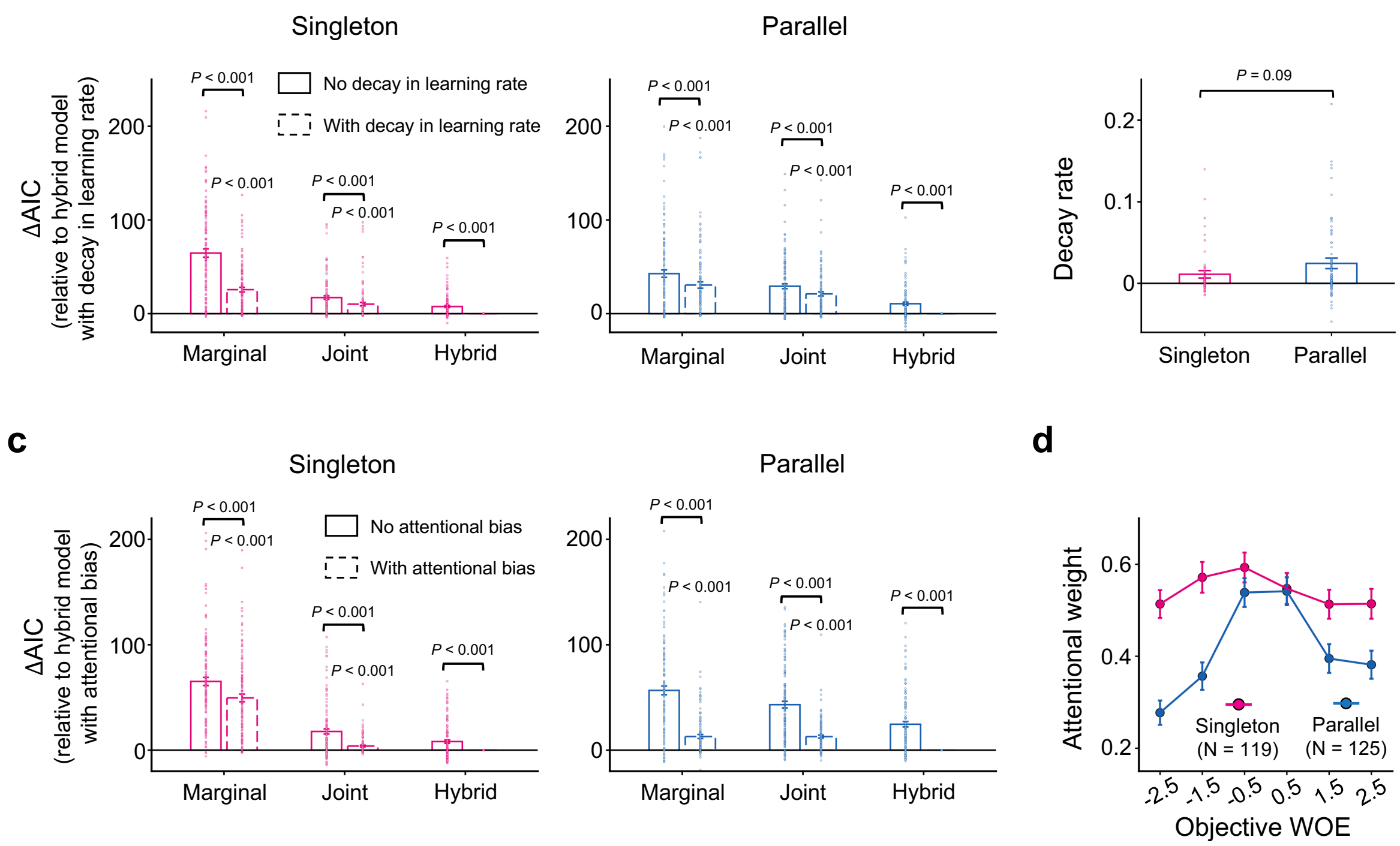

Fig. S14. Extension of the hybrid learning framework. (**a**) Mean ΔAIC values for models with and without decay in learning rates. To test whether the observed behavioral patterns could be explained by changes in learning rate over time, rather than by a hybrid updating mechanism, we added a decay parameter that allowed the learning rates to decrease across trials (Methods). Incorporating decay improved the fit of all models in both conditions, suggesting that participants reduced their learning speed as training progressed, possibly due to cognitive fatigue. However, the hybrid SD model continued to outperform both the marginal-only and joint-only models, demonstrating that a mixed strategy provides the best account of behavior. (**b**) Mean values of the decay parameter. We found no reliable evidence that the decay parameter differed between conditions using either parametric or non-parametric tests (independent-samples t-test: $t(221.2) = -1.7$, $P = 0.09$, Cohen's $d = -0.22$ (-0.47, 0.03); Wilcoxon rank sum test: W = 6428, $P = 0.07$), consistent with comparable learning motivation across conditions. (**c**) Mean ΔAIC values for models with and without attentional biases. Previous studies have shown that learners assign different attentional weights to different features in multi-cue tasks[1–3]. To test whether this applied to our cue combination task, we extended each model by adding an attentional weight parameter for each cue, applied during both learning and decision stages (Methods). Including attentional weights improved model fits, but the hybrid SD model remained the best-fitting account overall. (**d**) The estimated value of the attentional weight for each cue. The estimated attentional weights showed an inverted U-shaped relationship with the objective WOE values: cues with more extreme WOE values were assigned less weight. This pattern is consistent with the 'robust averaging' effect[4,5], where learners down-weight extreme information to avoid being misled. In all figures, there are 119 and 125 participants in the singleton and parallel conditions, respectively. Error bars indicate SEM.

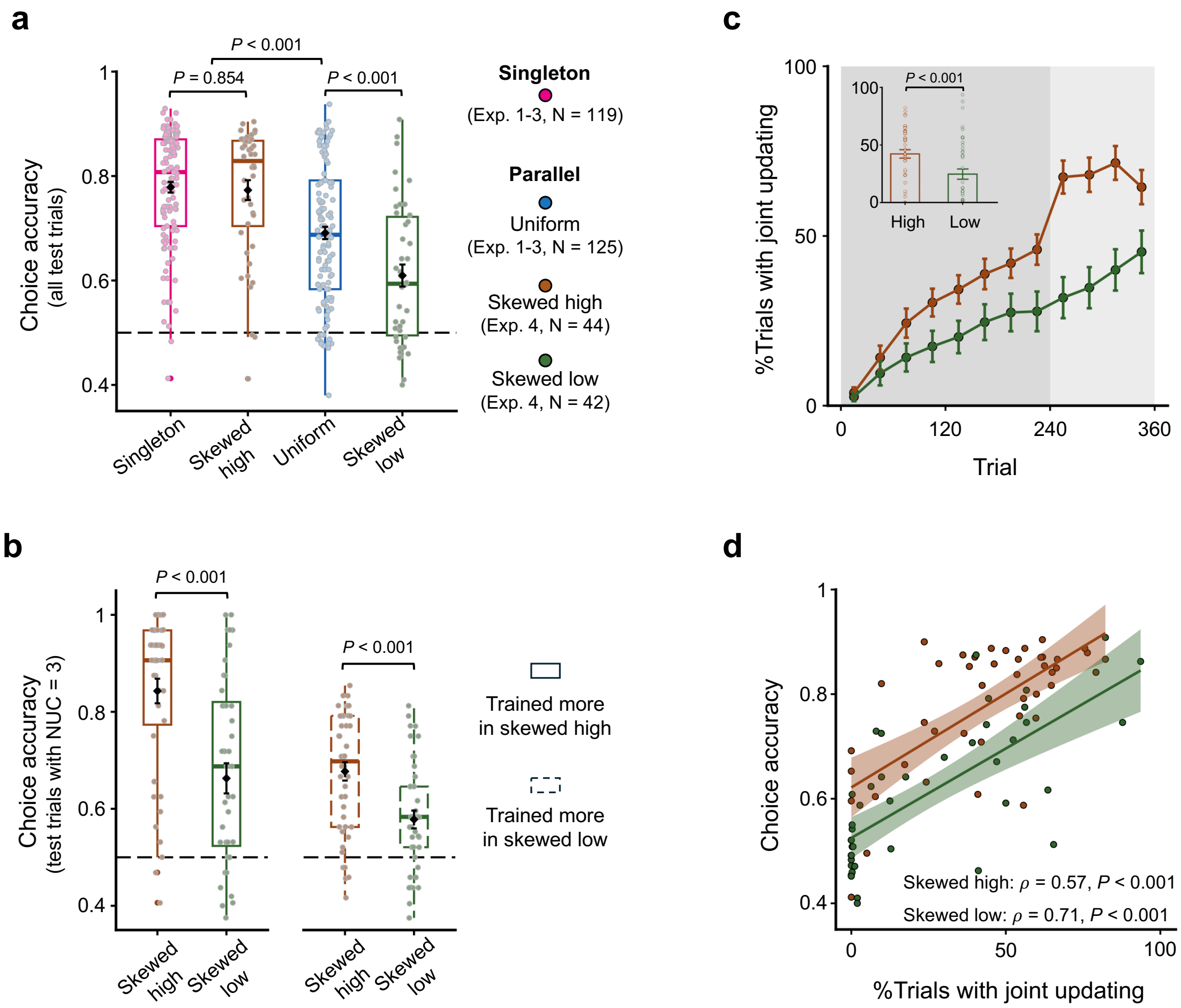

Fig. S15. Additional results for Exp. 4. (**a**) Boxplots display the mean choice accuracy averaged across all test trials separately for different conditions. Each colored dot represents an individual participant. Each black dot represents the mean across participants within a condition, and the error bars surrounding indicate SEM. (**b**) Mean choice accuracy averaged across test trials with three unique cues (NUC = 3) for the skewed high and skewed low conditions, shown separately by whether a specific cue combination was more frequently trained in the skewed high or skewed low condition. Error bars denote SEM. (**c**) Proportion of trials with joint updating, as recovered by the hybrid SD model, plotted over the course of training. Each dot represents the mean across participants, calculated over 30 consecutive trials. The inset shows the mean across all training trials. (**d**) Relationship between learning performance and the proportion of joint updating. The shaded areas around each fitted line indicate 95% CI. Each dot represents an individual participant.

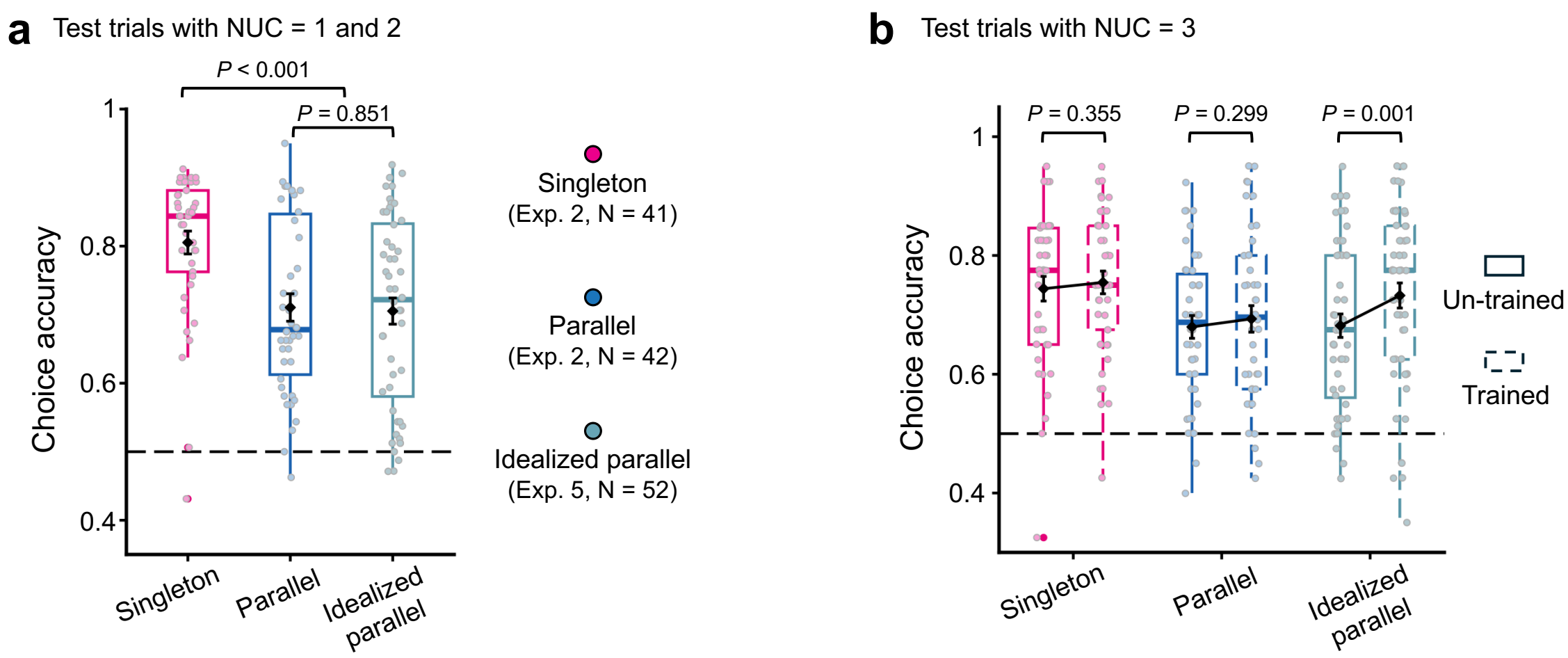

Fig. S16. Additional results for Exp. 5. (**a**) Mean choice accuracy averaged across test trials with NUC = 1 and 2. (**b**) Mean choice accuracy averaged across test trials with NUC = 3, separated by whether a specific combination was included in the training trials. Error bars indicate SEM.

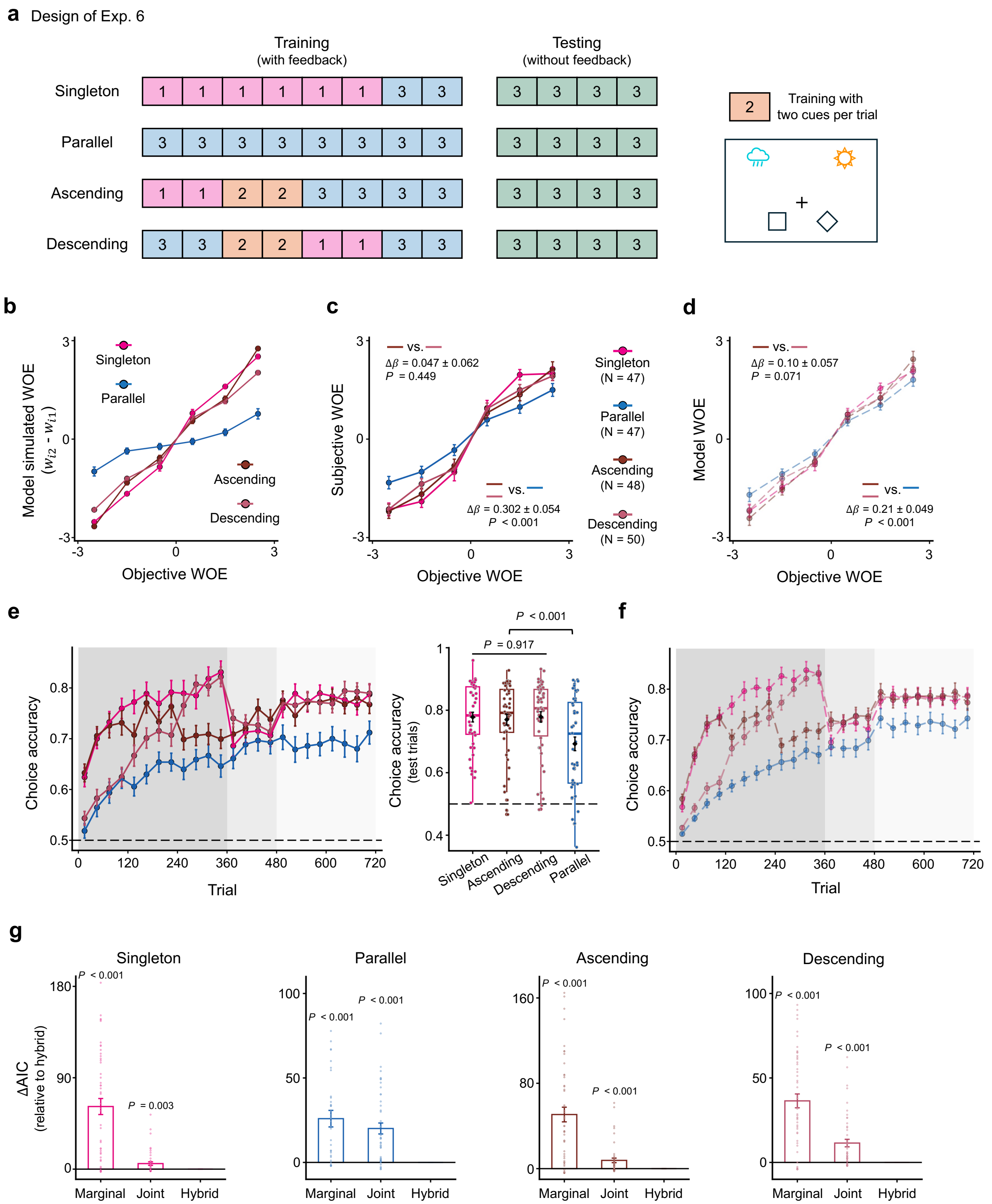

Fig. S17. Comparable learning performance under ascending and descending training (Exp. 6). (**a**) Experimental design. Each square represents a block of 60 trials, with numbers indicating the number of cues per trial. Colors denote trial types: pink = single cue, brown = two unique cues, blue = three unique cues, and green = three cues that may not be unique (Methods). (**b**) Out-of-

sample model-predicted WOE values for each cue, simulated from the hybrid SD model and plotted against the objective WOE value. Parameter values used in these simulations were derived from Exp. 1-3. (**c**) Subjective WOE values recovered by logistic regressions based on participants' choices in test trials. (**d**) Model-estimated WOE values recovered from the hybrid SD model, fitted to participants' actual choices. (**e**) Left: Mean choice accuracy averaged across trials and participants, derived from participants' actual choices and plotted as a function of trials. The light grey, grey and dark grey shaded areas indicate different task stages (360, 120 and 240 trials, respectively). Right: Mean choice accuracy averaged across all test trials for each condition separately. (**f**) Mean choice accuracy predicted by the hybrid SD model, averaged across trials and participants, is plotted over trials. (**g**) Mean ΔAIC value of each model (relative to the hybrid SD model), averaged across participants and shown separately for each condition. Each dot represents an individual participant. In all figures, there are 47, 47, 48 and 50 participants in the singleton, parallel, ascending and descending conditions, respectively. Error bars indicate SEM.

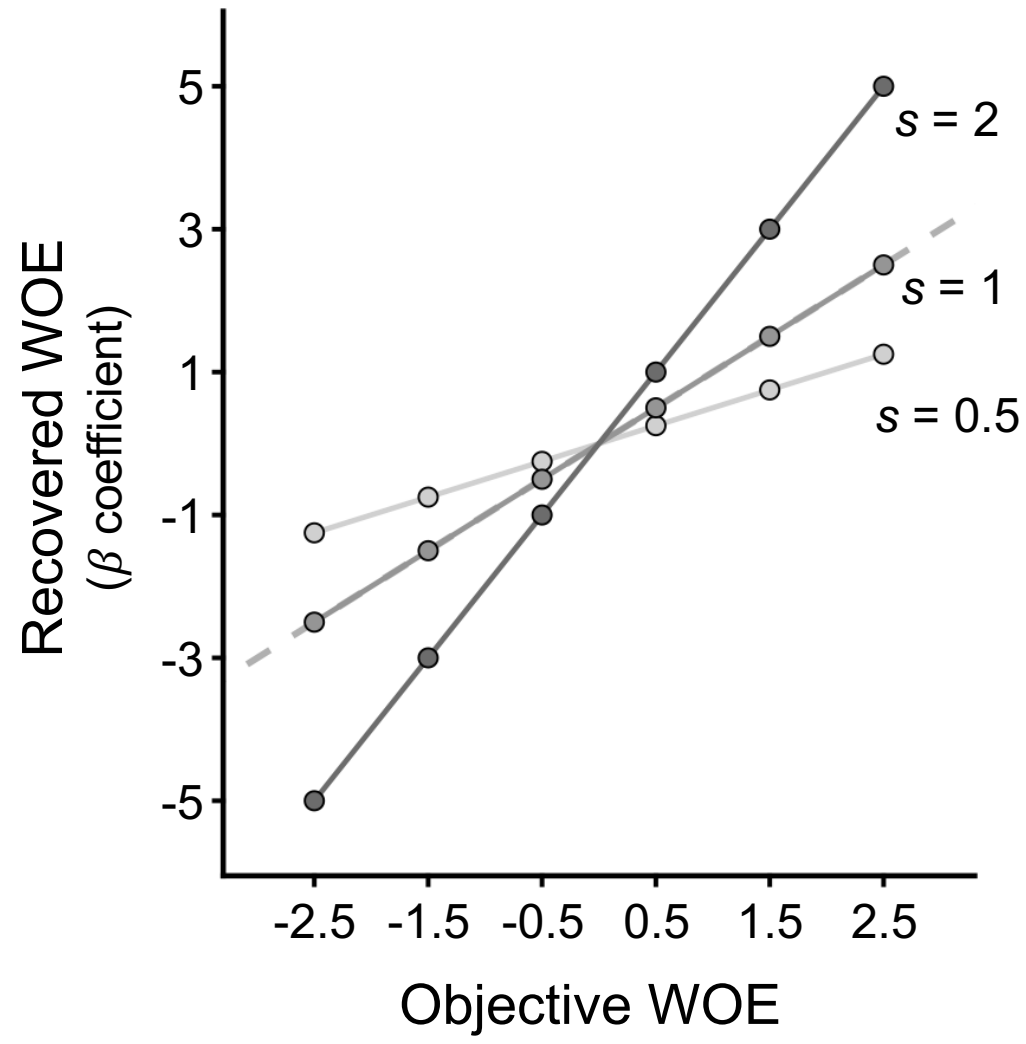

Fig. S18. Recovered WOE values as a function of objective WOE under different scaling factors. We simulated participants' choices in test trials using objective WOE values assigned to each cue, adjusted by a scaling factor (*s*). For instance, when $s = 1$, the WOEs used to generate behavior match the objective WOEs, whereas when $s = 0.5$, the WOEs are scaled to half of the objective values. Logistic regressions (Methods) were used to recover the WOEs for each cue based on simulated choices. The recovered WOE values matched the WOEs used to generate the data. The dashed grey line represents the diagonal line.

Table S1. Summary of the modelling results for Exp. 1–3. Estimated values of free parameters are presented as mean (SEM) in each entry. α_+, learning rate for positive outcome; α_-, learning rate for negative outcome; β_1, integration cost; γ, weight of joint updating; θ, threshold for determining joint versus marginal updating; Hybrid PE, hybrid learning models with the learning strategy in each trial determined by the distance in prediction errors generated by marginal and joint updating; Hybrid SD, hybrid learning models with the learning strategy in each trial determined by the standard deviation of the learnt WOE values of the presented cues; -LL, negative log likelihood; AIC, Akaike information criterion. Note that the inverse temperature for three-cue trials was set to 1 for all models. There are 119 and 125 participants in the singleton and parallel conditions, respectively.

Condition	Models	α_+	α_-	β_1	γ	θ	-LL	AIC
Singleton	Marginal	0.37 (0.023)	0.06 (0.012)	1.7 (0.18)			276.5 (6.05)	559.0 (12.11)
	Joint	0.36 (0.028)	0.03 (0.008)	4.5 (0.27)			252.8 (7.76)	511.52 (15.53)
	Hybrid constant	0.43 (0.029)	0.05 (0.010)	3.7 (0.25)	0.80 (0.030)		250.8 (7.69)	509.6 (15.38)
	Hybrid PE	0.45 (0.029)	0.05 (0.009)	3.3 (0.21)		0.30 (0.013)	246.6 (7.74)	501.3 (15.47)
	Hybrid SD	0.43 (0.029)	0.05 (0.009)	3.3 (0.23)		0.97 (0.056)	247.0 (7.72)	502.0 (15.45)
Parallel	Marginal	0.18 (0.015)	0.07 (0.012)	2.3 (0.29)			327.1 (5.81)	660.1 (11.62)
	Joint	0.17 (0.019)	0.01 (0.005)				321.3 (7.08)	646.6 (14.17)
	Hybrid constant	0.29 (0.022)	0.06 (0.013)	4.8 (0.38)	0.55 (0.032)		313.2 (6.98)	634.3 (14.00)
	Hybrid PE	0.32 (0.023)	0.08 (0.014)	4.1 (0.33)		0.35 (0.017)	308.3 (7.20)	624.6 (14.41)
	Hybrid SD	0.25 (0.020)	0.06 (0.013)	3.8 (0.32)		0.86 (0.062)	310.0 (7.19)	628.0 (14.37)

References

1. Akaishi, R., Kolling, N., Brown, J. W. & Rushworth, M. Neural Mechanisms of Credit Assignment in a Multicue Environment. *J. Neurosci.* **36**, 1096–1112 (2016).
2. Leong, Y. C., Radulescu, A., Daniel, R., DeWoskin, V. & Niv, Y. Dynamic Interaction between Reinforcement Learning and Attention in Multidimensional Environments. *Neuron* **93**, 451–463 (2017).
3. Wang, M. C. & Soltani, A. Contributions of Attention to Learning in Multidimensional Reward Environments. *J. Neurosci.* **45**, e2300232024 (2025).
4. Li, V., Herce Castañón, S., Solomon, J. A., Vandormael, H. & Summerfield, C. Robust averaging protects decisions from noise in neural computations. *PLOS Comput. Biol.* **13**, e1005723 (2017).
5. De Gardelle, V. & Summerfield, C. Robust averaging during perceptual judgment. *Proc. Natl. Acad. Sci.* **108**, 13341–13346 (2011).
