# Supplementary material for: Human curriculum learning of a cue combination task
Source: Nat Hum Behav. 2026 May 5;10(8):1539–56. doi: 10.1038/s41562-026-02452-1 (PMC13485681; doi:10.1038/s41562-026-02452-1)
Supplement: Supplementary file 2 [file 41562_2026_2452_MOESM2_ESM.pdf]

nature portfolio

Corresponding author(s): Qingtian Mi, Christopher Summerfield

Last updated by author(s): 2026-3-13

Reporting Summary

Nature Portfolio wishes to improve the reproducibility of the work that we publish. This form provides structure for consistency and transparency in reporting. For further information on Nature Portfolio policies, see our Editorial Policies and the Editorial Policy Checklist.

Statistics

For all statistical analyses, confirm that the following items are present in the figure legend, table legend, main text, or Methods section.

n/a	Confirmed	
☐	☒	The exact sample size (*n*) for each experimental group/condition, given as a discrete number and unit of measurement
☐	☒	A statement on whether measurements were taken from distinct samples or whether the same sample was measured repeatedly
☐	☒	The statistical test(s) used AND whether they are one- or two-sided<br>*Only common tests should be described solely by name; describe more complex techniques in the Methods section.*
☐	☒	A description of all covariates tested
☐	☒	A description of any assumptions or corrections, such as tests of normality and adjustment for multiple comparisons
☐	☒	A full description of the statistical parameters including central tendency (e.g. means) or other basic estimates (e.g. regression coefficient) AND variation (e.g. standard deviation) or associated estimates of uncertainty (e.g. confidence intervals)
☐	☒	For null hypothesis testing, the test statistic (e.g. *F*, *t*, *r*) with confidence intervals, effect sizes, degrees of freedom and *P* value noted<br>*Give P values as exact values whenever suitable.*
☒	☐	For Bayesian analysis, information on the choice of priors and Markov chain Monte Carlo settings
☐	☒	For hierarchical and complex designs, identification of the appropriate level for tests and full reporting of outcomes
☐	☒	Estimates of effect sizes (e.g. Cohen's *d*, Pearson's *r*), indicating how they were calculated

Our web collection on statistics for biologists contains articles on many of the points above.

Software and code

Policy information about availability of computer code

Data collection	Data was collected from the well-established online research platform Prolific (https://www.prolific.com), using custom scripts based on jsPsych (v7.0.0; https://www.jspsych.org/v7/).
Data analysis	Data was analyzed using custom scripts based on MATLAB (2024; https://mathworks.com) and R (v4.3.3; https://www.r-project.org). Modelling code is available on Open Science Framework (https://osf.io/gxv4z/).

For manuscripts utilizing custom algorithms or software that are central to the research but not yet described in published literature, software must be made available to editors and reviewers. We strongly encourage code deposition in a community repository (e.g. GitHub). See the Nature Portfolio guidelines for submitting code & software for further information.

Data

Policy information about availability of data

All manuscripts must include a data availability statement. This statement should provide the following information, where applicable:

- Accession codes, unique identifiers, or web links for publicly available datasets
- A description of any restrictions on data availability
- For clinical datasets or third party data, please ensure that the statement adheres to our policy

Human data are made freely available on Open Science Framework (https://osf.io/gxv4z/) .

Research involving human participants, their data, or biological material

Policy information about studies with human participants or human data. See also policy information about sex, gender (identity/presentation), and sexual orientation and race, ethnicity and racism.

Reporting on sex and gender	Participants' gender information (male, female, or other) was collected via self-reporting. Of the 667 participants included in the final data analyses, 347 identified as female, 312 as male, and 8 as other. We did not include sex or gender as variables in our analyses, given we had no hypotheses about why any individual characteristic, including sex or gender, would affect our hypotheses. Indeed we feel our results indicate basic learning mechanisms regardless of individual variability.
Reporting on race, ethnicity, or other socially relevant groupings	We did not collect and include any socially constructed or socially relevant categorization variables, given we had no hypotheses about why any individual characteristic, including race or ethnicity, would affect our hypotheses.
Population characteristics	See above. See also details for each experiment below.
Recruitment	Participants were recruited from the well-established online research platform Prolific. Eligibility criteria included the following: age between 18 and 30 years for Exp. 1-6 and age between 18 and 60 years for pointer game, a Prolific approval rate above 90%, no color blindness, no history of neurological or psychiatric illness, and no prior participation in the previous versions of this study. Eligible participants were randomly assigned to different experimental conditions.
Ethics oversight	Ethical approval was obtained from the University of Oxford Central University Research Ethics Committee.

Note that full information on the approval of the study protocol must also be provided in the manuscript.

Field-specific reporting

Please select the one below that is the best fit for your research. If you are not sure, read the appropriate sections before making your selection.

☐ Life sciences ☒ Behavioural & social sciences ☐ Ecological, evolutionary & environmental sciences

For a reference copy of the document with all sections, see nature.com/documents/nr-reporting-summary-flat.pdf

Behavioural & social sciences study design

All studies must disclose on these points even when the disclosure is negative.

Study description	The study involved a quantitative experimental design.
Research sample	The research samples for all experiments consisted of participants from Prolific. Here are the demographic information for the participants in each experiment:<br>Experiment 1: 58 females, 50 males and 1 other; age = 25.2 ± 3.37 ys (Mean ± S.D.);<br>Experiment 2: 25 females, 26 males and 1 other; age = 25.1 ± 3.60 ys;<br>Experiment 3: 48 females, 34 males and 1 other; age = 24.2 ± 3.93 ys;<br>Experiment 4: 41 females, 44 males and 1 other; age = 24.8 ± 3.39 ys;<br>Experiment 5: 33 females, 19 males and 0 other; age = 24.8 ± 3.61 ys ;<br>Experiment 6: 93 females, 95 males and 4 others; age = 24.6 ± 3.76 ys;<br>Pointer game: 49 females, 44 males and 0 other; age = 31.9 ± 9.45 ys.<br>These samples were representative of the overall US and UK population.
Sampling strategy	We utilized a convenience sampling strategy, allowing participation for individuals who accepted the invitation. To determine the initial sample size for Experiment 1, we conducted a pilot study with 67 participants (N = 36 in the singleton condition and N = 31 in the parallel condition) using a similar experimental design but with fewer trials than the main experiment. Based on the pilot results, we increased the number of trials and qualitatively estimated the required sample size to achieve statistical significance before collecting the final dataset. The final sample size in the current study is comparable to those reported in previous studies (e.g.,)
Data collection	Participants performed the task computerized over the internet. Experimenters were not present during data collection and did not influence or interact with participants during the experiment.
Timing	The data was collected between May 19, 2024 and November 12, 2025.
Data exclusions	Participants were excluded from data analysis according to the same criteria for all experiments. Specifically, exclusion occurred if a participant met one or more of the following conditions: (i) providing the same response for more than 30 consecutive trials (half the total trials in a block), (ii) an average button press count exceeding 1.5 per trial in a block, (iii) reaction times below 250 ms for more than 12 trials in a block (20% of the total trials in a block), (iv) low accuracy on sanity check trials, or (v) reporting issues with stimulus display. In total, 114 participants were excluded from the final data analyses, resulting in an exclusion rate of 16.6%, which is consistent with previous online studies (e.g., Hardy et al., 2023, Nature Human Behaviour; Hayes & Wedell, 2023, Psychonomic Bulletin & Review; Garcia et al., 2023, Nature Human Behaviour; Findling & Wyart, 2024, Science Advances).

Non-participation	No participants dropped out or declined participation.
Randomization	Participants were randomly assigned to different conditions in each experiment.

Reporting for specific materials, systems and methods

We require information from authors about some types of materials, experimental systems and methods used in many studies. Here, indicate whether each material, system or method listed is relevant to your study. If you are not sure if a list item applies to your research, read the appropriate section before selecting a response.

Materials & experimental systems

n/a	Involved in the study
☒	☐ Antibodies
☒	☐ Eukaryotic cell lines
☒	☐ Palaeontology and archaeology
☒	☐ Animals and other organisms
☒	☐ Clinical data
☒	☐ Dual use research of concern
☒	☐ Plants

Methods

n/a	Involved in the study
☒	☐ ChIP-seq
☒	☐ Flow cytometry
☒	☐ MRI-based neuroimaging

Plants

Seed stocks	*Report on the source of all seed stocks or other plant material used. If applicable, state the seed stock centre and catalogue number. If plant specimens were collected from the field, describe the collection location, date and sampling procedures.*
Novel plant genotypes	*Describe the methods by which all novel plant genotypes were produced. This includes those generated by transgenic approaches, gene editing, chemical/radiation-based mutagenesis and hybridization. For transgenic lines, describe the transformation method, the number of independent lines analyzed and the generation upon which experiments were performed. For gene-edited lines, describe the editor used, the endogenous sequence targeted for editing, the targeting guide RNA sequence (if applicable) and how the editor was applied.*
Authentication	*Describe any authentication procedures for each seed stock used or novel genotype generated. Describe any experiments used to assess the effect of a mutation and, where applicable, how potential secondary effects (e.g. second site T-DNA insertions, mosiacism, off-target gene editing) were examined.*
