# Supplementary material for: Human curriculum learning of a cue combination task
Source: Nat Hum Behav. 2026 May 5;10(8):1539–56. doi: 10.1038/s41562-026-02452-1 (PMC13485681; doi:10.1038/s41562-026-02452-1)
Supplement: Supplementary file 3 [file 41562_2026_2452_MOESM3_ESM.pdf]

nature portfolio

Peer Review File

Human curriculum learning of a cue combination task

Corresponding Author: Dr Qingtian Mi

Version 0:

Decision Letter:

30th April 2025

Dear Dr Summerfield,

Thank you once again for your manuscript, entitled "Human curriculum learning of a cue combination task," and for your patience during the peer review process.

Your manuscript has now been evaluated by 2 reviewers, whose comments are included at the end of this letter. Please note that we had initially invited an additional reviewer but they failed to return their comments. In the light of reviewer feedback, I regret that we cannot offer to publish your manuscript in Nature Human Behaviour.

While the reviewers find your work of some interest, they raise concerns about the strength of the novel conclusions that can be drawn at this stage. We feel that these reservations are sufficiently important as to preclude publication of this work in Nature Human Behaviour.

I am sorry that we cannot be more positive on this occasion but hope that you will find our reviewers' comments helpful when preparing your paper for submission elsewhere.

Sincerely,

[redacted]
Nature Human Behaviour

Reviewer expertise:

Reviewer #1: Decision making, credit assignment, computational models

Reviewer #3: Curriculum learning, cognitive control, computational neuroscience

Reviewers' Comments:

Reviewer #1 (Remarks to the Author):

In this article, Mi and Summerfield investigate the role of curriculum learning in a version of the weather prediction task, a multi-cue binary probabilistic learning task. They first present 3 experiments establishing the finding that “curriculum learning” (breaking down the problem into parts) helps learning. They then propose a “bounded rationality” neural network framework to show qualitative similar behavior. Finally, they use this framework to design novel curricula which they predict should enhance or impair learning, and test this in two final experiment.

This paper is well written, investigates a question of broad interest, and uses carefully designed behavioral experiments to test it, with rigorous methods. However, major issues limit the value of this contribution, including issues with a) ruling out alternative hypotheses; b) testing competing mechanisms; and c) supporting the proposed theoretical interpretation. Furthermore, the generalizability of the findings is very limited.

Major issues

1. The most important issue is in the modeling framework. Model methods state that the network is trained for 100 epochs (each corresponding to the full empirical experiment) to make predictions; predictions come from the asymptotic performance phase (top of p27). To rephrase, it means that the network is exposed to 2 orders of magnitude more trials than the participants, and not in the same order, despite the whole point of the paper being to investigate the role that the learning regime impacts learning. This approach seems very misleading, as most would read this paper and interpret it as saying that the authors offer an explanation of how learning dynamic unfolds. Instead, they propose a fixed snapshot of what might be known at the end of learning, with no validly interpretable explanation of how the human participants might arrive there. To be interpretable, the framework should lead to interpretable predictions over the same training as participants, and capture learning trajectories as well as testing phase.

2. A secondary major issue comes from the lack of competing models, besides the optimal one. This type of task has been extensively studied and modeled, and approaches like attention-modulated RL models (e.g. Niv and colleagues) or other algorithmic approaches might offer alternative explanations; these explanations are not explored or discussed.

3. The controls performed in experiments 2 and 3 are of limited value. For experiment 2, it is perfectly possible that motivation might be reset by being offered a novel opportunity with new stimuli to learn about – it is well known that someone might give up on a “too hard” context, but try again in a new context. L34 p4 is not a satisfactory interpretation. For experiment 3, half of the total combination of cues is still 10 combinations, which is still way above working memory capacity/resource limitations, so is again not a good control for the proposed rationale of ruling out memory effects.

4. The results of the last experiment (fig. S4) are described as matching the predictions of the model, but this is not fully the case: ascending and descending do not differ from singleton, whereas the model predicts a difference. The other prediction of the model is that there should be no difference between the ascending and descending conditions, and this prediction is borne, but this is a null effect in a highly variable sample, and no test of this null hypothesis is given.

5. The exclusion rate is not reported (only final number of participants included, and exclusion criteria; missing number of participants tested).

6. It is unclear whether the broader conclusion about specific curricula, or the use of the theoretical framework, has any chance of translating to other learning situations, in particular in light of the lack of validity of the theoretical framework as a learning mechanism.

No minor issues. Thanks for a well edited paper.

Reviewer #3 (Remarks to the Author):

Summary

The study investigates how different training curricula affect human learning in a probabilistic cue combination task. Across six behavioral experiments, participants learn to predict weather outcomes based on up to three cues presented on every trial, with each cue carrying a predictive value. Participants are assigned to different curriculum conditions: one group (singleton) is exposed to single cues during training, while another group (parallel) learns with three cues presented simultaneously. Within the parallel condition, the study further varies the training curricula by manipulating the distribution of summed predictive values of cues, the progression in the number of cues per block (increasing or decreasing), and the nature of feedback (deterministic vs. stochastic). Results show that participants in the singleton group learn cue-outcome associations more accurately and rapidly, and generalize better to novel three-cue combinations. To explain these findings, the authors propose a boundedly rational model that assigned credit independently to single cues, implementing the assumption that participants can only focus on one cue at a time. Using this model, they identify optimized training curricula for the parallel group that enhance learning outcomes and find that such a curriculum indeed improves learning performance comparable to that of the singleton group. The study concludes that humans benefit from curricula that support a divide-and-conquer learning strategy, with performance gains emerging from cognitively bounded mechanisms for credit assignment.

Strengths

The study has several strengths that make it a valuable contribution to the literature. First, it tackles a timely and important question about the cognitive mechanisms that support—and are supported by—different learning curricula in humans. This issue is one that nearly every experimental psychologist confronts in practice when designing training procedures for behavioral tasks, yet mechanistic and formal insights into why certain curricula are more effective than others remain scarce. This study approaches this question through a series of experiments that are simple and cleverly designed to test the underlying hypothesis that humans benefit from a divide-and-conquer strategy when learning to predict outcomes based on probabilistic cues.
Perhaps the most impressive aspect of the study is its use of a computational model not only to explain observed behavioral patterns but also to derive strong, testable predictions about how to optimize training curricula—in this case, for the parallel learning condition. The authors show that, a highly skewed distribution of summed cue values, as identified with the model, can improve generalization performance in the parallel curriculum. By empirically confirming these predictions, the study demonstrates how computational insights into curriculum learning can be used to design better training strategies–

potentially with applications beyond the lab. Additionally, the model's predictions are further validated by experiments showing that manipulating task difficulty over time (e.g., increasing or decreasing the number of cues presented on a trial) does not benefit learning in this task—highlighting that not all curriculum structures are equally beneficial.
Finally, the manuscript is well written, the figures are informative, and the presentation appears accessible to a broad scientific audience.

Weaknesses

The primary weaknesses of the manuscript concern the modeling approach and its limitations in supporting strong inferences about the underlying cognitive mechanisms. While the study introduces a compelling computational model, its evaluation relies on simulations trained to convergence and comparisons to summary-level behavioral outcomes, rather than formal model fitting to trial-by-trial behavioral data. This limits the ability to dissociate competing explanations and fails to leverage the rich within-subject dynamics present in the data. In particular, the modeling framework would benefit from fitting free parameters (e.g., learning rate, decision noise) to participants' actual choices, allowing for more rigorous model comparisons (Major Comment 1). The assumption that participants reach asymptotic performance (when they do not) may obscure key dynamics, such as differences in learning rates across curricula, and prevents the model from capturing meaningful variation during training and testing (Major Comments 1a and 1b). Moreover, the manuscript could be significantly improved by formally specifying its core mechanism of bounded credit assignment while distinguishing it from alternative explanations (Major Comment 1c).
In addition to these modeling concerns, there are some more minor issues related to the statistical treatment of WOE estimates (e.g., reliance on point estimates in t-tests without accounting for estimation uncertainty; see Comment 2), the need to clarify cue presentation frequencies across experiments (Comment 3), and the unaddressed possibility that motivational differences may interact with fatigue in Experiment 2 (Comment 4).
While these concerns—elaborated below—should not detract from the article's substantial strengths, addressing them could further strengthen an already impressive study.

Major Comments

1) Performing model fits to better dissociate alternative hypotheses. One of the study's strengths is its use of a computational model to formalize hypotheses about cognitive mechanisms underlying curriculum learning and to generate predictions for improved curricula that are then tested empirically. However, the current approach to model evaluation—based on summary statistics—limits the ability to differentiate between competing mechanistic explanations of the observed behavior.
The study would benefit from fitting the models directly to participants' trial-by-trial choice data using likelihood-based methods, which is standard practice in modeling learning behavior (e.g., especially reinforcement learning models). Instead of training the model to convergence, this would involve fitting free parameters (e.g., learning rate, decision noise) to each participant's choice series and comparing models based on fit metrics that also account for model complexity (e.g., AIC, BIC, or cross-validated likelihood). Such an approach would provide a more rigorous test of the model and allow the authors to evaluate individual by individual which model fits better, potentially exposing relevant individual differences. In addition, such an approach would address several related concerns:

1a) The current model simulations assume convergence, which does not seem to be supported by the empirical data. In particular, performance in the parallel group does not appear to saturate during training (e.g., see Figure 2E). This matters because, under convergence assumptions, differences in learning rate between models have no effect. However, an alternative explanation for the observed performance gap between the singleton and parallel groups could be that participants in the parallel condition learn more slowly—perhaps because multi-cue trials increase cognitive load and reduce effective learning rates. While this hypothesis may ultimately be incorrect, it is important to rule it out. Fitting learning rates for both groups would allow to test whether performance differences can be accounted for by slower learning alone, or whether curriculum structure provides an additional benefit. Alternatively, the authors could demonstrate within their current simulation framework that varying the learning rate in the optimal learner does not yield benefits from highly skewed cue distributions unless the boundedly rational mechanisms are in place (but see Major Comment 1c).

1b) The model is not evaluated against rich behavioral dynamics in the training phase. Currently, model predictions are compared primarily to test performance. However, the human data from the training phase contain several informative patterns that could be used to further evaluate the model. For instance, while the singleton group shows a performance drop from training to test, the parallel group maintains its level of performance (e.g., Fig 2E)—can the model capture this? Similarly, the highly skewed parallel condition even surpasses the singleton group in training performance (Fig. 4C). Additionally, the ascending and descending curricula diverge during training (e.g., the ascending curriculum stagnates earlier; Fig. S4C), which the current model cannot account for under convergence assumptions (see Major Comment 1a). Even if the model fails to capture these effects, attempting to fit them would highlight its limitations and could inspire needed model refinements for future work.

1c) The boundedly rationality model's credit assignment assumptions would benefit from clarification and comparison. The model is said to associate outcomes with single cues rather than integrating across them. E.g., it is stated that "the model determines [the outcome] y based solely on cue i, rather than integrating evidence across all cues" (p. 26). However, this statement is somewhat ambiguous and would benefit from formalization—ideally with an explicit equation. Moreover, several different interpretations of this assumption are possible, each corresponding to a distinct cognitive mechanism:
(a) Participants attend to a single cue (e.g., the most informative one) and base their decision on it, updating only that cue.
(b) Participants integrate multiple cues when making a decision, but update only one (e.g., the most informative) cue per trial.
(c) Participants integrate multiple cues when making decisions and update all cues independently (which seems to be

aligned with the current model's implementation).
These different mechanisms could lead to different behavioral patterns and may be supported or refuted by different aspects of the data. A proper model comparison would help distinguish them to better understand which boundedly rational mechanisms underlie curriculum learning.

2. Potential inflation of Type I error in t-tests on model-derived estimates. The study performs second-level t-tests on subjective weights of evidence (WOE) estimated via first-level logistic regressions of the cue weights for individual participants. This approach can be problematic because the t-test treats the WOE estimates as point estimates, ignoring the uncertainty associated with the initial estimation of the cue values via the logistic regression. This disregard for estimation variance can inflate Type I error rates and, in the worst case, lead to false positives when comparing WOE values across conditions. There are several ways the authors could address this issue. E.g., they could apply a Bayesian hierarchical regression which would allow for simultaneous modeling of within-subject uncertainty and between-group differences. Alternatively, they could weight each participant's WOE estimate in the t-test by the inverse of its standard error to account for differing levels of estimation reliability.

3. Clarification of WOE distribution comparability between Experiment 4 and Experiments 1–3. Experiment 4 applies a skewed distribution of the summed weights of evidence (WOEs). However, it's unclear whether the cue value distributions in both conditions of Experiments 1–3 are directly comparable to those in Experiment 4. If the underlying cue frequencies differ substantially this could confound the interpretation of results, as differences in WOE might then be driven by cue exposure frequency rather than by curriculum structure per se. While I suspect that the cue presentation frequencies are, in fact, matched across experiments, this is not clearly stated in the manuscript (unless I missed it). The right panel of Figure 4A suggests matching within Experiment 4 conditions, but not necessarily across experiments. Explicitly confirming the latter would help rule out alternative explanations

4. Potential interaction between motivation and cognitive fatigue in Experiment 2. Experiment 2 tests whether the performance differences between the singleton and parallel groups can be attributed to differences in motivation by including a trailing task at the end of the experiment. The finding that both groups perform equally well on this added task is interpreted as evidence that motivation does not differ between conditions. However, I wonder whether the addition of two extra blocks may have enhanced cognitive fatigue toward the end of the experiment, potentially masking motivational differences that were present earlier on. While this possibility is speculative, it leaves open the alternative interpretation that initial performance differences between groups may still reflect early motivational differences—effects that may diminish over time due to fatigue or disengagement. One way to address this would be to examine whether performance differences between groups change over time, particularly during the last Testing Phase. If motivation-related differences were present early on but faded due to fatigue, one would expect a convergence in performance between conditions in the final blocks of the Testing Phase. Alternatively, the authors could rule out this explanation by showing that models incorporating (motivation-related) differences in learning rate (see Major Comment 1a) do not account for the data as well as the proposed boundedly rationality model.

Minor Comments

- P. 18: It might be worth adding a table or figure to illustrate the distribution of WOEs in Experiment 1 for the parallel condition.

- P. 25: The authors might want to clarify whether the testing phase of Experiment 5 was still stochastic (and thus comparable to the other experiments).

- The manuscript would benefit from differentiating exploratory from confirmatory hypotheses (the latter of which pertain to the predictions of the boundedly rationality model) statistically, and applying appropriate constraints to hypothesis testing (e.g., two-tailed vs. one-tailed t-tests).

*****
Following suitable revisions, you may want to consider transferring your manuscript. Although we cannot publish your paper, it may be appropriate for another journal in the Nature Portfolio. If you wish to explore the journals and transfer your manuscript please use our manuscript transfer portal. You will not have to re-supply manuscript metadata and files, unless you wish to make modifications. For more information, please see our manuscript transfer FAQ page.

Version 1:

Decision Letter:

Dear Chris,

We have now fixed the technical issue and reopened the link for your resubmission. Here it is:
Link Redacted

** This url links to your confidential home page and associated information about manuscripts you may have submitted or be reviewing for us. If you wish to forward this email to co-authors, please delete the link to your homepage first **

Please remember to submit also a separate point-by-point response letter along with your revised manuscript. If we feel that a substantial attempt has been made to address the referees' comments, this response will be sent back to the referees - along with the revised manuscript - so that they can judge whether their concerns have been addressed satisfactorily or otherwise.

When revising your paper:

- ensure it complies with our format requirements as set out in our Guide to Authors.
- state in a cover note the length of the text, methods and figure legends; the number of references and the number of display items.

Please ensure that all correspondence is marked with your Nature Human Behaviour reference number in the subject line.

We hope to receive your revised paper within four weeks. If you cannot send it within this time, please let us know so that we can close your file. In this event, we will still be happy to reconsider your paper at a later date so long as nothing similar has been accepted for publication at Nature Human Behaviour or published elsewhere in the meantime. Should you miss the four-week deadline and your paper is eventually published, the received date will be that of the revised, not the original, version.

I look forward to hearing from you soon.

Best regards,

[redacted]
Nature Human Behaviour

Version 2:

Decision Letter:

7th January 2026

Dear Dr Summerfield,

Thank you once again for your manuscript, entitled "Human curriculum learning of a cue combination task," and for your patience during the peer review process.

Your manuscript has now been evaluated by 2 reviewers, whose comments are included at the end of this letter. Although the reviewers find your work to be of interest, they also raise some important concerns. We are interested in the possibility of publishing your study in Nature Human Behaviour, but would like to consider your response to these concerns in the form of a revised manuscript before we make a decision on publication.

In particular, we ask that you address the concern expressed by Reviewer #1 about the framing of the results as predictive when the model has been updated following new experiments.

Finally, your revised manuscript must comply fully with our editorial policies and formatting requirements. Failure to do so will result in your manuscript being returned to you, which will delay its consideration. To assist you in this process, I have attached a checklist that lists all of our requirements. If you have any questions about any of our policies or formatting, please don't hesitate to contact me.

In sum, we invite you to revise your manuscript taking into account all reviewer and editor comments. We are committed to providing a fair and constructive peer-review process. Do not hesitate to contact us if there are specific requests from the reviewers that you believe are technically impossible or unlikely to yield a meaningful outcome.

We hope to receive your revised manuscript within two months. I would be grateful if you could contact us as soon as possible if you foresee difficulties with meeting this target resubmission date.

With your revision, please:

• Include a “Response to the editors and reviewers” document detailing, point-by-point, how you addressed each editor and

referee comment. If no action was taken to address a point, you must provide a compelling argument. When formatting this document, please respond to each reviewer comment individually, including the full text of the reviewer comment verbatim followed by your response to the individual point. This response will be used by the editors to evaluate your revision and sent back to the reviewers along with the revised manuscript.

• Highlight all changes made to your manuscript or provide us with a version that tracks changes.

• EXTENDED DATA FIGURES
When re-submitting your manuscript, please ensure that any supplementary figures and tables that are crucial to the manuscript's conclusions are converted into Extended Data figures and tables to increase visibility of these data. Extended Data figures and tables are online-only (present in the online PDF and full-text HTML versions of the paper), peer-reviewed display items that provide essential background to the article but are not included in the main article due to space constraints. A maximum of ten Extended Data display items (figures and tables) is permitted.

Please use the link below to submit your revised manuscript and related files:

Link Redacted

Note: This URL links to your confidential home page and associated information about manuscripts you may have submitted, or that you are reviewing for us. If you wish to forward this email to co-authors, please delete the link to your homepage.

We look forward to seeing the revised manuscript and thank you for the opportunity to review your work. Please do not hesitate to contact me if you have any questions or would like to discuss these revisions further.

Sincerely,

[redacted]
Nature Human Behaviour

Reviewer expertise:

Reviewer #1: Decision making, credit assignment, computational models

Reviewer #3: Curriculum learning, cognitive control, computational neuroscience

REVIEWER COMMENTS:

Reviewer #1 (Remarks to the Author):

The reviewers did a thorough revision of the paper in response to the reviewers' concerns, resulting in a fully novel theoretical framework, as well as one novel experiment. I appreciate the effort to improve the paper, in particular the modeling, in response to our major concerns; the novel experiment helps frame the generalizability of the findings, making the authors broad claims more in line. Given that important parts of the paper are changed, I am treating them like a "first round" review. I am satisfied with responses and edits to the model-independent parts of the results. The novel theoretical framework is much more valid than the previous, which results in a major improvement in the paper. There remain important limitations to the modeling, which I detail below; as well asother issues.

Model comparison
- AIC is not an approximation of model evidence, and thus should not be used to compute protected exceedance probability (contrary to BIC). The results as presented are sufficient without the inclusion of pxp.
- Fig. S8: the model recovery procedure is inadequate and inflates the recoverability results (both model identifiability and parameter identifiability). Specifically, averaging out over simulations as is done here (to decrease simulation noise) leads to a model comparison procedure that is not the same as that applied to the experimental data. Most (reasonable) models will be very recoverable given enough data to average over; the recovery procedure is meant to ask whether the model is recoverable *in the given experimental context* and consequently is not meaningful when done in a different context, with different data size. Since the results of this paper rely strongly on interpretation of model comparison, it is important to ensure that model comparison is interpretable; the recovery (both model and parameters) should be redone applying the same procedure as for experimental datasets.
- Fig. S14 the hybrid model used for the main results (if I understand correctly) in the paper has a significantly worse fit than marginal/joint/hybrid models with attention. My understanding that this is the model used for the main results comes from other figures showing main model parameters, not including attention weights; if I am mistaken, please make sure to clarify. If I am not mistaken, then this is problematic and needs to either be changed (so the main text reflects the results from the

actual winning model) or the rationale for picking the *4th* best winning model should be defended

Model rationale and interpretation.
- Hybrid model– l 34. The authors discretize the mixture of experts in the model, according to a discrete threshold, and only justify this by saying “for simplicity”. This is not obvious to me – most other mixture approaches do not do this discretization and are not particularly complex. This appears to be an ad hoc choice for the modeling that is not a priori likely and is not justified. The authors should clarify the reasons behind this choice, and explain whether it is core to the model (i.e. whether relaxing that assumption to the standard formulation changes the results/fits worse).
- The novelty claim for dynamic updating in the discussion (l13—14 p17) is quite wrong, as many other mixture processes in published papers include dynamically changing mixture weights, for example Miller, Shenhav & Ludvig 2019, Collins & Frank 2012, Frank & Badre 2012, and many others.
- It seems very misleading that parts of the paper (e.g. follow up experiments, predictions) are claimed as model predictions, when we know that the model has been fully changed after this revision (e.g. p13/14). If the authors feel that their theory made the prediction independently of the model implementation, then they should express this more accurately and justify it; if it doesn’t, then they should refrain from making prediction claims where they are in fact postdictive.

Statistical issues
- Fig. S3, right panel: it is very puzzling that the authors report no effect of condition on RTs (p=.5), when the plot appears to show a clear effect. This should be at minimum double checked; and furthermore, is it possible that an inappropriate test is used that does not factor out relevant variance, limiting the power in testing for condition? The use of this plot to argue against a speed accuracy tradeoff is not convincing: the null result looks like a type II error that is over interpreted.
- P54 decay, please include the statistical test used and result. If it is a t-test as elsewhere, is it appropriate for this parameter, that likely has a skewed distribution? In any case, this again appears to lead to an over interpretation of a null result: decay has a multiplicative effect on learning rates, such that the numerically higher rate plotted in the figure could lead to significantly faster learning rate decrease in parallel than singleton. To conclude about motivation difference, it would be better to verify the effect on learning rates more directly.
- More generally, the use of parametric stats is problematic in several places in the manuscript. In addition to the above two examples, see e.g. Fig. S15 d, where one of the measures is bounded, with many points at the bounds (0) leading to a very skewed (non-normal) distribution; linear regression inflates the results, a non-parametric (e.g. Spearman) or robust test should instead be used.

Minor points:
- Fig. S17 e, the x axis tick labels are incorrect
- Methods p37 for model simulation appear to not have been updated from the previous version (they still contain the previous modeling with 100 epochs).

Reviewer #3 (Remarks to the Author):

The authors have provided a thorough and commendable revision that substantially strengthens the manuscript. Most notably, the transition from snapshot-based simulations to trial-by-trial likelihood-based model fitting improves the modeling framework’s ability to capture learning dynamics and supports more rigorous inference about the mechanisms underlying curriculum learning. The inclusion of model recovery and parameter recovery analyses is particularly valuable, as it allows for stronger conclusions about which models, such as hybrid, serial, or optimal accounts, best explain the data and justifies interpretation of parameters in terms of individual or experiment differences. In addition, the authors have systematically formalized and tested a broad range of alternative explanations raised by the reviewers, including differences in learning rates, learning rate decay, and attentional weighting mechanisms. The addition of explicit equations and clearer descriptions of the learning rules substantially improves the clarity with respect to the modeling approach. Overall, these new analyses yield informative insights into how curriculum structure shapes the balance between learning precision and cognitive cost over the course of learning.

Taken together, the revision includes an impressive array of additional analyses that address all of my concerns from the original review and make this study a strong and laudable contribution to the literature.

While I do not see any issues that would preclude publication, I have a small number of minor suggestions that the authors may wish to consider if they feel these would further improve the paper.

1. Predictive vs. generative model performance. It would be helpful to clarify explicitly whether the reported model simulations in Figures R6a and main-text Figures 3e, 4d, 5d, as well as Figures S7b and S7d reflect predictive performance (conditioning on the model on participants’ previous choices to predict the next trial) or generative performance (simulating the model from scratch using fitted parameters). I suspect the latter, but I could not find a clear statement in the manuscript. This distinction is important, as generative simulations provide a stronger posterior predictive check, whereas predictive simulations can overstate a model’s ability to capture learning dynamics in response to the task environment (see Palminteri et al., 2017, in TiCS).

2. Scope of model recovery analyses. The model and parameter recovery analyses are a very welcome addition, but I

wondered why recovery was demonstrated for only three of the five models listed in Table R1. In particular, it could be informative to explicitly test distinguishability between e.g., the hybrid SD model and the hybrid constant model. Similarly, extending recovery analyses to the alternative models incorporating learning rate decay or attentional weights could further strengthen claims about model distinguishability and ability to rule out corresponding alternative hypotheses. That said, I recognize the practical constraints involved, and this comment is intended as a suggestion rather than a requirement.

3. Interpretation of joint updating in the parallel condition. One result that I found somewhat counterintuitive concerns Figure R7a, where the hybrid SD model appears to infer fewer trials with joint updating in the parallel condition than in the singleton condition, despite participants benefiting more from joint updating in the parallel condition. I may be missing some detail, but a brief explanation of how this pattern should be interpreted would likely help both reviewers and readers.

4. Model comparison criteria. The rebuttal primarily reports AIC- and NLL-based model comparisons. Given the richness of the modeling effort, it might be helpful to briefly justify the choice of AIC over alternatives such as BIC, particularly with respect to model differentiability (e.g., does AIC provide better model differentiability than BIC?; see Wilson & Collins, 2019 in eLife). Even a short note indicating that the main conclusions are robust across criteria would be reassuring.

5. Reporting fitted parameter distributions. It would be useful to report the distributions of fitted parameters for the final selected model, perhaps in the supplementary materials. This would allow readers to verify that parameters such as α^+ and α^- lie well within their predefined bounds, such as the [0, 1] interval, and would further rule out potential fitting pathologies that are not uncommon in models of this kind.

6. Across-experiment comparisons. Finally, since models were fit across multiple experiments (assuming data was pooled for model comparison), I was curious whether different models might better explain data from different experiments (e.g., whether Experiment 1 favors a different winning model than Experiment 2). This comment is offered primarily out of my own interest, and I do not expect additional analyses unless such results are already available.

Version 3:

Decision Letter:

Our ref: NATHUMBEHAV-25020896C

3rd March 2026

Dear Dr Summerfield,

Thank you for submitting your revised manuscript "Human curriculum learning of a cue combination task" (NATHUMBEHAV-25020896C). It has now been seen by the original referees and their comments are below. As you can see, the reviewers find that the paper has improved in revision. We will therefore be happy in principle to publish it in Nature Human Behaviour, pending final revisions to satisfy the referees' remaining requests and to comply with our editorial and formatting guidelines.

We are now performing detailed checks on your paper and will send you a checklist detailing our editorial and formatting requirements within two weeks. Please do not upload the final materials and make any revisions until you receive this additional information from us.

Please do not hesitate to contact me if you have any questions.

Sincerely,

[redacted]
Nature Human Behaviour

Reviewer #1 (Remarks to the Author):

I thank the authors for being responsive to the reviews, and most points have been well addressed, the study is well done

and results are interesting.

I am still not fully comfortable with the general framing. My previous point 6 on claims around modeling is still not fully addressed. Despite the two edits in the result, mutliple places in the paper still overstate the claims of prediction by the *cognitive model* as opposed to the broader theory.

For example, the abstract still over claims predictiveness of the model; however, it is not the cognitive model, as stated, that was used to accelerate human learning. The authors use “framework” in their answer to my previous point 6, and adequately revised the results section, but not the abstract.

Same, the paragraph starting l 337 is still misleading. e.g . L347-351 implies simulations from the presented cognitive model generated the predictions, which is not the case. Again, there should be no confusion between the high level theory (which made accurate predictions) and the quantitative cognitive model presented in the paper (which was not predictive, and is instead postdictive). Same l 397, l433, l449 etc. I can’t list them all, but this should be adjusted throughout.

I agree with the authors that in the end what matters is that the theory predicts the results; but the results as framed overstate the methodological use of the quantitative model simulations for generating predictions, which is an unsupported claim in this paper. I do not see this as a minor language point, but an important point that overstates the contributions of the paper.

Reviewer #3 (Remarks to the Author):

I commend the authors for their thorough second revision. They have addressed all of my previous concerns, and I have no substantive issues remaining. The only minor point is that the statement "joint updating is more cognitively demanding" might be phrased more cautiously. Given the lack of definitive evidence, it would be preferable to frame this as a hypothesis, e.g., "joint updating is assumed to be more cognitively demanding", rather than as an established fact. With this final clarification, I believe the manuscript makes a valuable contribution and is ready for publication.

Open Access This Peer Review File is licensed under a Creative Commons Attribution 4.0 International License, which permits use, sharing, adaptation, distribution and reproduction in any medium or format, as long as you give appropriate credit to the original author(s) and the source, provide a link to the Creative Commons license, and indicate if changes were made.
In cases where reviewers are anonymous, credit should be given to 'Anonymous Referee' and the source.
The images or other third party material in this Peer Review File are included in the article’s Creative Commons license, unless indicated otherwise in a credit line to the material. If material is not included in the article’s Creative Commons license and your intended use is not permitted by statutory regulation or exceeds the permitted use, you will need to obtain permission directly from the copyright holder.
To view a copy of this license, visit https://creativecommons.org/licenses/by/4.0/

Reviewer #1:

0. In this article, Mi and Summerfield investigate the role of curriculum learning in a version of the weather prediction task, a multi-cue binary probabilistic learning task. They first present 3 experiments establishing the finding that "curriculum learning" (breaking down the problem into parts) helps learning. They then propose a "bounded rationality" neural network framework to show qualitative similar behavior. Finally, they use this framework to design novel curricula which they predict should enhance or impair learning, and test this in two final experiment.

   This paper is well written, investigates a question of broad interest, and uses carefully designed behavioral experiments to test it, with rigorous methods. However, major issues limit the value of this contribution, including issues with a) ruling out alternative hypotheses; b) testing competing mechanisms; and c) supporting the proposed theoretical interpretation. Furthermore, the generalizability of the findings is very limited.

   Reply: We thank the reviewer for the thoughtful and constructive feedback. We appreciate the recognition of our research question, experimental design, and methodological rigor. The reviewer's concerns about alternative hypotheses, competing mechanisms, and theoretical interpretation are important, and we have addressed them carefully in the revision by greatly extending our modelling framework. Below, we briefly summarize how we have addressed the reviewer's main concerns (with detailed responses provided under each specific question).

   The first major concern relates to our initial modelling framework, which previously offered qualitative predictions of final performance and did not capture learning dynamics (Question 1). To address this, we (i) introduced a hybrid learning framework that allows participants to arbitrate between two different learning strategies across training trials (Figs. R1–4), (ii) fitted the model to participants' trial-by-trial choices (Figs. R5–7 and Table R1), and (iii) conducted model and parameter recovery to assess model reliability (Fig. R8). The hybrid learning model explained participants' behaviour better than single-strategy alternatives. Moreover, this model reproduced participants' trial-by-trial learning dynamics quite well and could be clearly distinguished from alternative models with reliably estimated parameters. Together, these results validate our revised modelling framework and provide interpretable trial-by-trial learning trajectories alongside final performance.

   The second concern pertains to the lack of competing mechanisms (Question 2). In response, we systematically compared the hybrid learning model with several alternative models to rule out competing hypotheses. First, the hybrid learning model consistently outperformed single-strategy models in both conditions. Second, in singleton condition, behaviour could not be better explained by either the absence of learning during three-cue trials or by assuming different learning rates for single- and multi-cue training (Fig. R9). Third, behaviour in both conditions was not captured by models in which updating strategy depended solely on reward outcomes or trial number (Fig. R10a). Fourth, learning performance depended on the trial-by-trial assignment of updating strategies, not merely on the overall proportion of each learning strategy (Fig. R10b). Finally, although incorporating learning-rate decay (Fig. R11) or attentional biases (Fig. R12) improved model fits, the hybrid learning model still provided better account of participants' behaviour than single-strategy alternatives.

The third concern involves the interpretation of Exp. 2 and 3 (Question 3). We have now clarified the aims of the two experiments and provided additional evidence showing that the singleton advantage is unlikely to result from motivational differences between conditions. Regarding the fourth concern about discrepancies between model predictions and empirical results in Exp. 6 (Question 4), the revised model now aligns closely with the observed data (Fig. R13). In response to Question 5, we have expanded the participant information in the Methods section to include age, gender, and the number of participants tested and excluded. Finally, for Question 6, concerning the generalizability of our findings, we have extended our main results of the curricula to a new compositional task (Fig. R14), validated the modelling framework rigorously, and added a broader discussion about the hybrid learning framework, the "divide-and-conquer" strategy, and the implications for curriculum design in human and AI learning.

We believe these revisions based on the reviewer's insightful comments, have substantially strengthened the manuscript and clarified its theoretical contribution. We thank the reviewer again for the helpful feedback and would be glad to address any further questions.

Major issues

1. The most important issue is in the modeling framework. Model methods state that the network is trained for 100 epochs (each corresponding to the full empirical experiment) to make predictions; predictions come from the asymptotic performance phase (top of p27). To rephrase, it means that the network is exposed to 2 orders of magnitude more trials than the participants, and not in the same order, despite the whole point of the paper being to investigate the role that the learning regime impacts learning. This approach seems very misleading, as most would read this paper and interpret it as saying that the authors offer an explanation of how learning dynamic unfolds. Instead, they propose a fixed snapshot of what might be known at the end of learning, with no validly interpretable explanation of how the human participants might arrive there. To be interpretable, the framework should lead to interpretable predictions over the same training as participants, and capture learning trajectories as well as testing phase.

   Reply: We thank the reviewer's excellent suggestion for improving our model fitting, which we have now implemented in full. This new modelling approach allows us to train the model with the same trials as the participants and to quantitatively capture the trial-by-trial learning dynamics. This question is closely related to some aspects in Question 1 of Reviewer 3, so we combine these questions and answer them below.

   The reviewer points out that our initial approach - using model behavior at convergence to predict participants' choices - was limited, as it only provided qualitative predictions of final performance while failing to capture learning dynamics and individual variability. In response, we have substantially revised the modeling framework. Specifically,

   (i) We have extended our modelling framework to a hybrid learning framework which includes a mixture of 'marginal' and 'joint' updating strategies, corresponding to the 'serial' and 'optimal' updating described in our initial submission respectively.

   (ii) We have performed model estimation to fit participants' choices on a trial-by-trial basis.

   These revisions allow the model to be trained with the same trials as participants, which could provide interpretable trial-by-trial learning trajectories alongside final performance. We provide detailed descriptions below.

(i) A hybrid learning framework

We developed a hybrid learning framework which integrates the two learning strategies described in our initial submission: marginal updating, which assigns credit to each cue independent of every other, and joint updating, which distributes credit accurately across cues based on the combined evidence of all presented cues (Fig. R1c; see Fig. R1e for a more detailed illustration; see also Fig. R1a and R1d for network structure and updating rule). In this framework, the contribution of each learning strategy is determined by a weighting parameter γ. A higher γ reflects a greater reliance on joint updating, whereas a lower γ indicates the converse.

We considered two forms of hybrid learning. In the hybrid constant model, γ remained fixed between 0 and 1 across all training trials, whereas in hybrid adaptive models, it varied dynamically depending on the trade-off between learning precision and cognitive demanding of each learning strategy. In theory, γ could change continuously from 0 to 1 as a function of the learning precision and cognitive demanding. However, for simplicity, γ was set to either 0 (i.e., marginal updating) or 1 (i.e., joint updating) in each trial for the hybrid adaptive models, and the results remained similar with a continuous γ.

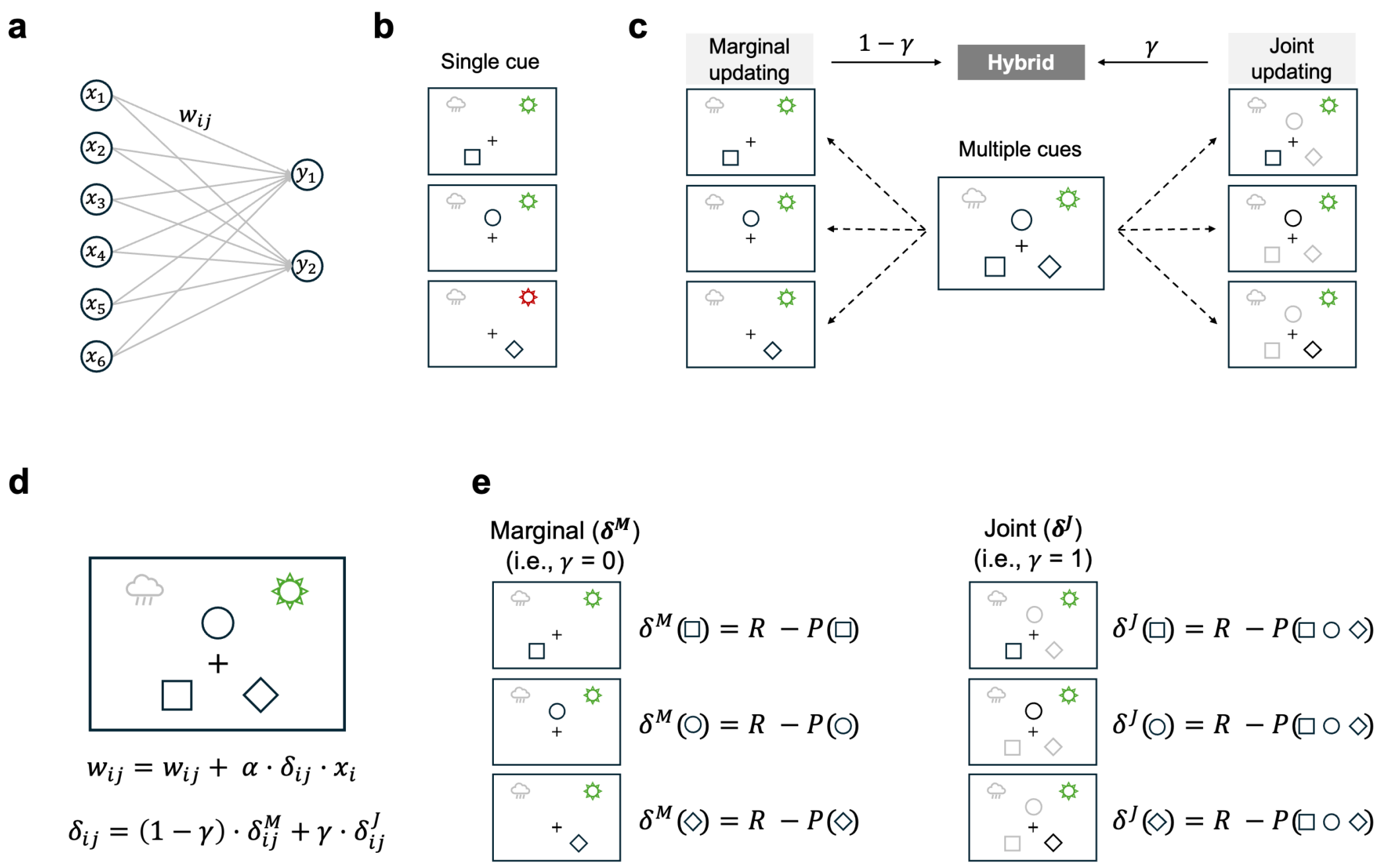

Fig. R1. Illustration of marginal, joint and hybrid updating. (a) Network structure. (b) Credit assignment in one-cue trials. (c) Credit assignment in multi-cue trials. In marginal updating, the outcome is assigned to each cue independently, as if that cue alone determined the result. In joint updating, cue weights are updated using the integrated prediction from all presented cues on the trial. In the hybrid updating, the two rules are combined with a weighting parameter γ. (d) Equations for how the weight of cue i is updated given the chosen option j. The rule reduces to marginal updating when γ = 0 and to joint updating when γ = 1. (e) Illustration of prediction errors (PEs) for marginal and joint updating. Take the cue diamond as an example. Under marginal updating, the PE for the diamond cue equals the observed reward minus the reward probability predicted by the diamond alone. Under joint updating, the PE equals the observed reward minus the reward probability predicted from the integrated evidence of the square, circle, and diamond. The observed outcome is identical in both cases; only the predicted outcome differs.

Specifically, compared to joint updating, marginal updating reduces cognitive demands for learning, as it obviates integration of evidence across cues when updating the weight of each cue. However, marginal updating also sacrifices learning precision: the learnt WOE

value is driven by the marginal reward probability of each cue and may deviate substantially from the true objective WOE value when marginal updating is made under multi-cue training (see Fig. R2). In Fig. R2a, the orange lines represent the model learnt WOE values at convergence for marginal updating, which mirror the empirical WOE values calculated by the marginal reward probability of each cue in Fig. R2b. By contrast, although joint updating is more cognitively demanding, it ensures that weights are updated in a way that converges on the true WOE values for each cue regardless of the training contexts (Fig. R2a). In Fig. R2a, the purple lines represent the model learnt WOE values at convergence for joint updating, which overlap with the true WOE values in both training contexts. In Fig. R2a left panel, the orange and purple lines overlap. The variances across those thin lines in Fig. R2a indicate the stochasticity of simulation in the probabilistic learning task.**

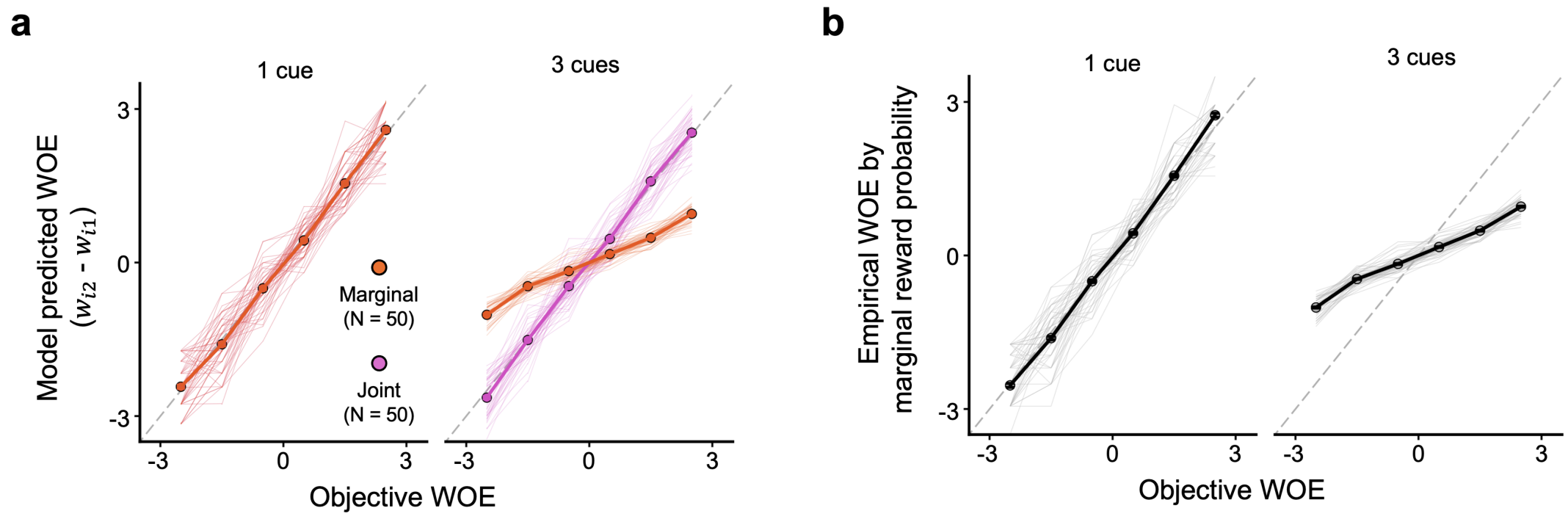

Fig. R2. Results of model simulation for marginal and joint updating at converge (a) and the empirical WOE values (b). The empirical WOE value for each cue is computed based on its marginal reward probability. For each cue, we first estimated its marginal reward probability, P_{rew} — the probability that a right (sunny) option is rewarded given the cue's presence. The empirical WOE was then computed as the log odds of this probability: log(P_{rew} /(1- P_{rew})). For more details, please see our Methods from line 17 page 31 to line 34 page 31 (L17P31–L34P31) in the revised manuscript.

We assume a rational learner to arbitrate between these two learning strategies by comparing their relative learning precision and relative cognitive cost. If the sacrificed learning precision by marginal updating is over its reduced cognitive demands, joint updating is preferred otherwise marginal updating is preferred. We estimated the relative cognitive cost between the two learning strategies for each participant as a free parameter θ and quantified the sacrificed learning precision of marginal updating compared to joint updating in two different ways (Fig. R3 middle and right panels).

(i) Constant weight (Hybrid constant)

$$\delta_{ij} = (1-\gamma)\cdot\delta_{ij}^{M} + \gamma\cdot\delta_{ij}^{J}$$
$$0 \leq \gamma \leq 1$$

(ii) Hybrid based on prediction error (PE) distance (Hybrid PE)

$$\delta_{ij} = \begin{cases} \delta_{ij}^{M}, & if\ |\delta_j^{M} - \delta_j^{J}| \leq \theta \\ \delta_{ij}^{J}, & if\ |\delta_j^{M} - \delta_j^{J}| > \theta \end{cases}$$

(iii) Hybrid based on standard deviation (SD) of subjective WOEs (Hybrid SD)

$$\delta_{ij} = \begin{cases} \delta_{ij}^{M}, & if\ sd(WOE) \leq \theta \\ \delta_{ij}^{J}, & if\ sd(WOE) > \theta \end{cases}$$

Fig. R3. Hybrid learning models. In hybrid constant model, the weight parameter γ is fixed across all training trials. In hybrid prediction error (PE) model, the strategy on each trial is chosen by comparing the marginal and joint updating PEs: if their difference quantified as the Euclidean distance between them is below a threshold θ, the model applies marginal updating; otherwise, it applies joint updating. Hybrid standard deviation (SD) model uses an analogous rule but bases the decision on the SD of the currently learned WOE values across the presented cues: low SD favors marginal updating, high SD favors joint updating. Although the threshold parameters in hybrid PE and hybrid SD models are on different scales, they are expected to be highly correlated.

**First, it is quantified as the difference between the prediction errors (PEs) generated by marginal and joint updating (hybrid PE model; Fig. R3 middle). Larger discrepancies imply

that marginal updating deviates more from joint updating, thereby making joint updating more beneficial. This approach is theoretically rigorous but to some extent unrealistic, as it requires evaluating the PE of joint updating before deciding which strategy to use. Second, it is calculated heuristically as the standard deviation (SD) of the learnt WOE values across the presented cues (hybrid SD model; Fig. R3 right). A higher SD indicates greater disparity among cues, which in turn predicts a larger gap between marginal and joint updating PEs. Indeed, SDs correlate strongly with the PE distances (spearman's ρ = 0.96, P ≈ 0; Fig. R4).

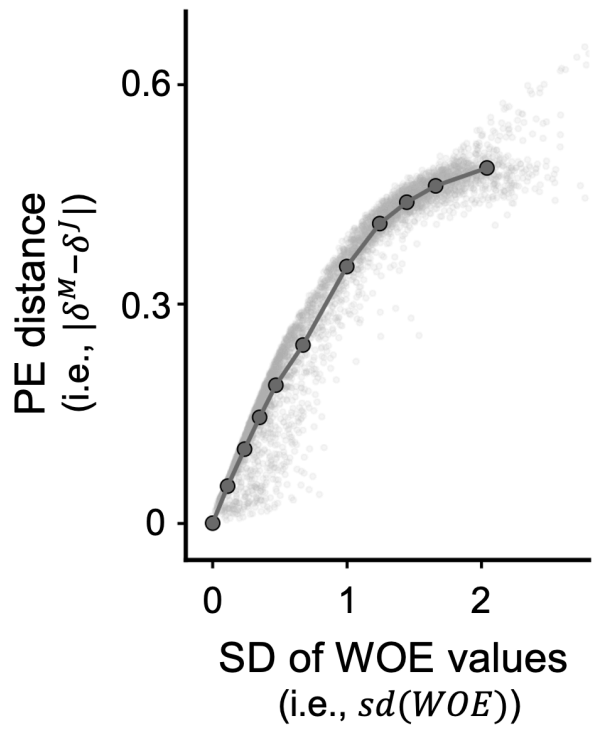

Fig. R4. Correlation between PE distance and SD of WOE values. The SD of WOE values provides a heuristic approximation for the distance between marginal and joint updating PEs.

The intuition behind the hybrid SD model is that the reliability of marginal versus joint updating depends on the disparity in the evidence strength of cues. When cue weights vary widely (i.e., large SD), marginal updating can substantially mislead learning, whereas joint updating avoids such errors. For instance, in Figs. R1b-c, if a square and a circle support "sunny" but a diamond strongly supports "rain", a sunny outcome following the three cues would cause marginal updating to generate a large, misleading prediction error for diamond, incorrectly weakening the diamond-rain association. In contrast, by integrating evidence across all cues, joint updating corrects this imbalance and yields a more accurate update. Conversely, when cue weights are similar (e.g., SD ≈ 0), both strategies converge to nearly identical updates, making marginal updating preferable due to its lower cognitive cost.

This hybrid learning framework is consistent with previous studies that suggest people rely on a mixture of learning strategies in a wide range of learning problems (Charpentier et al., 2020; Daw et al., 2011; Zhu et al; 2012). More importantly, this framework allows participants to flexibly adjust the weight of each learning strategy based on the learning contexts and the acquired knowledge so far, which could help us capture the dynamics of learning strategy in each condition. Such a mechanism is also consistent with the resource-rational account, which has been successfully used to model human cognition as the outcome of balancing the expected reward and cognitive effort of a choice (Bhui et al., 2021; Gershman et al., 2015; Goodman & Frank, 2016; Lieder & Griffiths; 2020).

(ii) Model estimation to establish the hybrid learning framework

We performed model estimation for each of the five models: marginal only, joint only, hybrid constant, hybrid PE, and hybrid SD (Table R1), to see how well these models could capture participants' choices on a trial-by-trial basis, including both training and testing phase. The parameters of each model and the estimation results are summarized in Table R1. α_+ and α_- represent two learning rates for positive and negative outcome respectively. β_1 is the invers temperature for model prediction based on single cues and the invers temperature

for model prediction based on three cues (β_3) is set to 1 to account for the collinearity between β_1 and β_3 if they are both allowed to change. Therefore, a larger β_1 means a greater cost in evidence integration from single cue to multiple cues. γ captures the weighting of joint updating in hybrid models: when γ = 0, the hybrid model reduces to pure marginal updating, and when γ = 1, it reduces to pure joint updating. θ reflects the relative cognitive cost of implementing joint updating compared to marginal updating.

Condition	Models	α_+	α_-	β_1	γ	θ	-LL	AIC
	Marginal	0.37 (0.023)	0.06 (0.012)	1.7 (0.18)			276.5 (6.05)	559.0 (12.11)
	Joint	0.36 (0.028)	0.03 (0.008)	4.5 (0.27)			252.8 (7.76)	511.52 (15.53)
**Singlton**	Hybrid constant	0.43 (0.029)	0.05 (0.010)	3.7 (0.25)	0.80 (0.030)		250.8 (7.69)	509.6 (15.38)
	Hybrid PE	0.45 (0.029)	0.05 (0.009)	3.3 (0.21)		0.30 (0.013)	246.6 (7.74)	501.3 (15.47)
	Hybrid SD	0.43 (0.029)	0.05 (0.009)	3.3 (0.23)		0.97 (0.056)	247.0 (7.72)	502.0 (15.45)
	Marginal	0.18 (0.015)	0.07 (0.012)	2.3 (0.29)			327.1 (5.81)	660.1 (11.62)
	Joint	0.17 (0.019)	0.01 (0.005)				321.3 (7.08)	646.6 (14.17)
**Parallel**	Hybrid constant	0.29 (0.022)	0.06 (0.013)	4.8 (0.38)	0.55 (0.032)		313.2 (6.98)	634.3 (14.00)
	Hybrid PE	0.32 (0.023)	0.08 (0.014)	4.1 (0.33)		0.35 (0.017)	308.3 (7.20)	624.6 (14.41)
	Hybrid SD	0.25 (0.020)	0.06 (0.013)	3.8 (0.32)		0.86 (0.062)	310.0 (7.19)	628.0 (14.37)

Table R1. Summary of modelling results for Exp. 1–3. The value shown in each entry represents the mean (SEM) across participants.

We used the Akaike Information Criterion (AIC) value to quantify how well a model could predict participants' choices while controlling for the model complexity. A lower AIC value indicates a better account of behavior. Our results showed that the hybrid learning models consistently achieved lower AIC values than either single-strategy model in both conditions (Fig. R5), suggesting that participants relied on a mixture of marginal and joint updating. Interestingly, both hybrid PE and hybrid SD models outperformed the hybrid constant model (Fig. R5), indicating that participants dynamically adjusted the weighting between strategies. Further comparisons showed that the hybrid PE and hybrid SD models performed similarly in the singleton condition while the hybrid PE model fit better in the parallel condition (Fig. R5). Nevertheless, because the hybrid SD model is more cognitively plausible, we adopted it as our main hybrid model for all subsequent analyses unless otherwise specified. In fact, similar results were observed regardless of whether a hybrid PE or hybrid SD model was used, as these two models were highly correlated.

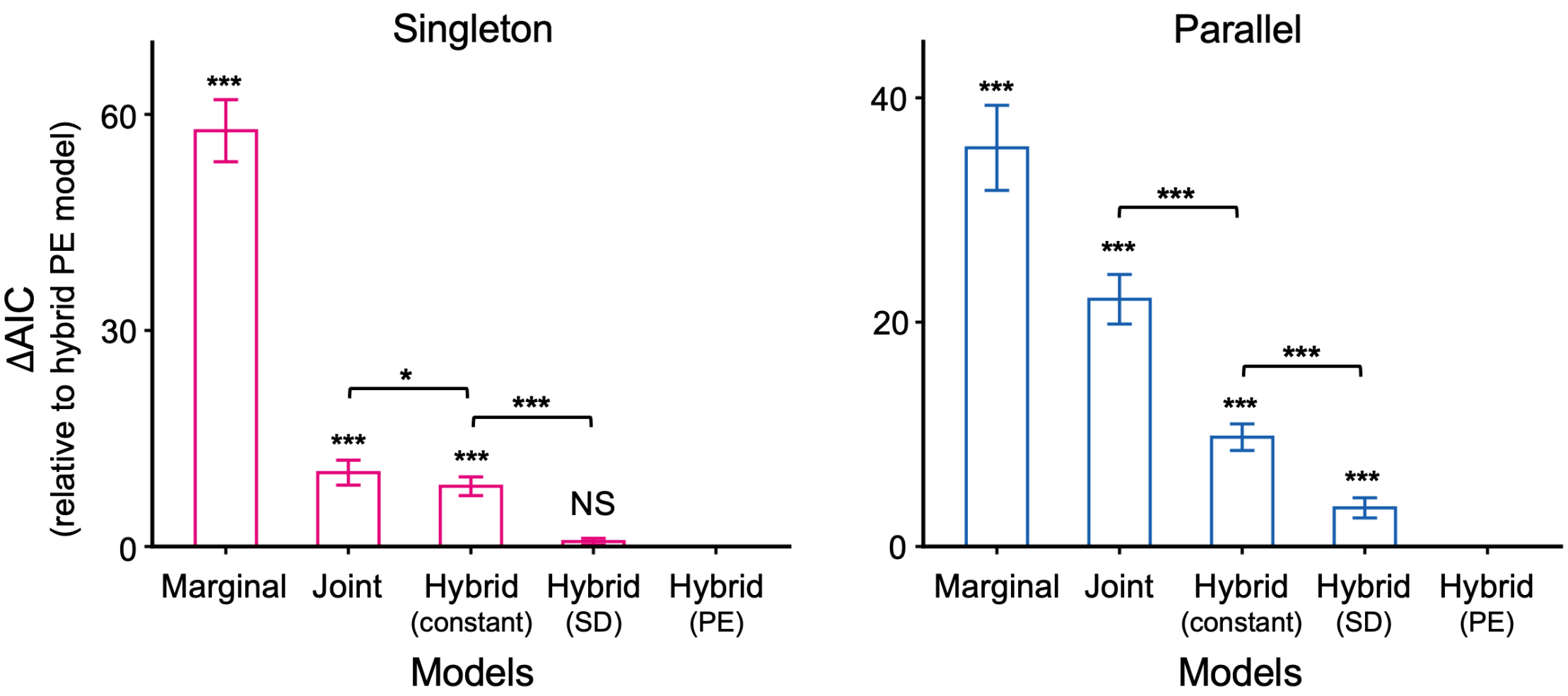

Fig. R5. Comparison between hybrid and single-strategy models. The ΔAIC value of each model is computed as the differences between the AIC value of each model relative to the hybrid PE model. Lower ΔAIC values reflect better explanatory power.

Overall, the hybrid SD model could predict 73.1%±0.96% (mean±SEM) of participants' choices in singleton condition and 65.0%±0.97% of choices in parallel condition. Such a difference in prediction accuracy is due to the choice variabilities in each condition, driven by the curriculum structure. In line with the accurate model predictions, the hybrid SD model greatly recovered the learning curves in both conditions, including the accuracy drop from one-cue to three-cue trials in the singleton condition (Fig. R6a). Moreover, the WOE values learnt by the hybrid SD model also closely matched the subjective WOE values recovered from participants' actual choices through logistic regressions (Fig. R6b). These results indicate that the hybrid SD model captures participants' learning dynamics quite well.

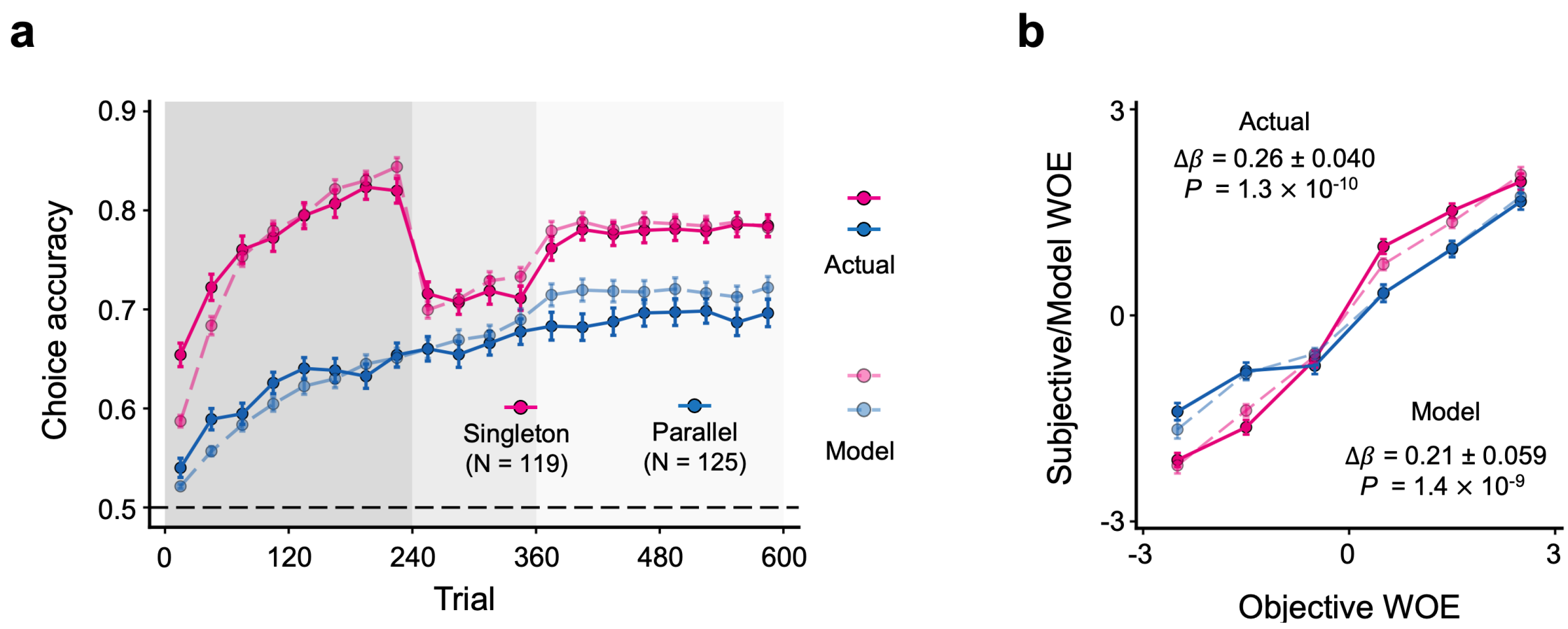

Fig. R6. Actual versus model predicted results. The recovered behavioral patterns in choice accuracy curves (a) and WOE values (b) by the hybrid SD model closely match the empirical results.

More interestingly, we computed the proportion of multi-cue trials with joint updating, as inferred from the hybrid SD model (in one-cue trials, marginal and joint updating cannot be separated). As expected, this proportion increased gradually across training in both conditions but was consistently lower in the parallel than in the singleton condition (Fig. R7a; see also Fig. R7c for example participants). Moreover, in both conditions, the proportion of joint updating trials was positively correlated with choice accuracy (Fig. R7b). Crucially, when we included both the condition indicator and the proportion of joint updating trials in a single regression to predict choice accuracy, the condition effect disappeared

(singleton vs parallel: β = -0.004, SE = 0.0129, t = -0.31, P = 0.755; proportion of joint updating: β = 0.270, SE = 0.0182, t = 14.82, P < 0.001). This is reflected by the two overlapping lines in Fig. R7b, indicating that if participants in the two conditions relied equally on joint updating, then they would achieve similar performance. Together, these results suggest that the observed singleton effect might be explained by the different weights of joint updating across conditions, which in turn are shaped by the training regimes.

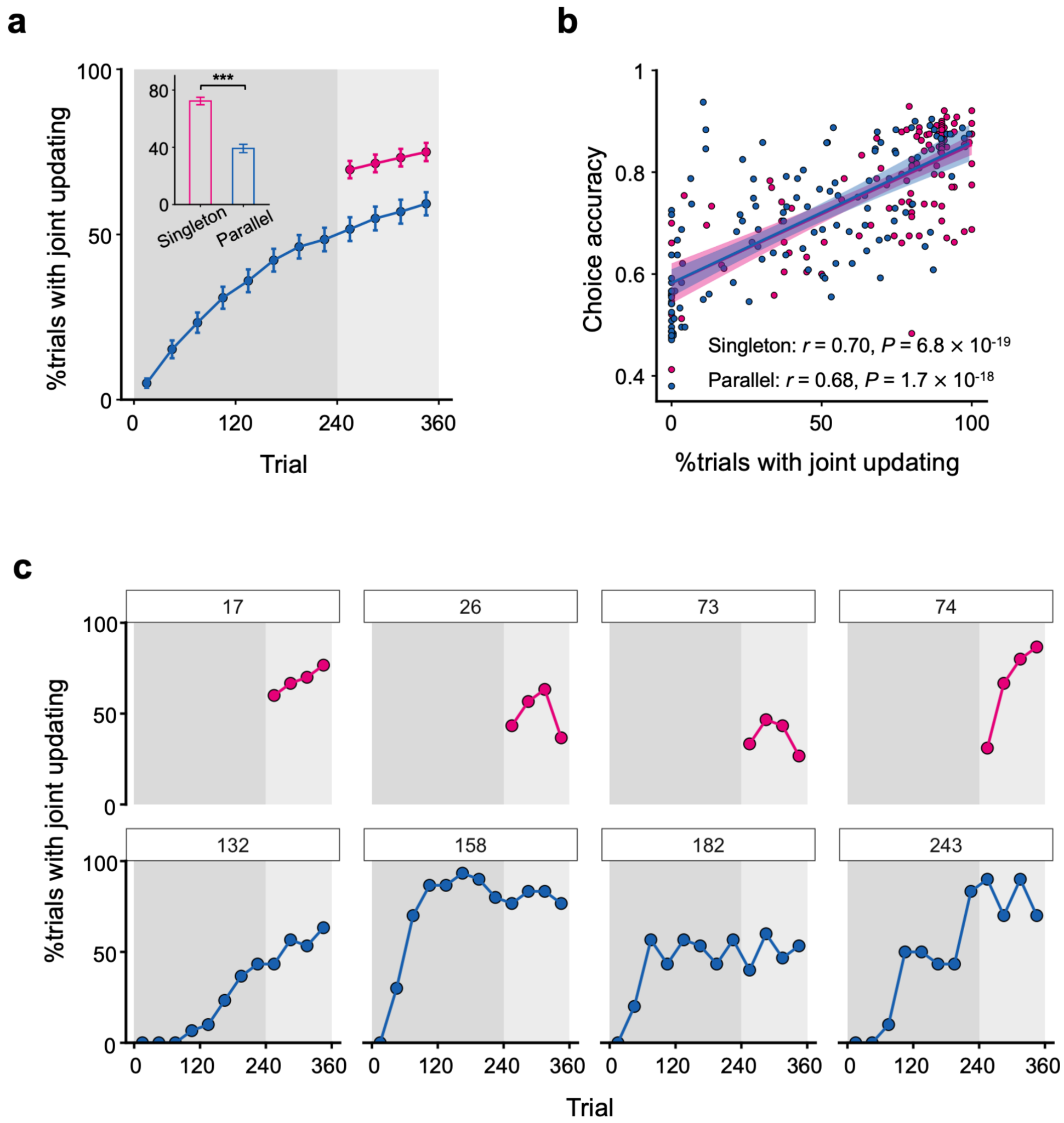

Fig. R7. Dynamics of learning strategy. (a) Proportion of multi-cue trials using joint updating over the course of training; inset shows the mean across all multi-cue training trials. (b) Across-participant correlation between choice accuracy and the proportion of joint updating trials. (c) Example participants.

We also performed model and parameter recovery to show that the hybrid SD model is distinguishable from alternative single-strategy models and that its parameters can be reliably estimated (Fig. R8). Fig. R8a shows that the model used to generate the simulated data is also most likely to be recovered, demonstrating that these models are distinguishable. Figs. R8b–e reveal that the recovered parameter values of the hybrid SD model are strongly correlated with the true values, confirming reliable parameter identifiability.

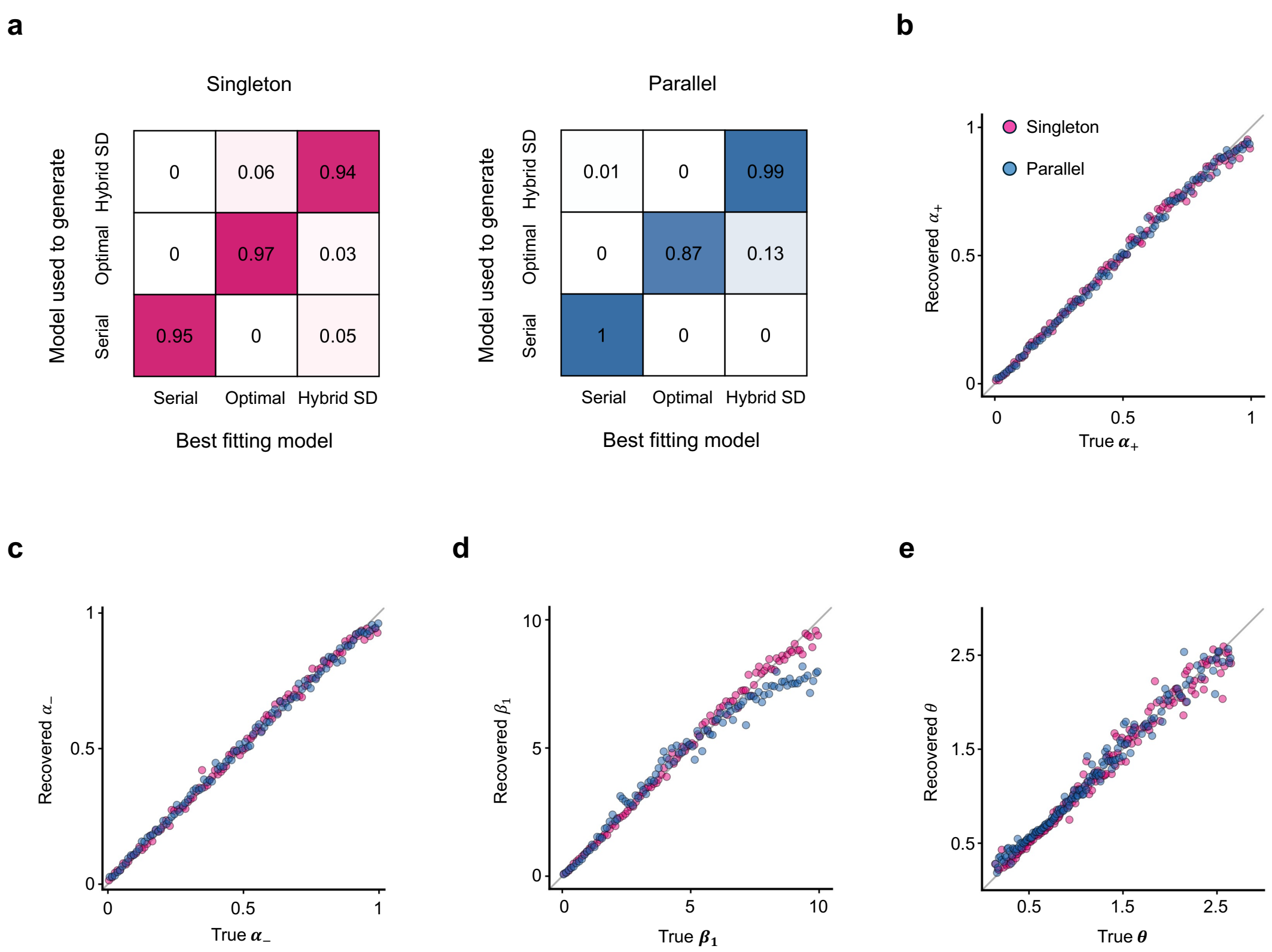

Fig. R8. Results of model and parameter recovery. (a) Model recovery. The number in each cell represents the probability of each candidate model being the best model in capturing the behavioral choices simulated by the model in each row. (b-e) Parameter recovery for hybrid SD model. For all four parameters (α_+, α_-, β_1 and θ), the recovered values are strongly correlated with the true values, confirming reliable parameter identifiability.

Together, our extended modelling framework captures not only the participants' explicit choices but also their latent use of learning strategy on a trial-by-trial basis. We have updated manuscript accordingly (revisions from line 16 page 8 to line 10 page 12 (L16P8–L10P12) in the revised manuscript) and added supporting results in Fig. 3, Fig. S6, Fig. S8, Fig. S11, and Table 1.

- **Charpentier, C. J., Iigaya, K. & O'Doherty, J. P. A Neuro-computational Account of Arbitration between Choice Imitation and Goal Emulation during Human Observational Learning. Neuron 1–21 (2020) doi:10.1016/j.neuron.2020.02.028.**
- **Daw, N. D., Gershman, S. J., Ben Seymour, Dayan, P. & Dolan, R. J. Model-Based Influences on Humans' Choices and Striatal Prediction Errors. Neuron 69, 1204–1215 (2011).**
- **Zhu, L., Mathewson, K. E. & Hsu, M. Dissociable neural representations of reinforcement and belief prediction errors underlie strategic learning. Proc. Natl. Acad. Sci. U.S.A. 109, 1419–1424 (2012).**
- **Bhui, R., Lai, L. & Gershman, S. J. Resource-rational decision making. Curr. Opin. Behav. Sci. 41, 15–21 (2021).**
- **Gershman, S. J., Horvitz, E. J. & Tenenbaum, J. B. Computational rationality: A converging paradigm for intelligence in brains, minds, and machines. Science 349, 273–278 (2015).**
- **Goodman, N. D. & Frank, M. C. Pragmatic language interpretation as probabilistic inference. Trends Cogn. Sci. 20, 818–829 (2016).**
- **Lieder, F. & Griffiths, T. L. Resource-rational analysis: Understanding human cognition as the optimal use of limited computational resources. Behav. Brain Sci. 43, e1-85 (2020).**

2. A secondary major issue comes from the lack of competing models, besides the optimal one. This type of task has been extensively studied and modeled, and approaches like attention-modulated RL models (e.g. Niv and colleagues) or other algorithmic approaches might offer alternative explanations; these explanations are not explored or discussed.

Reply: We thank the reviewer for urging us to evaluate alternative mechanisms, which could help strengthen the conclusion of our work. To address this question, we examined several types of alternative models for the hybrid learning framework.

First, as described above, we compared the hybrid learning model with single-strategy models and found that the hybrid learning model outperformed the single-strategy models (Fig. R5 and Table R1).

Second, we considered alternative models for the singleton condition where people were first trained with one-cue trials and then switched to three-cue training trials. One possibility is that participants ceased learning during three-cue trials after initial training with single cues. To test this, we compared models that either allowed or disallowed learning in multi-cue trials. We found that the model incorporating multi-cue learning provided a better fit to participants' choices than the model without multi-cue learning (Fig. R9a), ruling out this explanation. Another possibility is that behavior in the singleton condition could be better captured by marginal-only or joint-only models if they were allowed to use different learning rates for one-cue and three-cue trials. Although this adjustment improved the fit of the single-strategy models, they still performed worse than the hybrid model (Fig. R9b).

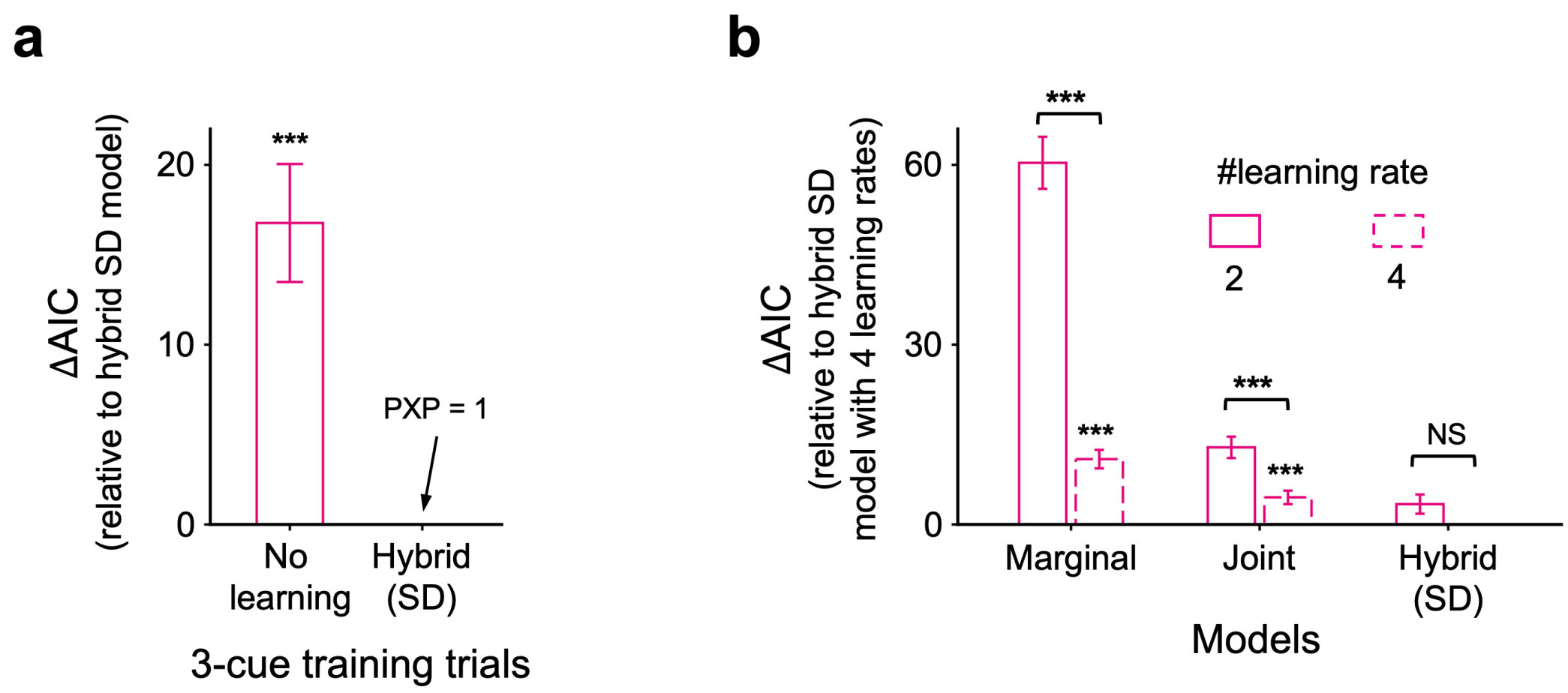

Fig. R9. Alternative models in singleton condition. The observed behavior could not be better explained by participants ceasing to learn during three-cue trials (a), nor by assuming different learning rates for single- versus multi-cue trials (b).

Third, we tested another set of alternatives focusing on the dynamics of the hybrid SD model, which showed an increasing proportion of joint updating trials over the course of training (Fig. R7a). One possibility is that participants simply increased the weight of joint updating as a function of the trial number (i.e., $\gamma = \frac{1}{1+e^{-b_1 \cdot t + b_0}}$, t is the trial number, b_1 and b_0 are two free parameters). Another is that the learning strategy was determined by whether a trial was rewarded or not (i.e., joint updating in rewarded trials and marginal updating in unrewarded trials), which could also yield an increasing proportion of joint updating as performance improved during training. Model fits showed that neither of these accounts outperformed the hybrid SD model (Fig. R10a). We also considered whether only the overall proportion of

joint updating mattered, rather than the specific mapping of different strategies to trials. To test this, we preserved the trial-by-trial proportions of joint updating inferred from the hybrid SD model but randomly shuffled their assignment to trials within small temporal windows (sizes = 1, 2, 5, or 10 trials; with size = 1 corresponding to the original model). After each shuffling, we estimated the other parameters in the model (i.e., α_+, α_-, and β_1), but updated the weight based on the assigned learning strategy. If only the overall proportion mattered, shuffling should not reduce model fit. Instead, even minimal shuffling (window size = 2) impaired model performance, indicating that the trial-specific assignment of learning strategies is critical (Fig. R10b). These results suggest that participants adaptively choose updating strategies on a trial-by-trial basis, as captured by the hybrid SD model.

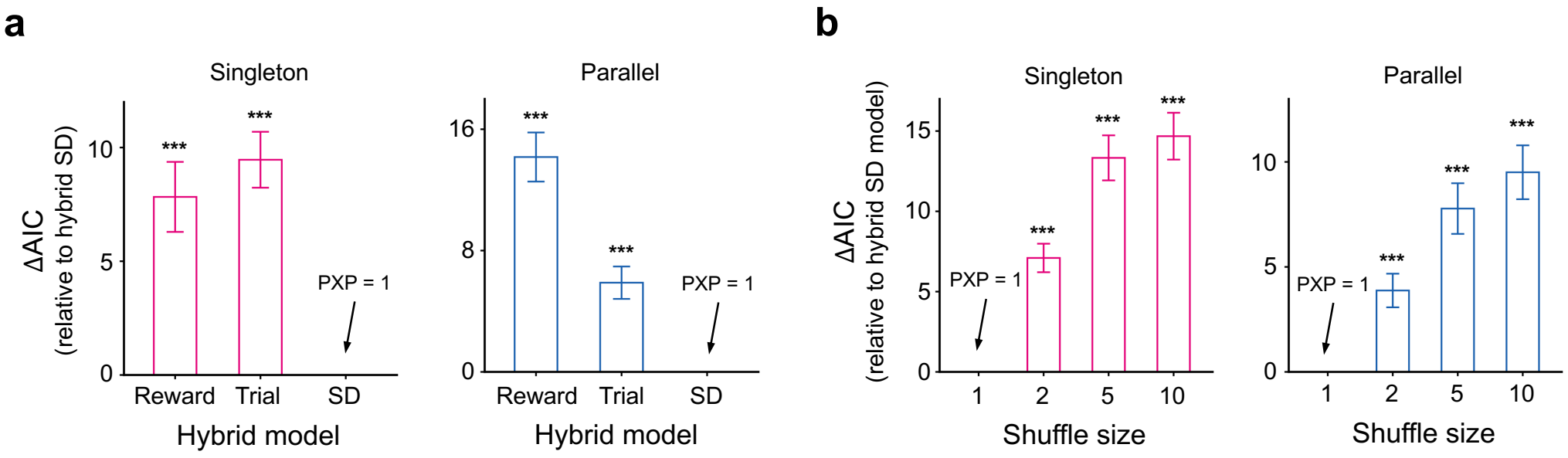

Fig. R10. Alternative explanations for the hybrid SD model. (a) In both conditions, models that select strategies solely by trial index or by reward outcome perform worse than the hybrid SD model in capturing the observed behavior. (b) Randomly shuffling the trial-by-trial assignment of learning strategies impairs model performance, indicating that the specific mapping of strategies to trials is critical.

Forth, we asked whether the observed behavior could be explained by changes in learning rate over time, rather than by a hybrid learning mechanism. To test this, we added a decay parameter that allowed learning rates to decrease across trials. Specifically, the learning rate at trial t was computed as $\alpha_t = \alpha_0 \cdot e^{-\eta \cdot t}$, where α_0 is a free parameter representing the initial learning rate, η is a free parameter capturing the rate of decay and the trial index is denoted by t, starting from 0. Incorporating learning rate decay improved the fit of all models in both conditions (Fig. R11a), suggesting that participants reduced their learning speed as training progressed, possibly due to cognitive fatigue. However, the hybrid SD model continued to outperform both the marginal-only and joint-only models (Fig. R11a), demonstrating that a mixed strategy provides a better account of the learning behavior. Importantly, the value of the decay parameter was comparable across conditions (Fig. R11b), suggesting similar learning motivation across groups.

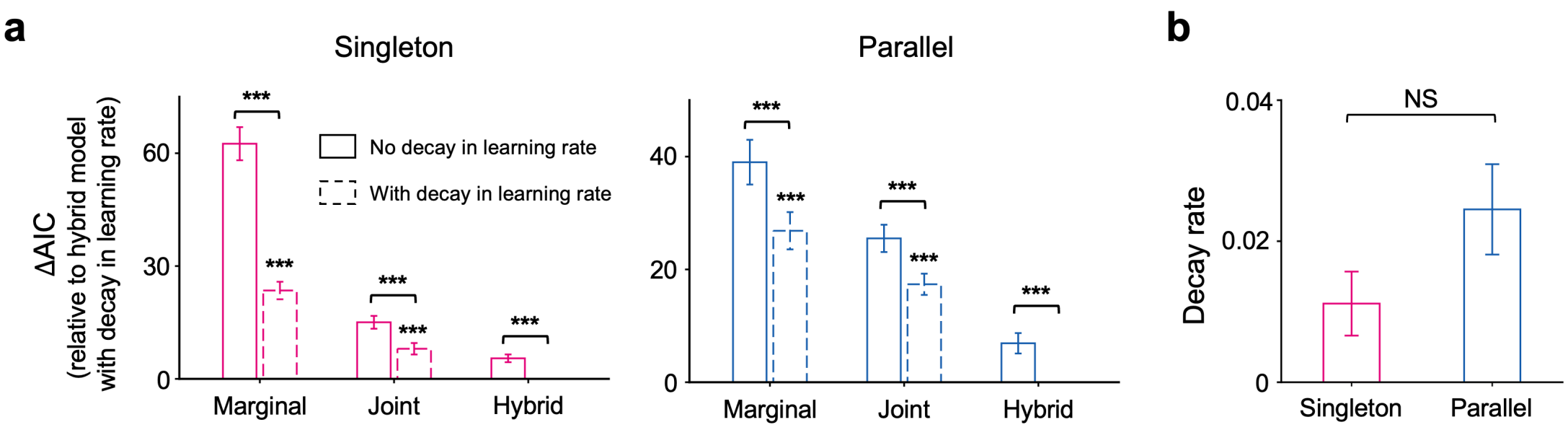

Fig. R11. Models with decay in learning rates. (a) Adding a decay term in learning rate improves overall fit, yet the hybrid model continues to outperform single-strategy models. (b) The estimated decay parameter does not differ between the singleton and parallel conditions.

Finally, we considered the role of selective attention. Previous studies have shown that learners assign different attentional weights to different features in multi-feature tasks (Leong et al., 2017; Wang & Soltani, 2025). To test whether this applies in our cue combination task, we extended each model by adding an attentional weight parameter to each cue, applied during both learning and decision stages (see Methods for details). Including attentional weights improved model fits, but the hybrid SD model remained the best-fitting account overall (Fig. R12a). Moreover, the estimated attentional weights showed an inverted U-shaped relationship with the objective WOE values: cues with more extreme WOE values were assigned less weight (Fig. R12b). This pattern is consistent with the "robust averaging" effect, where learners down-weight extreme information to avoid being misled (De Gardelle & Summerfield, 2011; Li et al., 2017).

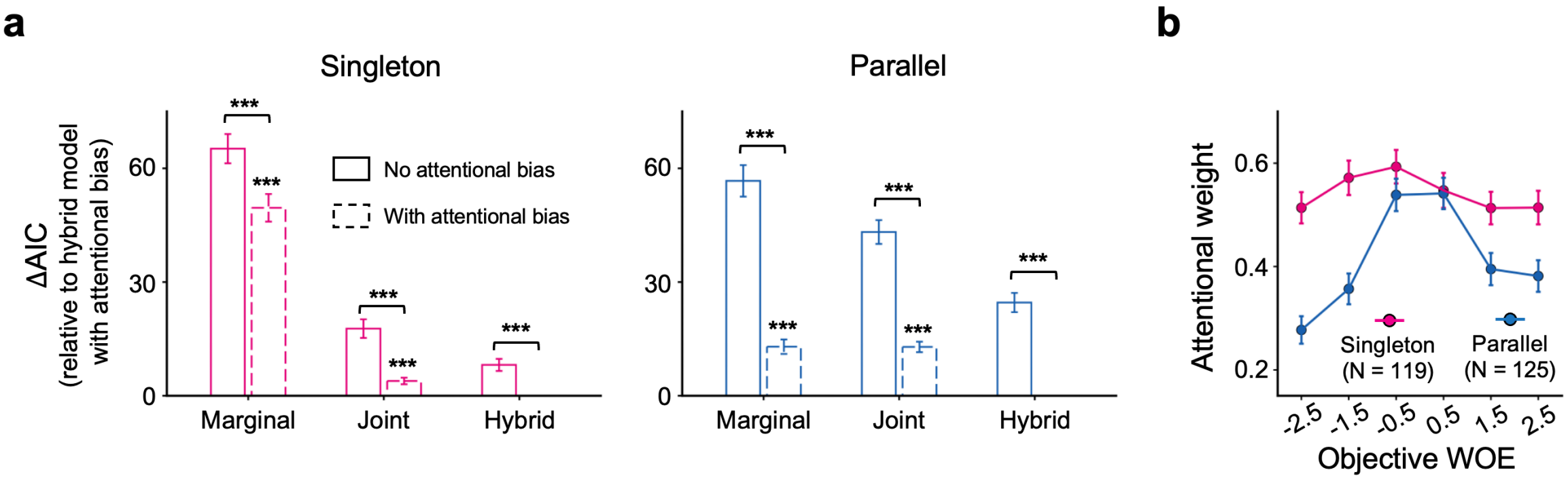

Fig. R12. Models with attentional weights. (a) Adding cue-specific attentional weights improves model fit, while the hybrid model still outperforms single-strategy alternatives. (b) Estimated attentional weight for each cue.

Together, the above results provide strong evidence for a hybrid learning framework. We have incorporated the model-comparison results into the manuscript (revisions in L12P12–L20P12; see also Figs. S12–14) and expanded the Discussion accordingly (revisions in L28 P17–L36 P17).

- **Leong, Y. C., Radulescu, A., Daniel, R., DeWoskin, V. & Niv, Y. Dynamic Interaction between Reinforcement Learning and Attention in Multidimensional Environments. Neuron 93, 451–463 (2017).**
- **Wang, M. C. & Soltani, A. Contributions of Attention to Learning in Multidimensional Reward Environments. J. Neurosci. 45, e2300232024 (2025).**
- **De Gardelle, V. & Summerfield, C. Robust averaging during perceptual judgment. Proc. Natl. Acad. Sci. U.S.A. 108, 13341–13346 (2011).**
- **Li, V., Herce Castañón, S., Solomon, J. A., Vandormael, H. & Summerfield, C. Robust averaging protects decisions from noise in neural computations. PLoS Comput Biol 13, e1005723 (2017).**

3. The controls performed in experiments 2 and 3 are of limited value. For experiment 2, it is perfectly possible that motivation might be reset by being offered a novel opportunity with new stimuli to learn about – it is well known that someone might give up on a "too hard" context, but try again in a new context. L34 p4 is not a satisfactory interpretation.

Reply: The reviewer is right that our design cannot rule out the possibility that both motivated and de-motivated participants "reset" to the same level of motivation in the trailing task (although we think this is rather unlikely, given that motivation in sequential tasks tends to be at least partly correlated). To accommodate this point, we have added a

caveat to the point that was originally on L34P4, which raises the alternative explanation suggested by the reviewer (revisions in L24P6–L26P6).

However, we would also draw the reviewer's attention to three additional results that offer circumstantial evidence against motivational differences as the source of the singleton effect.

- **First, RTs during testing did not differ between singleton and parallel conditions (Exp. 1–3: β = -0.02, *SE* = 0.042, *t(241.4)* = 0.52, *P* = 0.603). If participants in the parallel condition had been less motivated, they would likely have responded faster to complete the task more quickly.**
- **Second, as suggested by Reviewer 3, we examined whether performance differences between groups changed over time, particularly during the testing phase. If participants in parallel condition were less motivated, the performance differences between groups should become larger and larger as the testing progressed, suggesting a significant internation between trial number and condition in predicting the choice accuracy. However, no such an interaction was observed ($\beta_{trial \times condition}$ = 0.0002, *SE* = 0.00029, *z* = 0.70, *P* = 0.487), implying a comparable motivation during testing.**
- **Thirdly, computational modelling that allowed the learning rate to decay with training trials revealed no differences in the decay parameter between conditions (Fig. R11b; *t(221.2)* = -1.7, *P* = 0.09). If motivation was lower in the parallel condition, we would expect a stronger decay.**

Together, these results indicate that motivational differences are unlikely to account for the singleton effect.

(Reviewer comment continued) For experiment 3, half of the total combination of cues is still 10 combinations, which is still way above working memory capacity/resource limitations, so is again not a good control for the proposed rationale of ruling out memory effects.

We think that there may be some confusion here, no doubt due to a lack of clarity on our part. Exp. 3 was designed to test whether the singleton effect generalizes beyond specific training combinations. Therefore, participants were trained on half of the possible three-cue combinations and then tested on all. The results showed that singleton training improved accuracy not only for trained combinations (β = 0.32, *SE* = 0.158, *z* = 1.99, *P* = 0.023, one-tailed), but also for novel, untrained ones (β = 0.34, *SE* = 0.144, *z* = 2.39, *P* = 0.009, one-tailed). This result demonstrates that the effect generalizes beyond the specific experiences provided during training. This logic holds independent of the number of cue combinations that were trained. We have added additional details to our interpretation of this finding to make sure it is clear.

In summary, we have clarified the aims of Exp. 2 and 3 and added new evidence to show that the singleton effect is unlikely to arise from motivational differences. We have revised the manuscript to make these points explicit (revisions in L1P6–L36P6) and reordered Exp. 2 and 3 to present the results more clearly.

4. The results of the last experiment (fig. S4) are described as matching the predictions of the model, but this is not fully the case: ascending and descending do not differ from singleton, whereas the model predicts a difference. The other prediction of the model is that there should be no difference

between the ascending and descending conditions, and this prediction is borne, but this is a null effect in a highly variable sample, and no test of this null hypothesis is given.

Reply: We thank the reviewer for the careful comparison and pointing out the gap between our initial model predictions and the empirical results in Exp. 6. Fortunately, after revising our modelling framework, the out-of-sample model predictions closely match the observed results.

In particular, the hybrid SD model using the parameter values estimated from Exp. 1-3 predicts that participants under ascending and descending training should have a similar learning performance, which is comparable to the singleton condition and superior to the parallel condition (Fig. R13a). The observed results are now quite consistent with the model predictions (Fig. R13b).

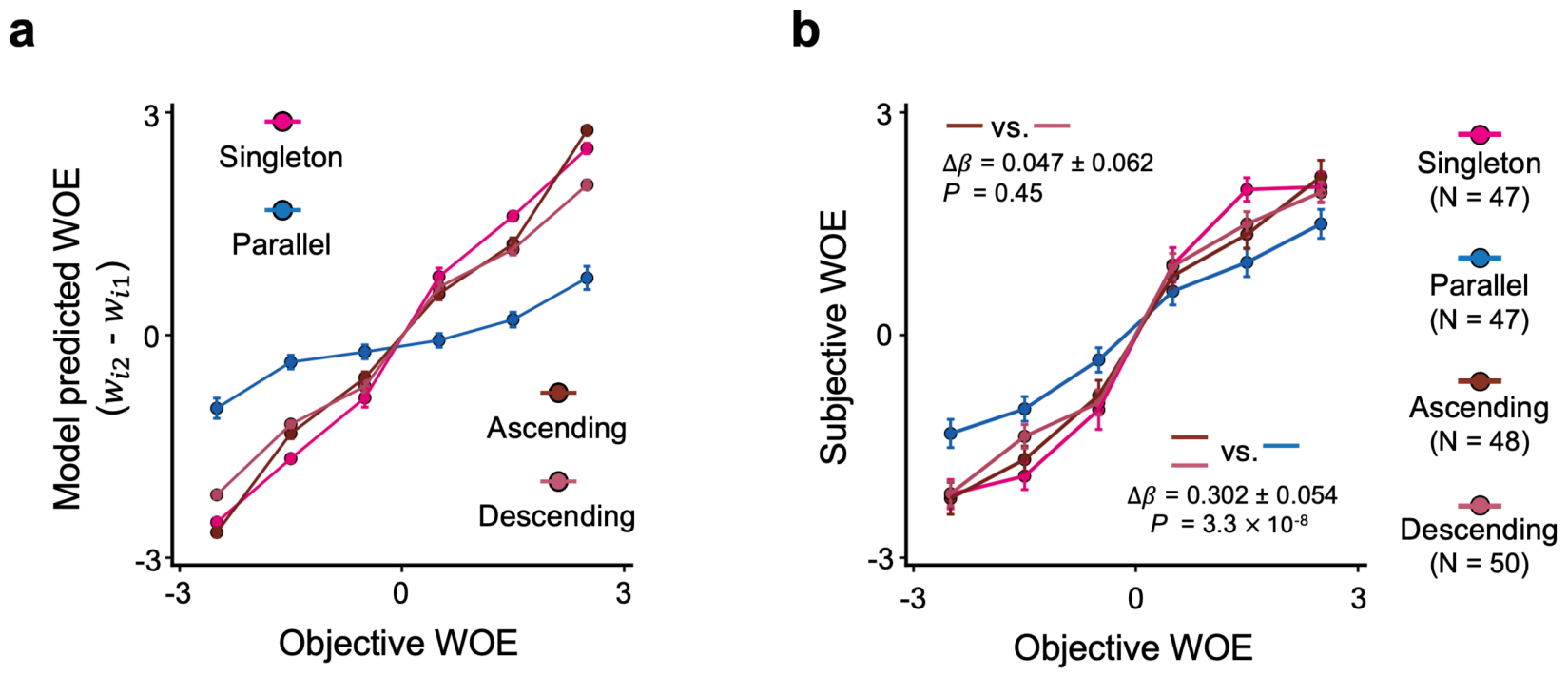

Fig. R13. Model predicted (a) and empirically observed (b) results in Exp. 6. The out-of-sample predictions by the hybrid SD model (a) show similar patterns to the empirical results.

We have now revised our manuscript accordingly (revisions in L14P16–L23P16; see also Figs. S17b–c).

5. The exclusion rate is not reported (only final number of participants included, and exclusion criteria; missing number of participants tested).

Reply: Thanks for this comment. Exclusion rate was previously included in the reporting summary. We have now revised the manuscript to add more detailed information about our participants, including their age and gender, as well as the number of participants tested and excluded.

Across experiments, exclusion rate ranges from 8.8% to 22.5%. These differences likely reflect variations in experiment length and the timing of data collection (e.g., weekday vs. weekend, day vs. night). Importantly, these rates are in line with those reported in other online behavioral studies (e.g., Garcia et al., 2023; Findling & Wyart, 2024; Hayes & Wedell, 2023). The updated participant information is now included in the Methods section (revisions in L9P23–L18P23 for Exp. 1, L14P27–L16P27 for Exp. 2, L15P28–L17P28 for Exp. 3, L29P28–L31P28 for Exp. 4, L9P29–L11P29 for Exp. 5, and L28P29–L31P29 for Exp. 6).

- **Garcia, B., Lebreton, M., Bourgeois-Gironde, S. & Palminteri, S. Experiential values are underweighted in decisions involving symbolic options. Nat. Hum. Behav. 7, 611–626 (2023)**
- **Findling, C. & Wyart, V. Computation noise promotes zero-shot adaptation to uncertainty during decision-making in artificial neural networks. Sci. Adv. 10, eadl3931 (2024).**

- **Hayes, W. M. & Wedell, D. H. Effects of blocked versus interleaved training on relative value learning. Psychon. Bull. Rev. 30, 1895–1907 (2023).**

6. It is unclear whether the broader conclusion about specific curricula, or the use of the theoretical framework, has any chance of translating to other learning situations, in particular in light of the lack of validity of the theoretical framework as a learning mechanism.

   Reply: Knowing how well findings generalize to real-world learning situations is a challenge for all laboratory-based work in psychology. We have now made this caveat explicit in the discussion (revisions in L5P17–L6P17). The cue combination task has been exceptionally widely used in psychology because it simulates many real-world problems where several discrete and independence pieces of evidence must be combined, such as medical, legal or financial decision-making. Nevertheless, we agree that it would be highly desirable to show that the proposed "divide and conquer" applies for a different task. Thus, we have now tested whether our findings are specific to the current task, by reporting the results of a new experiment in revised manuscript.

   Rather than simply changing the surface characteristics of the Weather Prediction Task, we used an entirely new (and more complex) task called "Pointer". Participants learned to predict the final location of an avatar on linear track given a random starting location and a sequence of symbols (Fig. R14a). The track was divided into 21 discrete numbered locations (coordinates -10 to 10). Each symbol denoted a simple arithmetic operation that moves the avatar along the track (e.g., the symbol denoting "+1" moves the avatar from location n to location $n+1$; Fig. R14b). Operations were applied sequentially—the output of one symbol served as the input for the next.

   This design allowed to once again compare a singleton condition (where participants learn one symbol at a time) to a parallel condition (three symbols per training trial; Figs. R14c-d). As expected, the singleton group outperformed the parallel group on identical three-symbol test trials (β = 1.77, *SE* = 0.379, *z* = 4.67, $P < 0.001$; Fig. R14e). These results indicate that the singleton advantage generalizes to a new and different learning task.

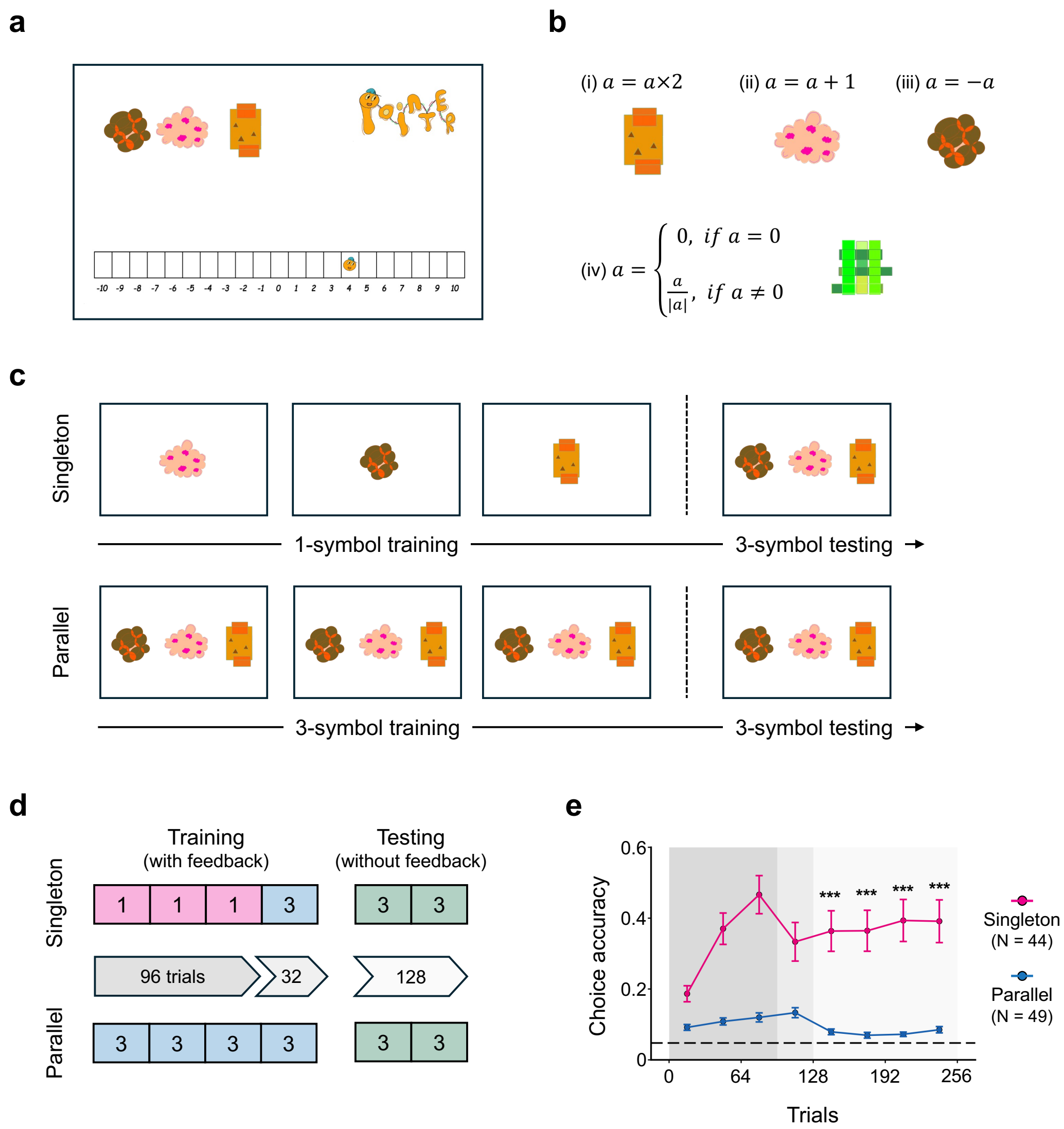

Fig. R14. Generalization of the singleton effect to another compositional task. (a) In pointer game, participants reported the final position of an avatar on a one-dimensional track, given a starting position and a sequence of "magic" symbols which denoted arithmetic operations that could change the location of the avatar. For a sequence of symbols, the output of the first symbol served as the input to the next. In this example, the final position of the avatar is -6 (intermediate position: -4, then -3, then -6 after the first, the second and the third symbol, respectively). (b) Each symbol denoted a simple mathematical operation. For each participant, four symbols were sampled from a set of 12; symbol–operation mappings were randomized across participants. (c) Illustration for the curricula. In the singleton condition, participants were trained with trials containing a single symbol during the initial training, whereas in the parallel condition, training involved trials with three symbols. Test trials, which included three symbols, were the same across conditions. (d) Training procedure for singleton and parallel condition. The two conditions differed only in the initial training phase. Numbers in cells indicate the number of symbols shown per trial. (e) Mean choice accuracy across training and testing for each condition. Each point reflects accuracy averaged over 32 consecutive trials per participant and then across participants within a condition. Error bars denote the SEM. Light grey, grey, and dark grey shaded areas indicate different task stages in (d).

We recognize that generalization of this effect to one other task does not mean that it will generalize to all other tasks. No one study can test the full generalizability of a new psychological finding; this is the job of the entire research community. We also realize that the benefit of the 'divide and conquer' strategy may be reduced, particularly when subcomponents interact in non-additive or context-dependent ways (Franklin & Frank, 2020; Lake et al., 2017). In such cases, additional mechanisms—such as joint representations, higher-order rules, or explicit feedback—may be required to capture dependencies among

components. Nevertheless, this additional study shows that our finding is not unique to the Weather Prediction Task.

Regarding the theoretical interpretation for the observed singleton effect, we developed a hybrid learning framework and validated it rigorously. We (i) fitted trial-by-trial choices and performed formal model comparison, (ii) conducted model and parameter recovery to establish identifiability, and (iii) tested the model's out-of-sample predictions, which matched new data. This framework not only aligns with prior evidence for hybrid learning mechanisms across many domains, from non-social reinforcement learning to strategic decision making (Charpentier et al., 2020; Daw et al., 2011; Zhu et al; 2012), but also extends it by allowing strategy weights to adapt to learning contexts and accumulated knowledge. From a resource-rational perspective, such an arbitration in learning strategies reflects a principled trade-off between learning precision and cognitive cost under resource constraints. We believe these new results should increase our confidence about the validity of the proposed theoretical framework and its potential to be extended to other learning contexts.

We have incorporated the results of the new compositional task into the revised manuscript (revisions in L4P8–L14P8; see also Fig. S5) and expanded our discussion to reflect the above points, focusing on the divide-and-conquer strategy, the hybrid learning framework, and the broader implications for curriculum design (revisions in L8P17–L18P18).

- **Franklin, N. T. & Frank, M. J. Generalizing to generalize: Humans flexibly switch between compositional and conjunctive structures during reinforcement learning. PLoS Comput. Biol. 16, e1007720- (2020).**
- **Lake, B. M., Ullman, T. D., Tenenbaum, J. B. & Gershman, S. J. Building machines that learn and think like people. Behav. Brain Sci. 40, e253 (2017).**
- **Charpentier, C. J., Iigaya, K. & O'Doherty, J. P. A Neuro-computational Account of Arbitration between Choice Imitation and Goal Emulation during Human Observational Learning. Neuron 1–21 (2020) doi:10.1016/j.neuron.2020.02.028.**
- **Daw, N. D., Gershman, S. J., Ben Seymour, Dayan, P. & Dolan, R. J. Model-Based Influences on Humans' Choices and Striatal Prediction Errors. Neuron 69, 1204–1215 (2011).**
- **Zhu, L., Mathewson, K. E. & Hsu, M. Dissociable neural representations of reinforcement and belief prediction errors underlie strategic learning. Proc. Natl. Acad. Sci. U.S.A. 109, 1419–1424 (2012).**

7. No minor issues. Thanks for a well edited paper.

Reply: We thank the reviewer for the positive words!

Reviewer #3:

0. Summary

The study investigates how different training curricula affect human learning in a probabilistic cue combination task. Across six behavioral experiments, participants learn to predict weather outcomes based on up to three cues presented on every trial, with each cue carrying a predictive value. Participants are assigned to different curriculum conditions: one group (singleton) is exposed to single cues during training, while another group (parallel) learns with three cues presented simultaneously. Within the parallel condition, the study further varies the training curricula by manipulating the distribution of summed predictive values of cues, the progression in the number of cues per block (increasing or decreasing), and the nature of feedback (deterministic vs. stochastic). Results show that participants in the singleton group learn cue-outcome associations more accurately and rapidly, and generalize better to novel three-cue combinations. To explain these findings, the authors propose a boundedly rational model that assigned credit independently to single cues, implementing the assumption that participants can only focus on one cue at a time. Using this model, they identify optimized training curricula for the parallel group that enhance learning outcomes and find that such a curriculum indeed improves learning performance comparable to that of the singleton group. The study concludes that humans benefit from curricula that support a divide-and-conquer learning strategy, with performance gains emerging from cognitively bounded mechanisms for credit assignment.

Strengths

The study has several strengths that make it a valuable contribution to the literature. First, it tackles a timely and important question about the cognitive mechanisms that support—and are supported by—different learning curricula in humans. This issue is one that nearly every experimental psychologist confronts in practice when designing training procedures for behavioral tasks, yet mechanistic and formal insights into why certain curricula are more effective than others remain scarce. This study approaches this question through a series of experiments that are simple and cleverly designed to test the underlying hypothesis that humans benefit from a divide-and-conquer strategy when learning to predict outcomes based on probabilistic cues.

Perhaps the most impressive aspect of the study is its use of a computational model not only to explain observed behavioral patterns but also to derive strong, testable predictions about how to optimize training curricula—in this case, for the parallel learning condition. The authors show that, a highly skewed distribution of summed cue values, as identified with the model, can improve generalization performance in the parallel curriculum. By empirically confirming these predictions, the study demonstrates how computational insights into curriculum learning can be used to design better training strategies– potentially with applications beyond the lab. Additionally, the model's predictions are further validated by experiments showing that manipulating task difficulty over time (e.g., increasing or decreasing the number of cues presented on a trial) does not benefit learning in this task—highlighting that not all curriculum structures are equally beneficial.

Finally, the manuscript is well written, the figures are informative, and the presentation appears accessible to a broad scientific audience.

Weaknesses

The primary weaknesses of the manuscript concern the modeling approach and its limitations in supporting strong inferences about the underlying cognitive mechanisms. While the study introduces a compelling computational model, its evaluation relies on simulations trained to convergence and comparisons to summary-level behavioral outcomes, rather than formal model fitting to trial-by-trial behavioral data. This limits the ability to dissociate competing explanations and fails to leverage the rich within-subject dynamics present in the data. In particular, the modeling framework would benefit from fitting free parameters (e.g., learning rate, decision noise) to participants' actual choices, allowing for more rigorous model comparisons (Major Comment 1). The assumption that participants reach asymptotic performance (when they do not) may obscure key dynamics, such as differences in learning rates across curricula, and prevents the model from capturing meaningful variation during training and testing (Major Comments 1a and 1b). Moreover, the manuscript could be significantly improved by formally specifying its core mechanism of bounded credit assignment while distinguishing it from alternative explanations (Major Comment 1c).

In addition to these modeling concerns, there are some more minor issues related to the statistical treatment of WOE estimates (e.g., reliance on point estimates in t-tests without accounting for estimation uncertainty; see Comment 2), the need to clarify cue presentation frequencies across experiments (Comment 3), and the unaddressed possibility that motivational differences may interact with fatigue in Experiment 2 (Comment 4).

While these concerns—elaborated below—should not detract from the article's substantial strengths, addressing them could further strengthen an already impressive study.

Reply: We thank the reviewer for the positive words and thoughtful feedback. Addressing these comments, especially the concern related to our modelling framework, greatly helps us strengthen and clarify the manuscript. Below, we briefly summarize how we have addressed the reviewer's main concerns (with detailed responses provided under each specific question).

First, to address the limitations of convergence-based simulations, we rebuilt the modelling framework to fit trial-by-trial choices and capture individual differences (Question 1). Specifically, we (i) introduced a hybrid learning model that arbitrates between the two learning strategies described in the original submission (Figs. R1–4), (ii) performed formal model estimation and comparison to identify the best-fitting account of participants' trial-by-trial choices (Figs. R5–7; Table R1), (iii) conducted model and parameter recovery to establish identifiability and reliability (Fig. R8), (iv) tested and ruled out a series of alternative mechanisms (Figs. R9–12), and (v) examined individual differences (Figs. R7 and R15). The revised framework reproduces observed learning trajectories and outperforms single-strategy alternatives, substantially strengthening our modelling approach.

Second, we addressed the statistical and design concerns (Question 2). To mitigate potential Type I error inflation, we re-analysed WOE estimates using reliability-adjusted WOE values; the conclusions were unchanged (Fig. R20). We also clarified that cue presentation frequencies were matched across experiments to avoid confounds (Question 3). In addition, we added analyses to probe motivation (including time-course and modelling checks), finding no evidence that motivational differences account for the singleton advantage (Question 4). Finally, we addressed the remaining minor points (Questions 5–7).

We greatly appreciate the reviewer's insightful and constructive comments, and believe these revisions guided by the reviewer's suggestions have significantly improved the manuscript's clarity and rigor. We thank the reviewer again and would be glad to address any further questions.

Major Comments

1. Performing model fits to better dissociate alternative hypotheses. One of the study's strengths is its use of a computational model to formalize hypotheses about cognitive mechanisms underlying curriculum learning and to generate predictions for improved curricula that are then tested empirically. However, the current approach to model evaluation—based on summary statistics—limits the ability to differentiate between competing mechanistic explanations of the observed behavior.

   The study would benefit from fitting the models directly to participants' trial-by-trial choice data using likelihood-based methods, which is standard practice in modeling learning behavior (e.g., especially reinforcement learning models). Instead of training the model to convergence, this would involve fitting free parameters (e.g., learning rate, decision noise) to each participant's choice series and comparing models based on fit metrics that also account for model complexity (e.g., AIC, BIC, or cross-validated likelihood). Such an approach would provide a more rigorous test of the model and allow the authors to evaluate individual by individual which model fits better, potentially exposing relevant individual differences. In addition, such an approach would address several related concerns:

   Reply: We thank the reviewer for the excellent suggestions for (i) performing model fitting and (ii) exploring individual differences. The first aspect of this comment regarding the model fitting was also raised by Reviewer 1, we thus address these related concerns together in our response to Question 1 by Reviewer 1. Here we only provide a brief summary of the revisions we have made (please see our reply to Question 1 by Reviewer 1 for details).

   Per the reviewer's suggestion, we substantially revised our modelling framework and performed formal model estimation and comparison. First, we introduced a hybrid learning framework which combines marginal updating with joint updating, corresponding to the boundedly rational learner and optimal learner in our initial submission (Figs. R1–4). This model allows participants to flexibly arbitrate between a marginal and a joint updating strategy depending on the trade-off between their relative learning precision and relative cognitive cost, consistent with a resource-rational account (Bhui et al., 2021; Gershman et al., 2015; Goodman & Frank, 2016; Lieder & Griffiths; 2020). Second, we fit all competing models with participants' trial-by-trial choices and found that the hybrid learning model (i.e., the hybrid SD model) explained participants' choices better than single-strategy models (Fig. R5; Table R1). Third, we performed both model and parameter recovery, confirming that the hybrid learning model is distinguishable from alternatives and that its parameters can be reliably estimated (Fig. R8).

   The second aspect of this comment is related to the participants' individual differences in the task. To address this point, we associated the estimated model parameters with participants' learning performance. The hybrid learning model contains four free parameters (Table R1): two learning rates for positive and negative outcomes (α_+ and α_-), one parameter for the cost in evidence integration from one cue to multiple cues (β_1), and one parameter for the relative cognitive cost of joint versus marginal updating (θ). We run a

regression analysis by using the estimated model parameters together with a condition indicator to predict participants' choice accuracy in the test trials (Fig. R15a). We found that α_+, β_1 and θ were all significantly correlated with participants' choice accuracy (see Figs. R15b–d for illustration of the regression results in Fig. R15a), indicating that better learning performance was associated with greater positive learning rate (α_+), lower integration noise (β_1), and lower cognitive cost for joint updating (θ).

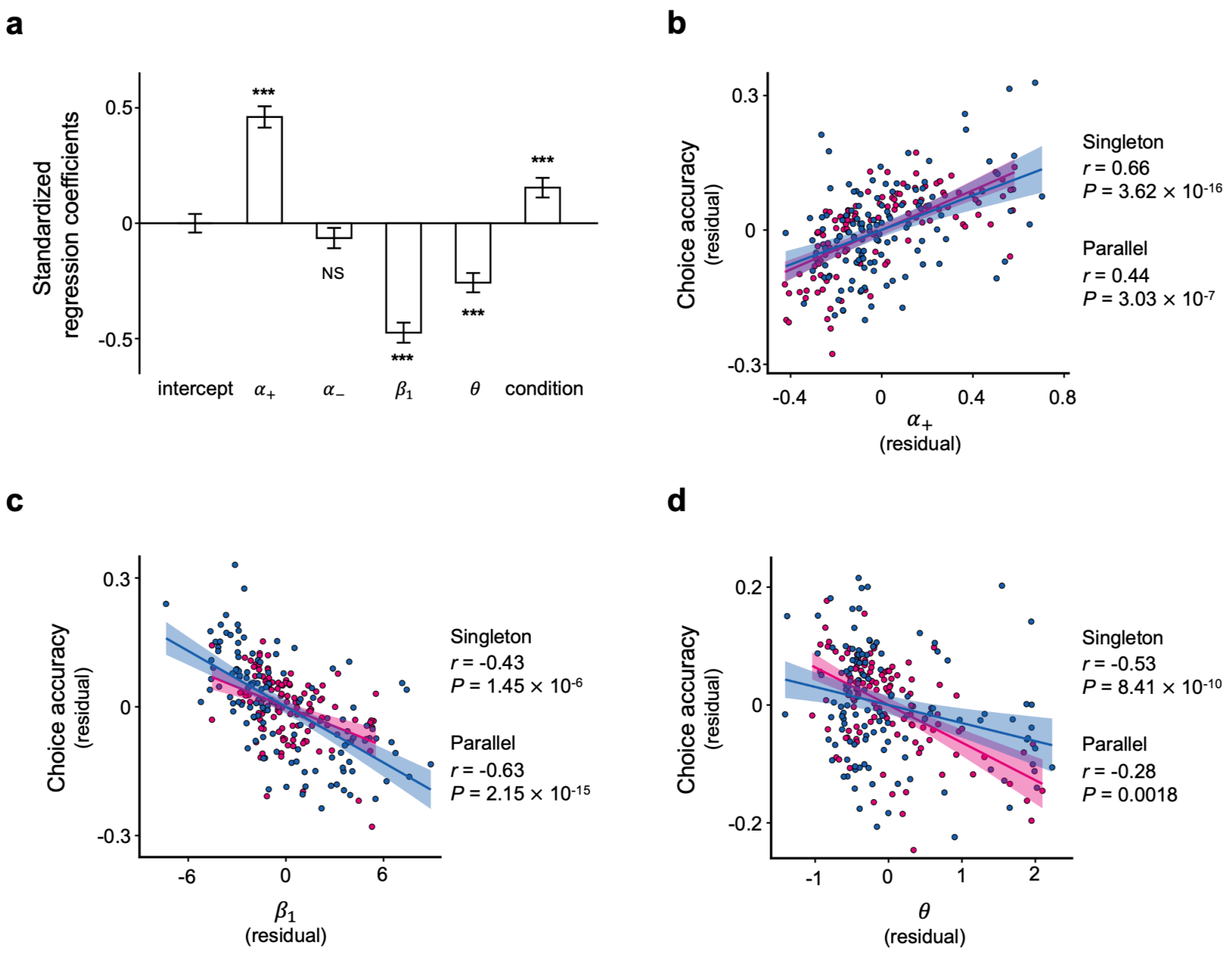

Fig. R15. Individual differences. (a) Regression analysis using estimated parameters and training condition to predict choice accuracy in test trials. (b-d) Relationships between choice accuracy and individual parameters (α_+, β_1 and θ), shown for illustration of the regression results.

Interestingly, in Fig. R15a, after controlling for the model parameters between singleton and parallel conditions, we still observed an effect of training conditions. This result suggests that the observed singleton effect is not caused by the parameter differences between conditions. We then asked what may drive the learning advantage of singleton training. Further analyses revealed that participants' reliance on joint updating may play a role in the singleton effect (Fig. R7). In both conditions, participants who had a larger proportion of multi-cue trials using joint updating performed better in the task (Fig. R7b). More importantly, when we included both the condition indicator and the proportion of joint updating in a single regression to predict choice accuracy, the condition effect disappeared (singleton vs parallel: β = -0.004, *SE* = 0.0129, *t* = -0.31, *P* = 0.755; proportion of joint updating: β = 0.270, *SE* = 0.0182, *t* = 14.82, *P* < 0.001). These results thus suggest that the observed singleton effect might be explained by differences in the reliance on joint updating across conditions, which in turn are shaped by the training regimes.

Taken together, these results provide a rigorous evaluation of our modeling framework and strengthen our theoretical claims. Beyond the revisions detailed in our response to Question 1 by Reviewer 1, we have updated the manuscript to report individual-differences analyses (revisions from line 25 page 11 to line 10 page 12 (L25P11–L10P12); see Figs. 3g–h and Figs. S9e–h).

- **Bhui, R., Lai, L. & Gershman, S. J. Resource-rational decision making. Curr. Opin. Behav. Sci. 41, 15–21 (2021).**
- **Gershman, S. J., Horvitz, E. J. & Tenenbaum, J. B. Computational rationality: A converging paradigm for intelligence in brains, minds, and machines. Science 349, 273–278 (2015).**
- **Goodman, N. D. & Frank, M. C. Pragmatic language interpretation as probabilistic inference. Trends Cogn. Sci. 20, 818–829 (2016).**
- **Lieder, F. & Griffiths, T. L. Resource-rational analysis: Understanding human cognition as the optimal use of limited computational resources. Behav. Brain Sci. 43, e1-85 (2020).**

1a) The current model simulations assume convergence, which does not seem to be supported by the empirical data. In particular, performance in the parallel group does not appear to saturate during training (e.g., see Figure 2E). This matters because, under convergence assumptions, differences in learning rate between models have no effect. However, an alternative explanation for the observed performance gap between the singleton and parallel groups could be that participants in the parallel condition learn more slowly—perhaps because multi-cue trials increase cognitive load and reduce effective learning rates. While this hypothesis may ultimately be incorrect, it is important to rule it out. Fitting learning rates for both groups would allow to test whether performance differences can be accounted for by slower learning alone, or whether curriculum structure provides an additional benefit. Alternatively, the authors could demonstrate within their current simulation framework that varying the learning rate in the optimal learner does not yield benefits from highly skewed cue distributions unless the boundedly rational mechanisms are in place (but see Major Comment 1c).

Reply: We thank the reviewer for the thoughtful comments and helpful suggestions. As suggested by the reviewer, we relaxed the convergence assumption in our original simulations by performing model estimation without requiring learning to be fully converged.

Consistent with the reviewer's intuition, participants in the singleton condition showed a higher positive learning rate than those in the parallel condition (Fig. R16 and Table R1), likely reflecting the reduced credit-assignment demands of single-cue compared to multi-cue training. However, the parameter differences alone cannot explain the singleton advantage. The effect remained even when we controlled for the parameter differences in a regression analysis which included both estimated parameters and a condition indicator to predict learning performance (Fig. R15a; see also our reply to the above question).

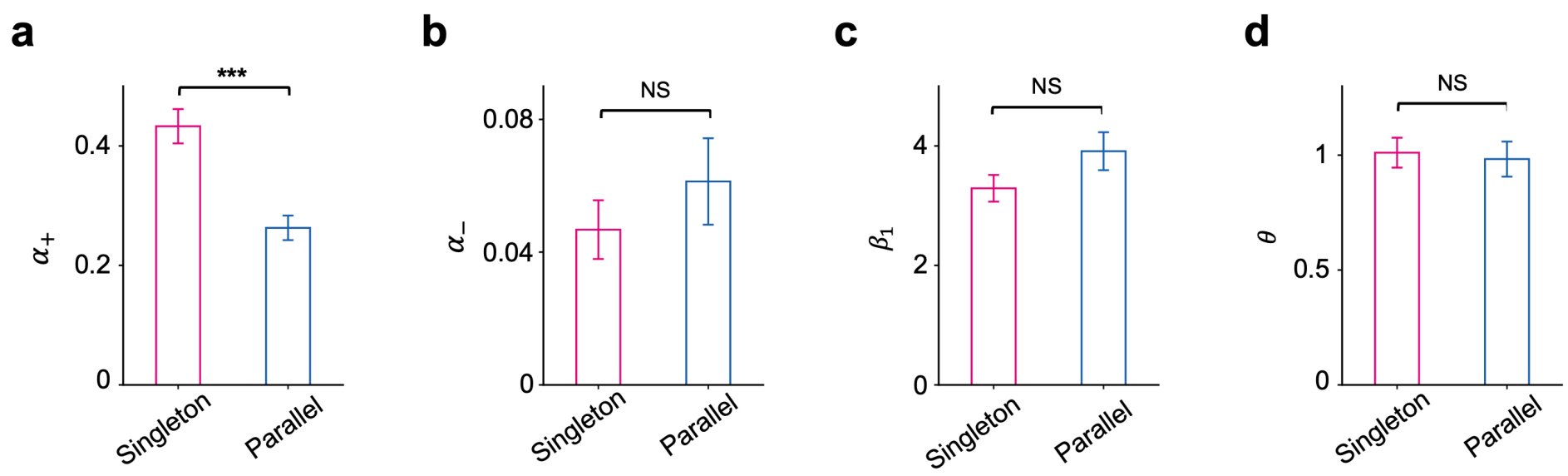

Fig. R16. Estimated parameter values by the hybrid learning model. Participants trained under the singleton condition exhibited a higher positive learning rate (α_+) compared to those in the parallel condition, while the other parameters (α_-, β_1 and θ) were comparable across conditions.

This is further confirmed by a simulation analysis using identical parameter values to simulate participants' learning performance in both conditions (Fig. R17). Specifically, we adopted the median of each estimated parameter values, derived from the hybrid learning model in the parallel condition, for simulations. The results showed that participants under

singleton training robustly generated better performance even with matched parameters with parallel condition (Fig. R17a). This effect was unique to the hybrid learning model: when simulations were performed using only marginal or joint updating models with the same parameters, the singleton advantage disappeared (Fig. R17b).

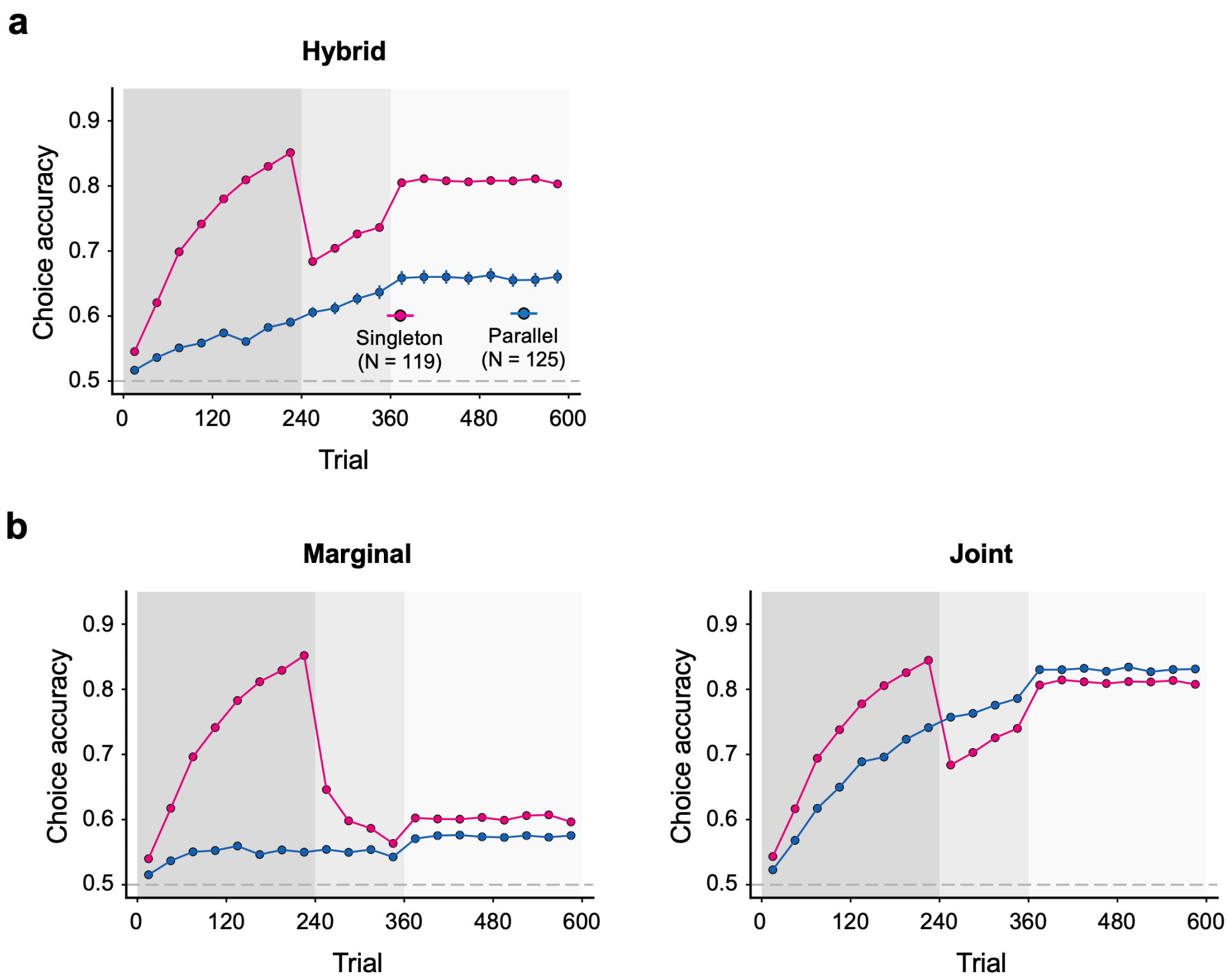

Fig. R17. Simulation results using the same parameter values across conditions. (a) The hybrid learning model robustly reproduced the singleton learning advantage even with matched parameters between singleton and parallel conditions. The simulations were performed using the median of the parameter estimates derived from the hybrid learning model in the parallel condition. (b) The singleton advantage disappeared when simulations were run with either the marginal or joint updating model under the same parameters.

We finally showed that the singleton effect might be explained by the reliance on the joint updating. When we controlled for the weight of joint updating, the condition effect disappeared (Fig. R7b; see also our reply to the above question). Together, our results suggest that the performance differences cannot be accounted for by slower learning alone and singleton training provides an additional benefit in learning by making participants learn more optimally. We have now included these points in the manuscript (Figs. S9a–d and Fig. S10).

1b) The model is not evaluated against rich behavioral dynamics in the training phase. Currently, model predictions are compared primarily to test performance. However, the human data from the training phase contain several informative patterns that could be used to further evaluate the model. For instance, while the singleton group shows a performance drop from training to test, the parallel group maintains its level of performance (e.g., Fig 2E)—can the model capture this? Similarly, the highly skewed parallel condition even surpasses the singleton group in training performance (Fig. 4C). Additionally, the ascending and descending curricula diverge during training (e.g., the ascending curriculum stagnates earlier; Fig. S4C), which the current model cannot account for under convergence assumptions (see Major Comment 1a). Even if the model fails to capture these

effects, attempting to fit them would highlight its limitations and could inspire needed model refinements for future work.

Reply: We thank the reviewer for this valuable suggestion, which encouraged us to capture the learning dynamics across different training curricula. To address this, we fit participants' trial-by-trial choices using our hybrid learning model and compared the model recovered accuracy curves with the observed ones.

The results showed that the model successfully captured participants' learning dynamics. Specifically, the predicted accuracy curves closely matched the empirical ones for the singleton and parallel conditions in Exp.1–3 (Fig. R18a), the skewed high and skewed low conditions in Exp. 4 (Fig. R18b), and even the ascending and descending conditions in Exp.6 (Fig. R18c). These findings demonstrate that the hybrid learning model provides a good account of both training and test performance, reinforcing its validity for the current task. We have revised our manuscript to include these new results (Fig. 3e, Fig. 4d and Figs. S17e&f).

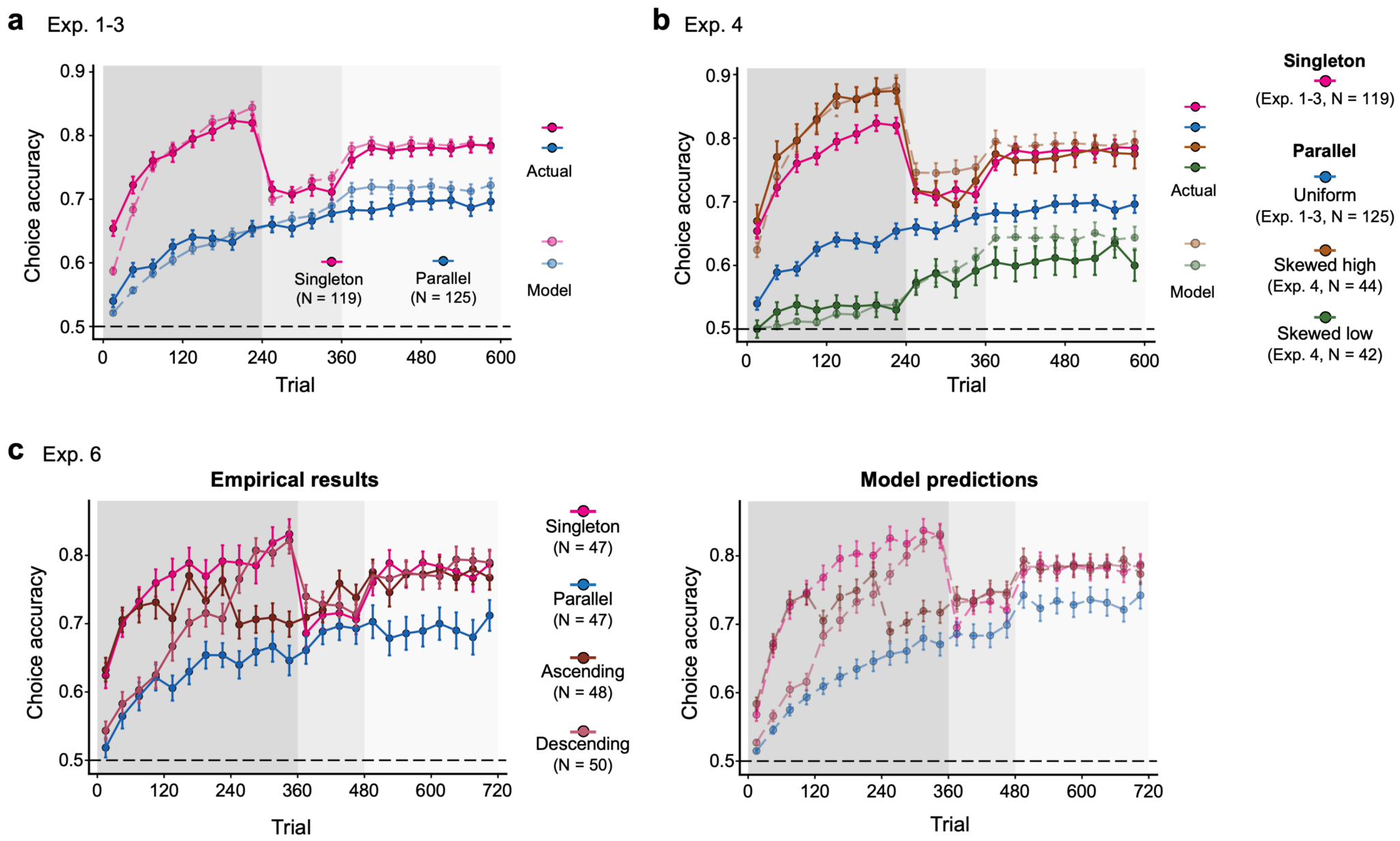

Fig. R18. Empirical results and model predictions. The accuracy curves recovered by the hybrid SD model closely match the empirical accuracy curves in singleton and parallel conditions (a), in skewed high and skewed low conditions (b), and in ascending and descending conditions (c).

1c) The boundedly rationality model's credit assignment assumptions would benefit from clarification and comparison. The model is said to associate outcomes with single cues rather than integrating across them. E.g., it is stated that "the model determines [the outcome] y based solely on cue i, rather than integrating evidence across all cues" (p. 26). However, this statement is somewhat ambiguous and would benefit from formalization—ideally with an explicit equation. Moreover, several different interpretations of this assumption are possible, each corresponding to a distinct cognitive mechanism:

(a) Participants attend to a single cue (e.g., the most informative one) and base their decision on it, updating only that cue.

(b) Participants integrate multiple cues when making a decision, but update only one (e.g., the most informative) cue per trial.

(c) Participants integrate multiple cues when making decisions and update all cues independently (which seems to be aligned with the current model's implementation).

These different mechanisms could lead to different behavioral patterns and may be supported or refuted by different aspects of the data. A proper model comparison would help distinguish them to better understand which boundedly rational mechanisms underlie curriculum learning.

Reply: We thank the reviewer for highlighting the need to (i) clarify our modeling assumptions and (ii) to formally compare alternative mechanisms. Per the reviewer's suggestion, we first revised the Methods section to provide explicit equations describing our models, along with clearer explanations and illustrations. For what we now call the marginal updating model (formerly the boundedly rational learner model), the prediction error used to update the weight of each presented cue i is determined by the observed outcome and the prediction made solely by each cue:

$$\delta_{ij}^{M} = r - y_{ij}$$

where δ_{ij}^{M} represents the marginal updating prediction error for cue i given the chosen option j, and r is the observed outcome which equals 1 if the selected option is rewarded and 0 otherwise. y_{ij} is the predicted reward probability for option j based on cue i alone, computed as:

$$y_{ij} = \frac{e^{\beta_1 \cdot w_{ij}}}{\sum_{u=1}^{2} e^{\beta_1 \cdot w_{iu}}}$$

w_{ij} represents the weight linking cue i to option j, and β_1 is the invers temperature governing choice stochasticity in single-cue trials. To be noted, the weight of all presented cues, instead of the most informative one, is updated (see below for model comparison between models with weights of all presented cues updated and with weight of only the most informative cue updated). We have revised the Methods to include these clarifications and more detailed descriptions of our models (revisions in L2P32–L9P37).

Regarding the second point, we formally tested alternative formulations. As suggested by the reviewer, we compared models assuming that participants (i) based decisions and updates on only the most informative cue (denoted as M1 in the reply), (ii) based decisions on all cues but updated only the most informative cue (M2), or (iii) based decisions on all cues and updated all cues (M3). Here we only considered the marginal updating which attributes the credit to each cue independent of other presented cues, and the results remained similar for other learning strategies. The results showed that in parallel condition where participants were trained with multiple cues per trial, M3 provided a better fit than M1 or M2, as indicated by lower AIC values (M3: 660.1$\pm$11.62 (Mean$\pm$SEM); M1: 716.3$\pm$6.90; M2: 693.1$\pm$9.16). These results suggest that participants in our task attended to and updated all cues in a trial. However, it could be possible that in more complex contexts (e.g., 10 cues per trial), participants may only attend to some of the cues given the cognitive resource limitation.

We also examined other alternative models within the broader hybrid learning framework (see our reply to Question 2 by Reviewer 1 for more details). Here we briefly summarize the results. First, in the singleton condition, participants' behavior could not be better accounted for by either the absence of learning during three-cue training trials or by assuming different learning rates for one-cue and three-cue trials (Fig. R9). Second, the observed patterns in both training conditions were not explained by a strategy in which updating strategy was simply determined by reward outcomes or trial number (Fig. R10a). Third, learning behavior depended on the specific assignment of updating strategies to each trial, not just the overall weight of joint updating (Fig. R10b). Finally, although incorporating

features such as decay in learning rates (Fig. R11) or attentional biases across cues (Fig. R12) improved model fits, the hybrid learning model continued to outperform other single-strategy alternatives.

Importantly, the model assuming participants assign different attentional weights to different cues (Leong et al., 2017), partly captures the reviewer's idea that participants may only attend to some salient cues (e.g., the most informative one). This model outperforms the original model without attentional bias (Fig. R12a), suggesting that participants distributed their attention unequally to different cues. More interestingly, we found that the attentional weights estimated by the model showed an inverted U-shaped relationship with the objective WOE values: cues with more extreme WOE value were assigned less weight. (Fig. R12b). This pattern is consistent with the "robust averaging" effect (De Gardelle & Summerfield, 2011, Li et al., 2017), where decision makers down-weight extreme information to avoid being misled. For the reviewer's reference, we copy the results of "robust averaging" identified by De Gardelle & Summerfield (2011) below (Fig. R19).

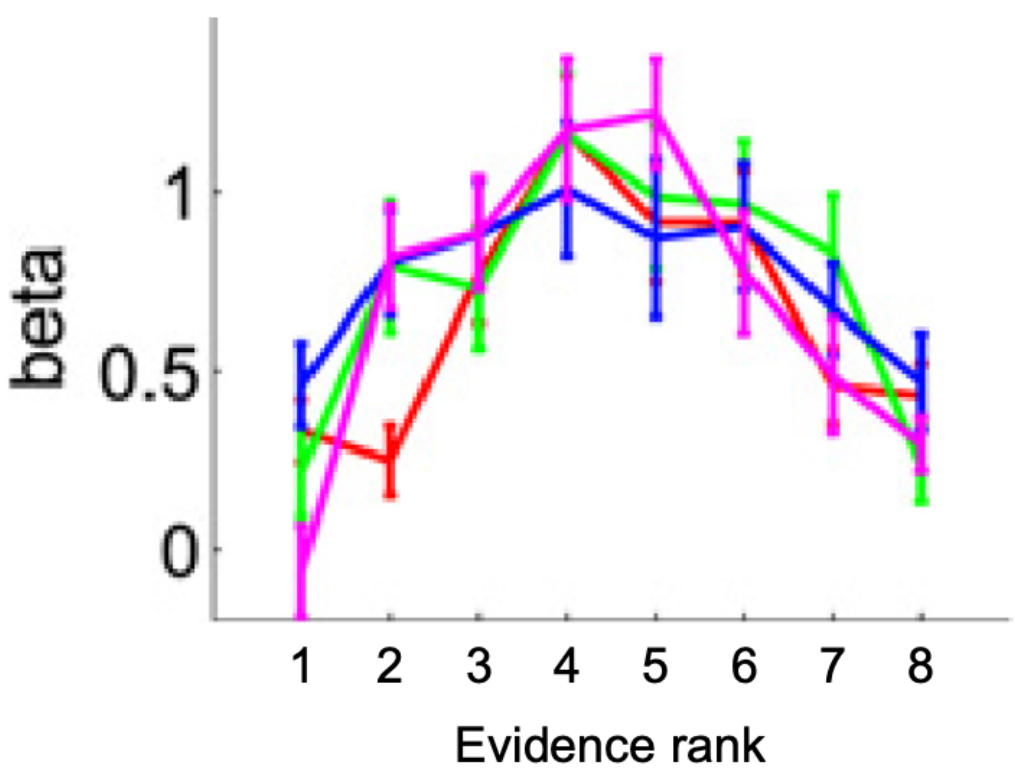

Fig. R19. Robust averaging effect copied from Fig. 3a in De Gardelle & Summerfield (2011). People assign lower weight to extreme cues and higher weight to intermediate cues, yielding an inverted-U relationship between cue extremity and weight.

In summary, our results by model estimation and comparison robustly support a hybrid learning mechanism that participants arbitrate between a marginal and a joint learning strategy. Although incorporating decay in learning rate and attentional weights improved fitting, the hybrid model still outperformed those single-strategy models with these features. For simplicity, we mainly emphasized the hybrid learning mechanism and used the original hybrid learning model without these additional features for data analyses while recognizing these extensions may capture further nuances.

- **Leong, Y. C., Radulescu, A., Daniel, R., DeWoskin, V. & Niv, Y. Dynamic Interaction between Reinforcement Learning and Attention in Multidimensional Environments. Neuron 93, 451–463 (2017).**
- **De Gardelle, V. & Summerfield, C. Robust averaging during perceptual judgment. Proc. Natl. Acad. Sci. U.S.A. 108, 13341–13346 (2011).**
- **Li, V., Herce Castañón, S., Solomon, J. A., Vandormael, H. & Summerfield, C. Robust averaging protects decisions from noise in neural computations. PLoS Comput Biol 13, e1005723 (2017).**

2. Potential inflation of Type I error in t-tests on model-derived estimates. The study performs second-level t-tests on subjective weights of evidence (WOE) estimated via first-level logistic regressions of the cue weights for individual participants. This approach can be problematic because the t-test

treats the WOE estimates as point estimates, ignoring the uncertainty associated with the initial estimation of the cue values via the logistic regression. This disregard for estimation variance can inflate Type I error rates and, in the worst case, lead to false positives when comparing WOE values across conditions. There are several ways the authors could address this issue. E.g., they could apply a Bayesian hierarchical regression which would allow for simultaneous modeling of within-subject uncertainty and between-group differences. Alternatively, they could weight each participant's WOE estimate in the t-test by the inverse of its standard error to account for differing levels of estimation reliability.

Reply: We thank the reviewer for this helpful suggestion. To address the concern, we re-analyzed the WOE estimates by accounting for estimation reliability. Specifically, we weighted each participant's WOE value by the inverse of its squared standard error. For each participant, the adjusted WOE value for cue i becomes:

$$w_i^{adj} = w_i \times \frac{1/se_i^2}{\sum_{k=1}^{6} 1/se_k^2}$$

where w_i is the original WOE value extracted from the logistic regression and the se_i represents the estimated standard error of w_i. We have revised the Methods to include this point (revisions in L5P31–L9P31).

Using these adjusted WOE values, we replicated our original findings: participants in the singleton condition still learned larger WOE values than those in the parallel condition (Fig. R20). We have updated the results in the manuscript accordingly (Figs. 1f and 4c).

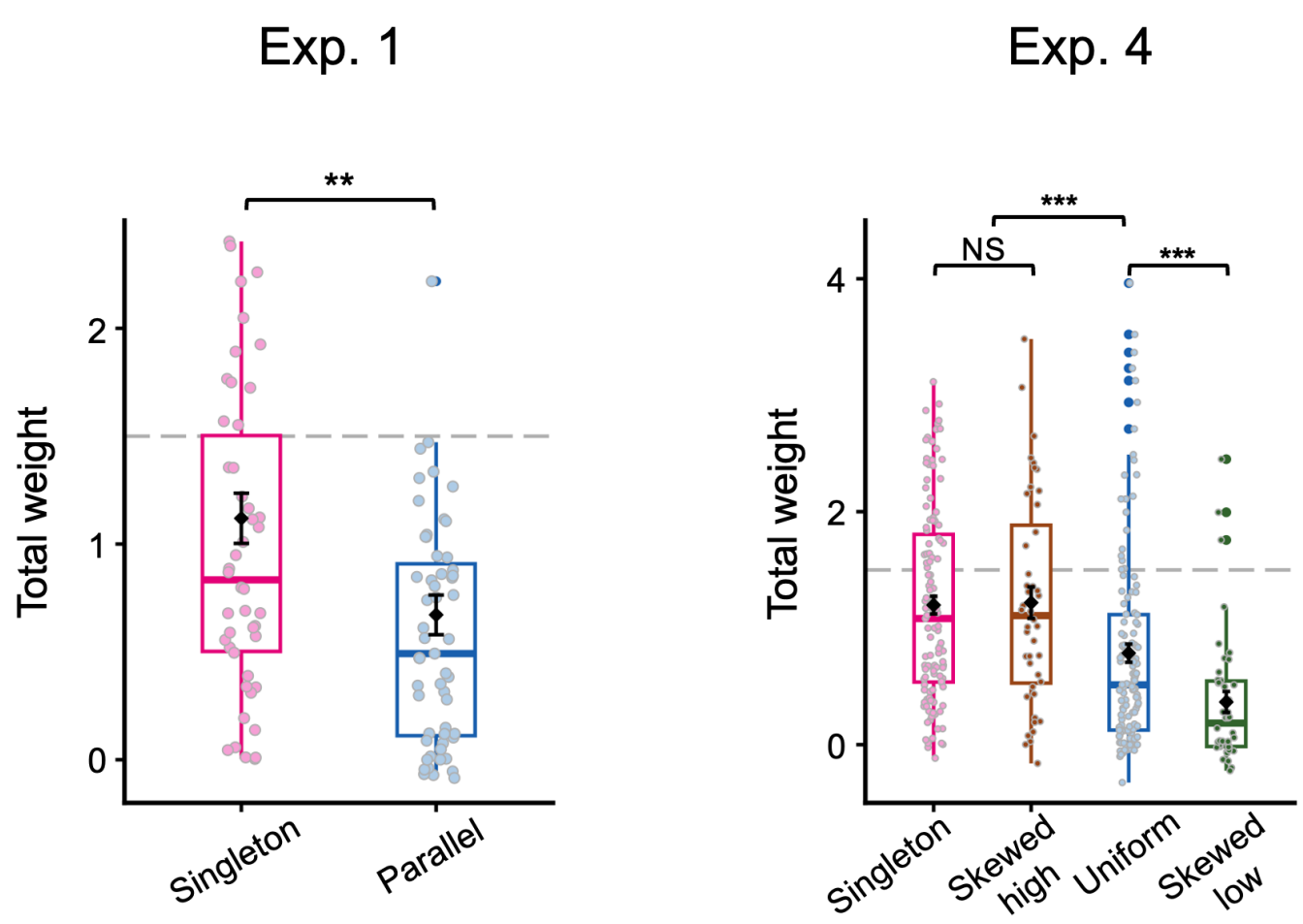

Fig. R20. Total weight calculated by adjusted subjective WOE values. Total weight was computed by aggregating subjective WOEs after weighting each cue by its estimation reliability from the logistic regressions.

3. Clarification of WOE distribution comparability between Experiment 4 and Experiments 1–3. Experiment 4 applies a skewed distribution of the summed weights of evidence (WOEs). However, it's unclear whether the cue value distributions in both conditions of Experiments 1–3 are directly comparable to those in Experiment 4. If the underlying cue frequencies differ substantially this could confound the interpretation of results, as differences in WOE might then be driven by cue exposure frequency rather than by curriculum structure per se. While I suspect that the cue presentation frequencies are, in fact, matched across experiments, this is not clearly stated in the manuscript (unless I missed it). The right panel of Figure 4A suggests matching within Experiment 4 conditions, but not necessarily across experiments. Explicitly confirming the latter would help rule out alternative explanations

Reply: we thank the reviewer for this helpful comment and apologize for not making this point clearer in the manuscript. As the reviewer suspected, we carefully designed our stimuli to make the distribution of cue frequency across all conditions and all experiments matched, including Exp. 4. Specifically, each of the six different cues appears uniformly in any conditions. The critical difference lies in the distribution of cue combinations—that is, the curriculum structure—which accounts for the observed variation in learning across conditions.

Per the reviewer's suggestion, we have revised our manuscript to explicitly highlight this point (revisions in L38P12–L1P13).

4. Potential interaction between motivation and cognitive fatigue in Experiment 2. Experiment 2 tests whether the performance differences between the singleton and parallel groups can be attributed to differences in motivation by including a trailing task at the end of the experiment. The finding that both groups perform equally well on this added task is interpreted as evidence that motivation does not differ between conditions. However, I wonder whether the addition of two extra blocks may have enhanced cognitive fatigue toward the end of the experiment, potentially masking motivational differences that were present earlier on. While this possibility is speculative, it leaves open the alternative interpretation that initial performance differences between groups may still reflect early motivational differences—effects that may diminish over time due to fatigue or disengagement. One way to address this would be to examine whether performance differences between groups change over time, particularly during the last Testing Phase. If motivation-related differences were present early on but faded due to fatigue, one would expect a convergence in performance between conditions in the final blocks of the Testing Phase. Alternatively, the authors could rule out this explanation by showing that models incorporating (motivation-related) differences in learning rate (see Major Comment 1a) do not account for the data as well as the proposed boundedly rationality model.

   Reply: We thank the reviewer for this excellent suggestion to more directly assess whether the singleton effect could be explained by motivational differences.

   Following the reviewer's suggestion, we first examined whether group performance differences changed over time during the testing phase. If initial differences reflected motivation that later declined due to fatigue, we would expect a significant interaction between trial number and condition in predicting the testing performance. However, no such interaction was observed ($\beta_{trial \times condition}$ = 0.0002, *SE* = 0.00029, *z* = 0.70, *P* = 0.487), suggesting comparable motivation across groups during testing.

   Second, we tested this possibility with computational modeling, treating motivation or task engagement as a decay in learning rates. If motivation were lower in the parallel condition, we would expect a stronger decay. The analysis revealed no significant difference in the decay parameter between groups (Fig. R9b; *t(221.2)* = -1.7, *P* = 0.09).

   Finally, a direct comparison in the reaction times (RTs) at the testing phase showed no significant differences in the RTs between conditions (Exp. 1–3: β = -0.02, *SE* = 0.042, *t*(241.4) = 0.52, *P* = 0.603). If participants in the parallel condition had been less motivated, they would likely have responded faster to complete the task more quickly.

Taken together, these results indicate that the singleton effect is unlikely to be driven by differences in motivation. We have revised the manuscript to include these analyses and their interpretations (revisions in L28P6–L36P6).

Minor Comments

5. P. 18: It might be worth adding a table or figure to illustrate the distribution of WOEs in Experiment 1 for the parallel condition.

   Reply: We thank the reviewer for this helpful suggestion. In response, we have added a figure illustrating the distribution of summed objective WOE values, as well as the distribution of individual cues in the training trials under both singleton and parallel conditions (Fig. R21). While the summed objective values differ across conditions, the frequency of each cue remains uniformly distributed and identical between conditions.

   We have revised our manuscript to include this clarification (Fig. S2c).

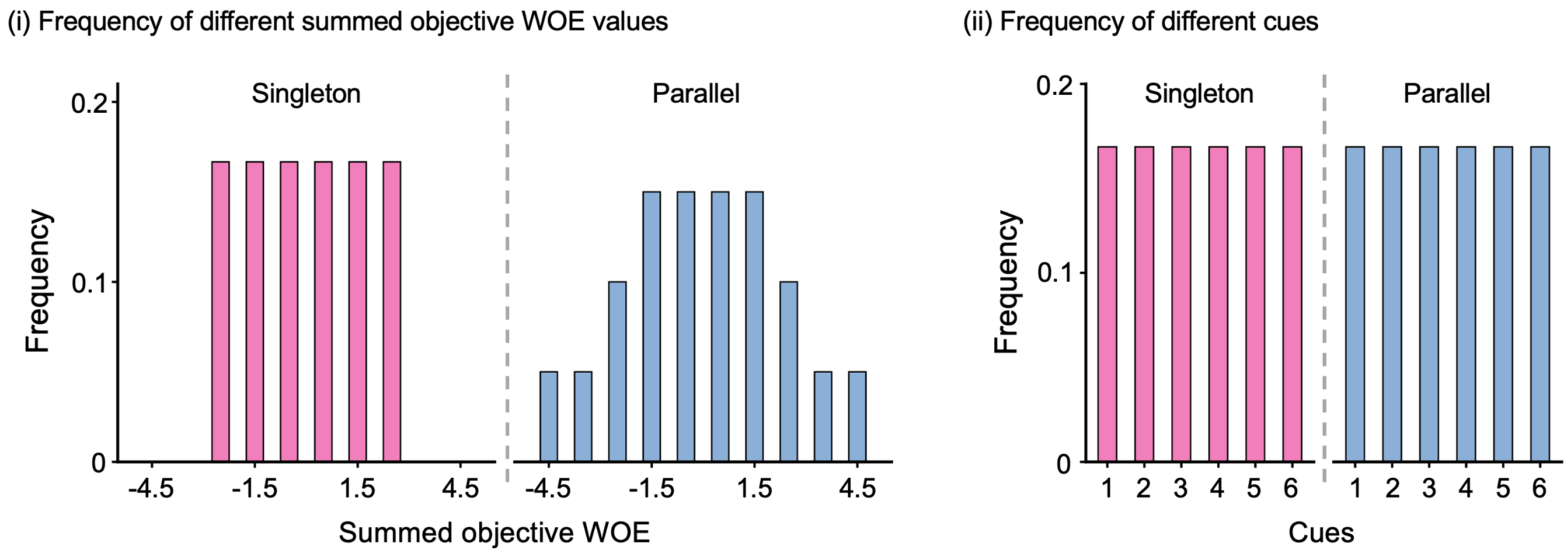

Fig. R21. Distribution of summed objective WOE values and cue frequencies for singleton and parallel training.

6. P. 25: The authors might want to clarify whether the testing phase of Experiment 5 was still stochastic (and thus comparable to the other experiments).

   Reply: We thank the reviewer for pointing out the need for clearer clarification. The reviewer is correct: the testing phase of Exp. 5 remained stochastic and was designed to be directly comparable to other experiments. This ensured that performance during testing could be meaningfully compared across conditions. We have updated our manuscript to make this point explicit (revisions in L22P29–L24P29).

7. The manuscript would benefit from differentiating exploratory from confirmatory hypotheses (the latter of which pertain to the predictions of the boundedly rationality model) statistically, and applying appropriate constraints to hypothesis testing (e.g., two-tailed vs. one-tailed t-tests).

   Reply: We thank the reviewer for this suggestion. In the initial submission, we reported all statistics using two-tailed tests as a conservative default. In the revision, we now distinguish clearly between exploratory and confirmatory hypotheses and apply one-tailed tests when a directional prediction is pre-specified. This adjustment increases the sensitivity of our analyses while maintaining rigorous standards of inference.

Dear Editor and Reviewers,

Thank you for your thoughtful and constructive feedback on our manuscript entitled “Human curriculum learning of a cue combination task.” We have addressed all comments carefully and believe the revision substantially improves both the clarity and the rigor of the work. All changes are highlighted in blue in the revised manuscript for the convenience of review, and a detailed point-by-point response follows.

Below we briefly summarize the revisions we have made to address the raised concerns.

1. Clarification of framing the follow-up experiments as predictive. The reviewer expressed concern that parts of the manuscript described follow-up experiments as “model predictions” despite revisions to the original modelling framework. We have revised the text to clearly separate (i) qualitative predictions that follow from our core theoretical claim—namely, that learners do not rely exclusively on joint updating but also use a simpler marginal updating strategy under cognitive constraints—from (ii) the role of the computational model in formalizing this claim and quantifying its implications. We also clarify that the revised model improves quantitative alignment with behaviour, without implying that results are post hoc.

2. Justification of the hybrid model used for main analyses. The reviewer noted that models incorporating attentional weights achieve better fit by AIC than the hybrid model used for the main analyses. We now explain explicitly why we retain the hybrid model without extensions as the reference model: it isolates the mechanism central to our questions—arbitration between marginal and joint updating—using a minimal and interpretable parameter set. We also show that when model complexity is penalized more strongly (BIC), attentional extensions no longer outperform the reference hybrid model, and the qualitative conclusions are unchanged.

3. Addressing modelling choices and robustness. We now motivate the threshold-based hybrid formulation and show that a continuous-mixture variant yields comparable fits and the same qualitative conclusions. We also redid model and parameter recovery using single-simulation procedures matched to the experimental data size, ensuring recoverability is assessed under the same conditions as the empirical analysis. In addition, we removed Bayesian model selection metrics that are not appropriate for AIC-based comparisons. We also explored model comparisons with BIC and observed unchanged results that the hybrid model outperformed single strategy alternatives, and found our main modelling results were robust across different experiments.

4. Refinement of the statistical analyses and interpretations. In response to concerns about parametric tests and potential over-interpretation of null effects, we replaced correlation analyses with non-parametric alternatives where appropriate and tempered interpretations of reaction-time and decay effects. We also clarify more directly why the singleton advantage cannot be exclusively explained by response speed or by motivation difference indexed by the fitted learning parameters.

5. Positioning novelty relative to prior literature. We revised the Discussion to acknowledge prior work demonstrating dynamic arbitration between learning strategies. The novelty of the present study is now framed more precisely: not in dynamic mixture weights per se, but in showing how curriculum structure systematically shapes strategy arbitration by altering the trade-off between learning precision and cognitive cost, and how this insight can be used prospectively to design effective curricula.

6. Additional clarifications. We also addressed several additional points, including (i) clarifying whether recovered curves reflect predictive versus generative performance, (ii) explaining why joint

updating is inferred less often in the parallel condition, (iii) reporting fitted parameter distributions, and (iv) clarifying why certain simulation descriptions are retained in Methods.

We appreciate the time and efforts invested by the Editor and the Reviewers. We hope the revised manuscript addresses all outstanding concerns, and we would be happy to provide any further information if needed.

Reviewer #1 (Remarks to the Author):

0. The reviewers did a thorough revision of the paper in response to the reviewers' concerns, resulting in a fully novel theoretical framework, as well as one novel experiment. I appreciate the effort to improve the paper, in particular the modeling, in response to our major concerns; the novel experiment helps frame the generalizability of the findings, making the authors broad claims more in line. Given that important parts of the paper are changed, I am treating them like a "first round" review. I am satisfied with responses and edits to the model-independent parts of the results. The novel theoretical framework is much more valid than the previous, which results in a major improvement in the paper. There remain important limitations to the modeling, which I detail below; as well as other issues.

   Reply: Thank you for the careful re-evaluation and for the positive assessment of the revisions. We appreciate the reviewer's continued critical engagement and the identification of remaining limitations. Following these suggestions, we have revised the manuscript further and address each of the remaining points in detail below. We believe these additional clarifications and analyses further improve the work and strengthen its contribution.

Model comparison

1. AIC is not an approximation of model evidence, and thus should not be used to compute protected exceedance probability (contrary to BIC). The results as presented are sufficient without the inclusion of pxp.

   Reply: Thank you for pointing this out. Per the reviewer's suggestion, we have now removed all results and references related to Bayesian model selection and protected exceedance probabilities from the revised manuscript.

2. Fig. S8: the model recovery procedure is inadequate and inflates the recoverability results (both model identifiability and parameter identifiability). Specifically, averaging out over simulations as is done here (to decrease simulation noise) leads to a model comparison procedure that is not the same as that applied to the experimental data. Most (reasonable) models will be very recoverable given enough data to average over; the recovery procedure is meant to ask whether the model is recoverable *in the given experimental context* and consequently is not meaningful when done in a different context, with different data size. Since the results of this paper rely strongly on interpretation of model comparison, it is important to ensure that model comparison is interpretable; the recovery (both model and parameters) should be redone applying the same procedure as for experimental datasets.

   Reply: Thank you for this important comment and agree with the concern that averaging out over simulations is not an adequate way to present the results of the model and parameter recovery.

   In response, we re-ran both model and parameter recovery analyses using single simulations, matching the experimental data size and applying the identical fitting and comparison procedures used for participants' data. As expected, recoverability decreased modestly relative to the averaged analysis, but remained acceptable (**Fig. S8**). Parameter recovery similarly showed increased variance; however, recovered parameters remained significantly correlated with their true values across all parameters (**Fig. S8**), indicating reliable identifiability under the experimental conditions.

   We have updated the manuscript to reflect these revised recovery analyses and now report results based exclusively on single-simulation recovery (see below).

Revised Fig. S8 (pages 50-51):

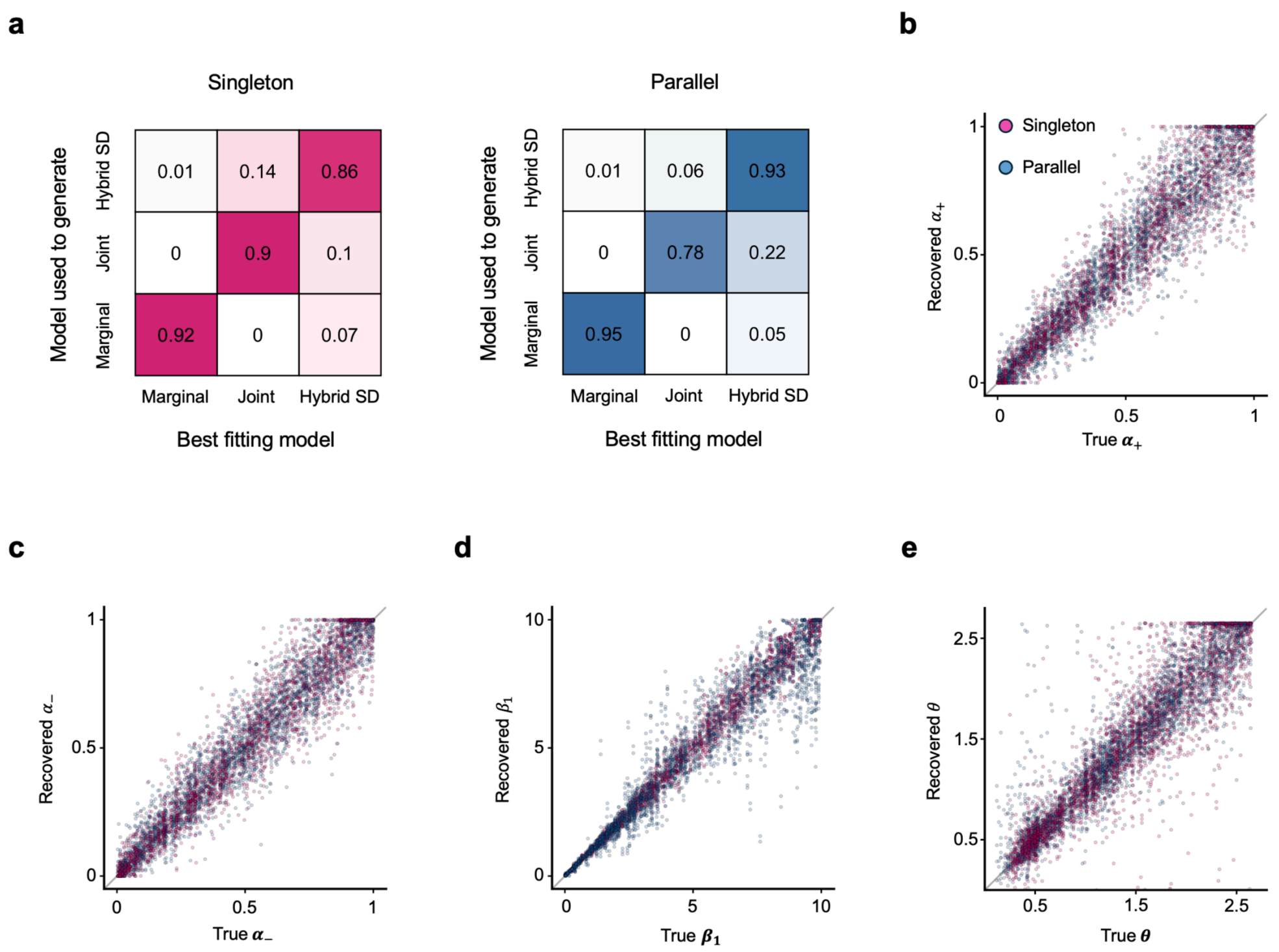

Fig. S8. Model and parameter recovery. (**a**) Model recovery. The model recovery focuses on the hybrid SD, pure marginal and pure joint updating models without relevant extensions (e.g., learning rate decay and attentional bias), since the central theoretical claim of this study is that participants rely on a hybrid learning mechanism rather than a single updating strategy. Specifically, we aimed to examine whether the hybrid updating model could be reliably distinguished from the single-strategy alternatives. Choices were first simulated under each model using parameter values randomly sampled from predefined distributions. The simulated data were then fitted with each candidate model, and the best-fitting model was identified based on the AIC value. For each participant, we ran five simulation-estimation loops with identical parameter values and repeated the full procedure five times (25 simulations total). Model recoverability was assessed by pooling data across all participants and computing the probability that each model produced the lowest AIC. Results are shown separately for singleton and parallel conditions. In both conditions, the model used to generate the simulated data was also most likely to be recovered, demonstrating that these models are distinguishable. The number in each cell represents the probability of each candidate model being the best model in capturing the behavioral choices simulated by one specific model of each row. To be noted, the sum of some rows is not identical to 1 because of rounding. (**b–e**) Parameter recovery results for hybrid SD model. We further tested whether the parameters of the hybrid SD model could be reliably estimated. Using the same procedure as for model recovery, we examined the correlation between true parameter values and those recovered from model fitting. For all four parameters (α_+, α_-, β_1 and θ), the recovered values were strongly correlated with the true values, confirming reliable parameter identifiability. Each dot represents a data point from one participant; colors indicate experimental condition.

3. Fig. S14 the hybrid model used for the main results (if I understand correctly) in the paper has a significantly worse fit than marginal/joint/hybrid models with attention. My understanding that this is

the model used for the main results comes from other figures showing main model parameters, not including attention weights; if I am mistaken, please make sure to clarify. If I am not mistaken, then this is problematic and needs to either be changed (so the main text reflects the results from the actual winning model) or the rationale for picking the *4th* best winning model should be defended.

Reply: Thank you for raising this thoughtful question. You are correct that the hybrid model we used for main results has a worse fit than the models with attentional bias, based on the AIC value (but see model comparisons based on the BIC below). However, we chose to retain the hybrid learning model without extensions as the primary model for the main analyses for conceptual and interpretative reasons, rather than purely based on fit.

Specifically, our central goal is to understand how curriculum structure shapes learning/ credit-assignment strategies, particularly the dynamic arbitration between marginal and joint updating. The original hybrid learning model directly captures this core mechanism with a minimal set of parameters, allowing clear interpretation of learning strategies, individual differences, and trial-by-trial dynamics. In contrast, attentional models introduce additional parameters that improve fit but primarily refine how cues are weighted, rather than which learning strategy is used. Importantly, these attentional extensions do not change our core conclusions: the hybrid learning mechanism remains necessary to explain behavior. We therefore treat attentional models as meaningful extensions rather than competitors to the hybrid learning framework.

In addition, when model complexity is penalized more strongly using BIC, attentional models no longer outperform the hybrid model without attentional bias and in fact show worse fit, whereas hybrid models continue to outperform single-strategy alternatives (**Fig. R1**). Thus, across comparison criteria, the key conclusion—that hybrid learning provides the better account of behavior than single strategy learning—remains unchanged.

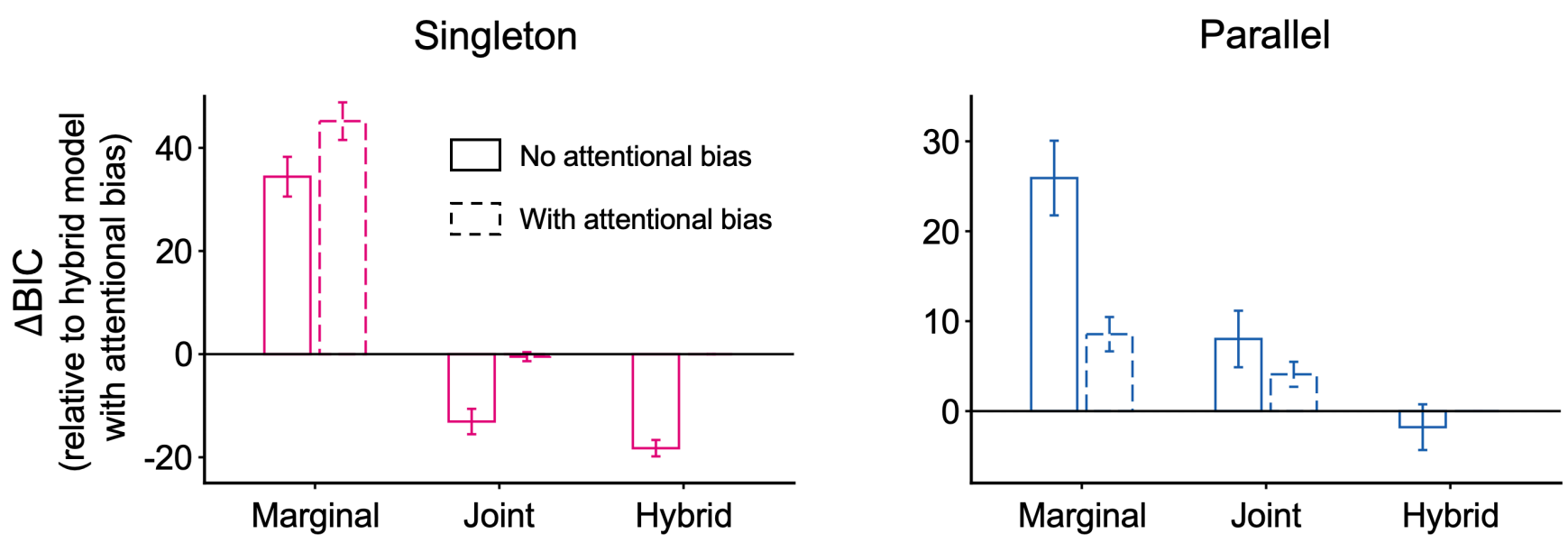

Fig. R1. Model comparison using BIC in singleton (left) and parallel (right) conditions. In both conditions, the hybrid model without attentional bias shows lower BIC values than those alternatives.

We have revised the manuscript to clarify this rationale and to explicitly state why the hybrid model without attentional weights serves as the reference model for the main analyses.

Revised Results (lines 320-326):

> …Finally, although extensions such as learning-rate decay or cue-specific attentional bias improved overall model fit, the hybrid learning model continued to outperform single-strategy models (**Fig. S14**). We retained the hybrid model without extensions as the reference model for the main analyses, as it directly captures the core theoretical mechanism—the arbitration between marginal and joint updating—with a minimal and interpretable parameter set. While these extensions enhance quantitative fit, they do not alter the qualitative conclusions or the necessity of a hybrid learning mechanism.

Model rationale and interpretation.

4. Hybrid model– l 34. The authors discretize the mixture of experts in the model, according to a discrete threshold, and only justify this by saying "for simplicity". This is not obvious to me – most other mixture approaches do not do this discretization and are not particularly complex. This appears to be an ad hoc choice for the modeling that is not a priori likely and is not justified. The authors should clarify the reasons behind this choice, and explain whether it is core to the model (i.e. whether relaxing that assumption to the standard formulation changes the results/fits worse).

   Reply: Thank you for this great point. Our central theoretical claim is that learners arbitrate between two distinct learning strategies when multiple cues are present. The threshold-based (discretized) hybrid model is one implementation of this idea, chosen to make the strategy-switching explicit and easy to interpret: on each trial, learners rely predominantly on one strategy or the other depending on whether the expected benefit exceeds a criterion.

   Importantly, this discretization is not core to the theory. We view threshold-based and continuous-weight mixtures as two variants of the same hybrid learning framework. To address the reviewer's concern directly, we implemented and fit a continuous mixture version in which the weight on joint updating (i.e., γ) varies smoothly as a function of cue discriminability. Specifically, the weight on joint updating at trial *t* is given by a logistic function of the standard deviation (SD) of the learned cue weights:

$$\gamma_t = \frac{1}{1 + exp(-\beta_\gamma \cdot (SD - \theta))}$$

   where β_γ represents an additional free parameter, capturing the sensitivity to SD. The threshold-based model corresponds to the limiting case where $\beta_\gamma \to \infty$.

   Fitting this continuous model to individual participants' choices yielded results consistent with the threshold-based model: fit was comparable in the singleton condition and better in the parallel condition by AIC (singleton: ΔAIC = 0.06 $\pm$ 0.38 (mean $\pm$ SEM), $t(118) = 0.16$, $P = 0.87$; parallel: ΔAIC = -4.65 $\pm$ 1.19, $t(124) = -3.90$, $P < 0.001$). In practice, the threshold-based model offers similar explanatory power with fewer parameters and clearer trial-by-trial interpretation of strategy use, which motivated its use in the main text.

   We have revised the manuscript to clarify this rationale and to state explicitly that relaxing the discretization to a continuous mixture does not alter the results. This makes clear that our conclusions do not depend on an ad hoc modeling choice, but on the broader principle of hybrid strategy arbitration.

   Revised Results (lines 238-244):

   > For clarity and interpretability, we used a threshold-based rule to implement the arbitration between different learning strategies in the hybrid adaptive models. Specifically, γ is set to either 0 (i.e., marginal updating) or 1 (i.e., joint updating) in each training trial, meaning the learning strategy switches between marginal and joint updating. This discretization is not central to the theory: a continuous mixture in which γ varies smoothly between 0 and 1 yields comparable fits and the same conclusions. We therefore retain the threshold-based formulation as a parsimonious and interpretable implementation of hybrid learning.

5. The novelty claim for dynamic updating in the discussion (l13—14 p17) is quite wrong, as many other mixture processes in published papers include dynamically changing mixture weights, for

example Miller, Shenhav & Ludvig 2019, Collins & Frank 2012, Frank & Badre 2012, and many others.

Reply: We thank the reviewer for highlighting these important precedents and agree with the point. We did not intend to claim that dynamically changing mixture weights are novel per se. As the reviewer notes, a large body of prior work has demonstrated dynamic arbitration between learning strategies and has also explored the underlying neural mechanisms (e.g., Lee et al., 2014; Charpentier et al., 2020).

We have revised the Discussion to clarify our contribution relative to this literature. The novelty of our work lies not in the existence of dynamic arbitration between learning strategies, but in how curriculum structure shapes strategy arbitration over learning, and in linking this arbitration to a concrete trade-off between learning precision and cognitive cost in a cue-combination task. In particular, our framework shows how different training curricula bias learners toward different regions of the strategy space and thereby alter learning outcomes, and how this mechanism can be used prospectively to design and validate new curricula.

Revised Discussion (lines 458-474):

> The use of a hybrid learning mechanism have been widely observed from non-social reinforcement learning to strategic decision-making[38–42]. In the classic two-stage learning task[39], for example, learners arbitrate between model-free and model-based strategies. Importantly, prior work has shown that the weighting of different learning and control strategies adjusts dynamically with context, experience, and reliability signals[40,41,46–49], and has identified neural mechanisms supporting this arbitration process[40,41,49]. Our study builds on this literature by examining how training curricula shape such strategy arbitration during learning in a cue-combination task.
>
> Specifically, we show how different curricula bias learners toward different regions of the strategy space by altering the relative benefits and costs of alternative learning strategies. From a resource-rational perspective[33–35], learners should weigh the benefits of a learning strategy against its cognitive cost, selecting the one that maximizes expected utility under the resource constraints. Consistent with this view, we find that learners rely more on joint updating when its precision advantage outweighs its additional effort relative to marginal updating, and that curriculum structure systematically influences this trade-off. This framework thus helps explain why certain curricula are more effective than others and provides a principled basis for designing training regimes that improve learning outcomes.

- Lee, S. W., Shimojo, S., & O'Doherty, J. P. (2014). Neural Computations Underlying Arbitration between Model-Based and Model-free Learning. *Neuron*, 81(3), 687–699. https://doi.org/10.1016/j.neuron.2013.11.028
- Charpentier, C. J., Iigaya, K., & O'Doherty, J. P. (2020). A Neuro-computational Account of Arbitration between Choice Imitation and Goal Emulation during Human Observational Learning. *Neuron*, 1–21. https://doi.org/10.1016/j.neuron.2020.02.028

6. It seems very misleading that parts of the paper (e.g. follow up experiments, predictions) are claimed as model predictions, when we know that the model has been fully changed after this revision (e.g. p13/14). If the authors feel that their theory made the prediction independently of the model implementation, then they should express this more accurately and justify it; if it doesn't, then they should refrain from making prediction claims where they are in fact postdictive.

Reply: Thank you for this comment and agree that it is essential to be precise about what constitutes a genuine prediction. We would like to clarify that the key predictions in our study stem from a theoretical hypothesis that remained unchanged throughout the revision process, rather than from a specific implementation detail of the model.

Specifically, the central theoretical claim underlying the singleton effect is that learners do not rely exclusively on optimal joint updating, but instead often use a simpler, marginal updating strategy when multiple cues are present. This assumption was present in the original submission where we assumed that participants only use marginal updating and remains the foundation of the revised framework. Although the modelling implementation evolved—from a simplified assumption to a more rigorous, trial-by-trial hybrid model—the qualitative predictions follow directly from this core hypothesis. For example, predictions about when curricula should help or hinder learning depend on whether curricula promote the efficiency of marginal updating, not on the precise mathematical form of the model.

At the same time, the revised modelling framework plays an important role in formalizing and sharpening these theory-driven predictions. In particular, it improves the alignment between predicted and observed behaviour under new curricula. For instance, as the reviewer noted in the original submission, there was a gap between model predictions and the empirical results in Exp. 6; this gap is now resolved by the revised framework, which better captures trial-by-trial learning dynamics.

We agree that the manuscript should distinguish more clearly between predictions derived from the theoretical framework and results that are supported or refined by a particular model implementation. We have revised the text to make this distinction explicit. This ensures that our claims are not postdictive, while also accurately reflecting the role of the revised modelling framework.

Revised Results 1 (lines 228-231):

> Inspired by previous studies[38–42], we developed a hybrid learning framework in which learning reflects the combined influence of joint and marginal updating. Specifically, this framework assumes that participants don't rely exclusively on joint updating but often use a simpler marginal updating when credit assignment is challenging in multi-cue settings.

Revised Results 2 (lines 328-334):

> The hybrid learning model thus offered a solid foundation for predicting which curricula should enhance, impair, or have no effect on human learning. We next tested these out-of-sample predictions in three independent experiments. Importantly, these predictions follow from the framework's core theoretical claim—that learners rely partially on a simpler marginal updating strategy under cognitive resource constraints—rather than from any specific model implementation. The hybrid SD model serves to formalize and quantify this claim and to improve alignment between predicted and observed learning dynamics.

Statistical issues

7. Fig. S3, right panel: it is very puzzling that the authors report no effect of condition on RTs ($p=.5$), when the plot appears to show a clear effect. This should be at minimum double checked; and furthermore, is it possible that an inappropriate test is used that does not factor out relevant variance,

limiting the power in testing for condition? The use of this plot to argue against a speed accuracy tradeoff is not convincing: the null result looks like a type II error that is over interpreted.

Reply: Thank you for raising this concern and apologize for the lack of clarity in our original description. The RT analysis was conducted after controlling for task difficulty, indexed by the summed objective WOE values on each trial. Because the summed WOE values are not uniformly distributed across trials, and RT changes with difficulty (i.e., the summed WOE values), comparing raw RTs averaged across all trials would overweight more frequent WOE values and potentially produce a misleading condition effect.

To address this, we used a regression analysis that included both summed WOE values and a condition indicator as predictors of RT. This analysis revealed a strong effect of WOE on RT, confirming that harder trials elicited slower responses, but no reliable effect of condition once difficulty was accounted for. We have rechecked this analysis carefully and confirmed the result. In contrast, an analogous regression predicting choice accuracy showed significant effects of both WOE and condition, indicating that curriculum differences affect accuracy but not response speed when difficulty is held constant.

That said, the reviewer's intuition about the plot is correct: when RTs are averaged across all trials without controlling for difficulty, the parallel condition shows slightly faster responses than the singleton condition ($t(100.24) = 2.17$, $P = 0.032$). Our intent, however, is not to rule out speed–accuracy trade-offs in general, but to show that the observed accuracy advantage in the singleton condition is unlikely to be straightforwardly explained by slower responding alone.

We have revised the manuscript to clarify this analytical approach and to avoid over-interpreting the null RT result, as replicated below.

Revised Results (lines 93-98):

> …Critically, the advantage of singleton learning remained significant after controlling for the cue evidence (**Fig. S3a**; $\beta = 1.00$ (0.48, 1.52), $z = 3.73$, $P < 0.001$). By contrast, once cue evidence was accounted for, RT did not differ significantly between conditions (**Fig. S3b**; $\beta = -0.02$ (-0.14, 0.10), $t(105.3) = 0.29$, $P = 0.77$), indicating that the singleton advantage in accuracy is unlikely to be driven solely by slower responding.

8. P54 decay, please include the statistical test used and result. If it is a t-test as elsewhere, is it appropriate for this parameter, that likely has a skewed distribution? In any case, this again appears to lead to an over interpretation of a null result: decay has a multiplicative effect on learning rates, such that the numerically higher rate plotted in the figure could lead to significantly faster learning rate decrease in parallel than singleton. To conclude about motivation difference, it would be better to verify the effect on learning rates more directly.

   Reply: Thank you for pointing this out. For the decay parameter, we initially reported results from an independent-samples t-test, consistent with other analyses in the manuscript. To address concerns about distributional assumptions, we additionally conducted a non-parametric Wilcoxon rank-sum test. This analysis likewise showed no reliable difference between conditions ($W = 6428$, $P = 0.07$), although it suggested a weak trend toward greater decay in the parallel condition. We have now reported both tests to provide a more complete picture.

   Revised Legend of Fig. S14 (page 57):

…We found no reliable evidence that the decay parameter differed between conditions using either parametric or non-parametric tests (independent-samples t-test: $t(221.2) = -1.7$, $P = 0.09$, Cohen's $d = -0.22$ (-0.47, 0.03); Wilcoxon rank sum test: W = 6428, $P = 0.07$), consistent with comparable learning motivation across conditions.

Regarding the interpretation of decay and motivation, we agree that learning-rate decay acts multiplicatively and could, in principle, yield different learning dynamics even when mean differences are small. To address this concern more directly, we examined learning rates themselves. A direct between-condition comparison of the learning rates for all training trials would be inappropriate, however, because participants in the singleton condition experienced both one-cue and three-cue trials, whereas those in the parallel condition experienced only three-cue trials. Since credit assignment is inherently easier in one-cue trials, learning rates are expected to be higher in that context.

To make a fair comparison, we therefore re-estimated the model allowing separate learning rates for one-cue and three-cue trials and compared learning rates for three-cue trials only. This analysis revealed comparable learning rates across conditions for three-cue trials (α_+: 0.30$\pm$0.031 vs. 0.25$\pm$0.020 (singleton vs parallel), $t(204.3) = 1.24$, $P = 0.22$; α_-: 0.055 $\pm$0.013 vs. 0.06 $\pm$0.013, $t(241.5) = -0.32$, $P = 0.74$). As expected, learning rates were higher for one-cue trials overall (α_+: 0.56$\pm$0.035; α_-: 0.08$\pm$0.012), reflecting reduced credit-assignment demands. These results have been added to the revised manuscript.

Revised Results (lines 141-147):

…Finally, computational modelling revealed no reliable differences between the two conditions, either in learning-rate decay when learning rates were allowed to decay during training (**Fig. S14b**; $t(221.2) = -1.7$, $P = 0.09$, Cohen's $d = -0.22$ (-0.47, 0.03); Methods) or in the learning rates themselves for multi-cue trials when single-cue and multi-cue trials were allowed to have different learning rates in the singleton condition (α_+: $t(204.3) = 1.24$, $P = 0.22$, Cohen's $d = 0.16$ (-0.09, 0.41); α_-: $t(241.5) = -0.32$, $P = 0.74$, Cohen's $d = -0.04$ (-0.29, 0.21); Methods).

Finally, we want to emphasize that the singleton advantage cannot be explained solely by differences in learning parameters, including learning rates. Our simulation analyses showed that even when singleton and parallel learners were assigned identical parameter values, their performance diverged under different curricula (**Fig. S10**). This indicates that curriculum structure itself—rather than motivational differences indexed by learning rates or the decay—drives the observed effects. However, this doesn't mean that learning rates have no influence on learning performance. As shown in **Fig. S9f**, we observed a positive correlation between the value of the positive learning rate and the final learning performance.

9. More generally, the use of parametric stats is problematic in several places in the manuscript. In addition to the above two examples, see e.g. Fig. S15 d, where one of the measures is bounded, with many points at the bounds (0) leading to a very skewed (non-normal) distribution; linear regression inflates the results, a non-parametric (e.g. Spearman) or robust test should instead be used.

Reply: Thank you for highlighting this important issue. To address concerns about non-normal and bounded distributions in the correlation analyses, we systematically assessed the distributional

properties of the relevant measures and replaced parametric analyses (i.e., Pearson correlations) with appropriate non-parametric alternatives (i.e., Spearman rank correlations) wherever assumptions of normality were not met. Importantly, this change does not affect our conclusions: all reported relationships remain qualitatively the same, although effect sizes show minor variation in some cases.

More broadly, we examined additional analyses that relied on parametric tests (e.g., t-tests) and confirmed that our main conclusions remain unchanged when using non-parametric alternatives. We have updated the manuscript accordingly to ensure that the statistical analyses are appropriate and robust.

Minor points:

10. Fig. S17 e, the x axis tick labels are incorrect

Reply：Thank you for the careful check. We have now corrected this error.

Revised Fig. S17e (page 60):

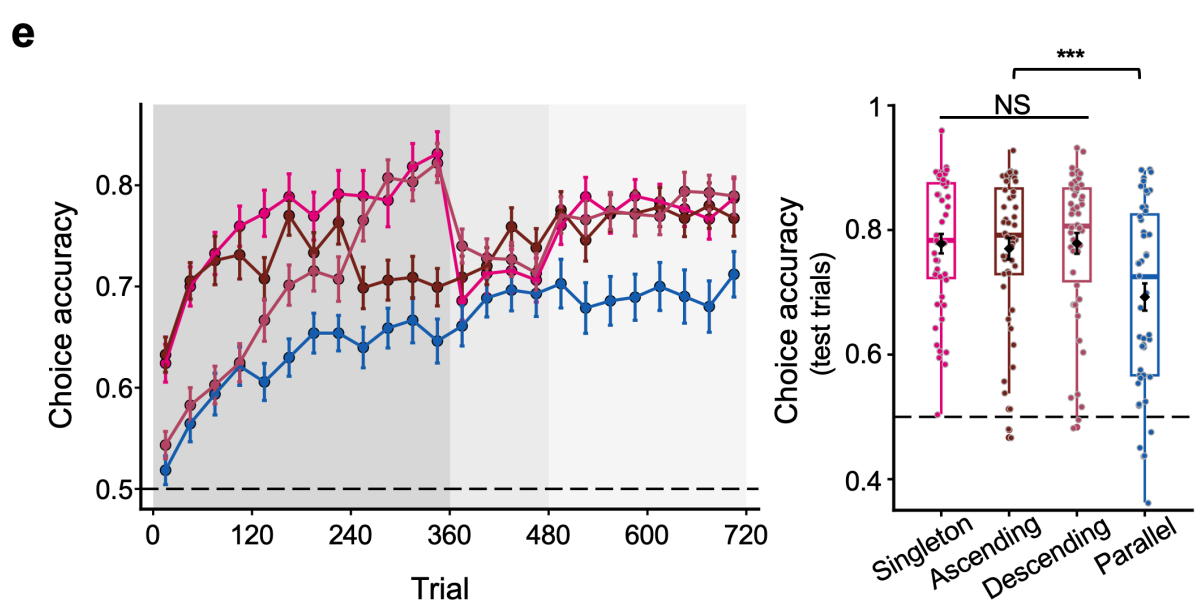

11. Methods p37 for model simulation appear to not have been updated from the previous version (they still contain the previous modeling with 100 epochs).

Reply: Thank you for pointing this out. The description of the 100-epoch simulations was intentionally retained because these simulations serve a specific and limited purpose in the revised manuscript. In particular, we use simulations with pure marginal and pure joint updating to illustrate their asymptotic learning properties—that is, the differences in final performance once learning has converged.

These simulations are not used to fit behavioral data or to draw inferences about learning dynamics. Rather, they provide conceptual insight into why marginal updating is intrinsically less precise: even at convergence, marginal updating learns only the marginal reward probability of each cue and therefore fails to recover the objective WOE values. By contrast, joint updating converges on the true WOEs. This distinction motivates the hybrid framework but is analytically separate from the trial-by-trial model fitting introduced in the revision.

Because the purpose and implementation of these convergence simulations are unchanged from the original version, we retained the corresponding Methods text. To avoid confusion, we have clarified in the revised manuscript that these simulations are illustrative and distinct from the likelihood-based, trial-by-trial modeling used for inference.

Revised Methods (lines 1182-1184):

…These simulations were used to illustrate why marginal updating provides a lower learning precision than joint updating and were distinct from likelihood-based, trial-by-trial modelling used for inference.

Reviewer #3 (Remarks to the Author):

0. The authors have provided a thorough and commendable revision that substantially strengthens the manuscript. Most notably, the transition from snapshot-based simulations to trial-by-trial likelihood-based model fitting improves the modeling framework's ability to capture learning dynamics and supports more rigorous inference about the mechanisms underlying curriculum learning. The inclusion of model recovery and parameter recovery analyses is particularly valuable, as it allows for stronger conclusions about which models, such as hybrid, serial, or optimal accounts, best explain the data and justifies interpretation of parameters in terms of individual or experiment differences. In addition, the authors have systematically formalized and tested a broad range of alternative explanations raised by the reviewers, including differences in learning rates, learning rate decay, and attentional weighting mechanisms. The addition of explicit equations and clearer descriptions of the learning rules substantially improves the clarity with respect to the modeling approach. Overall, these new analyses yield informative insights into how curriculum structure shapes the balance between learning precision and cognitive cost over the course of learning.

   Taken together, the revision includes an impressive array of additional analyses that address all of my concerns from the original review and make this study a strong and laudable contribution to the literature.

   While I do not see any issues that would preclude publication, I have a small number of minor suggestions that the authors may wish to consider if they feel these would further improve the paper.

   Reply: Thank you so much for the positive words and further suggestions for improving the paper. We have carefully considered the remaining minor suggestions and addressed each of them in detail below. We believe these final refinements further improve the clarity and presentation of the manuscript.

1. Predictive vs. generative model performance. It would be helpful to clarify explicitly whether the reported model simulations in Figures R6a and main-text Figures 3e, 4d, 5d, as well as Figures S7b and S7d reflect predictive performance (conditioning on the model on participants' previous choices to predict the next trial) or generative performance (simulating the model from scratch using fitted parameters). I suspect the latter, but I could not find a clear statement in the manuscript. This distinction is important, as generative simulations provide a stronger posterior predictive check, whereas predictive simulations can overstate a model's ability to capture learning dynamics in response to the task environment (see Palminteri et al., 2017, in TiCS).

   Reply: Thank you for this thoughtful point of distinguishing predictive and generative model performance. The recovered choice accuracy curves shown in the mentioned figures reflects predictive performance, in which model predictions are conditioned on participants' previous choices. We have revised the manuscript to make this explicit.

   In addition, we evaluated generative performance by simulating each model from scratch using parameters fitted to individual participants. These simulations showed that the hybrid model could reproduce learning dynamics across conditions (**Fig. R2a**) and that the hybrid model more closely matched the observed learning performance than single-strategy alternatives (**Fig. R2b**). Taken together, evidence from both predictive and generative analyses supports the hybrid model as a better account of participants' behaviour than single strategy models.

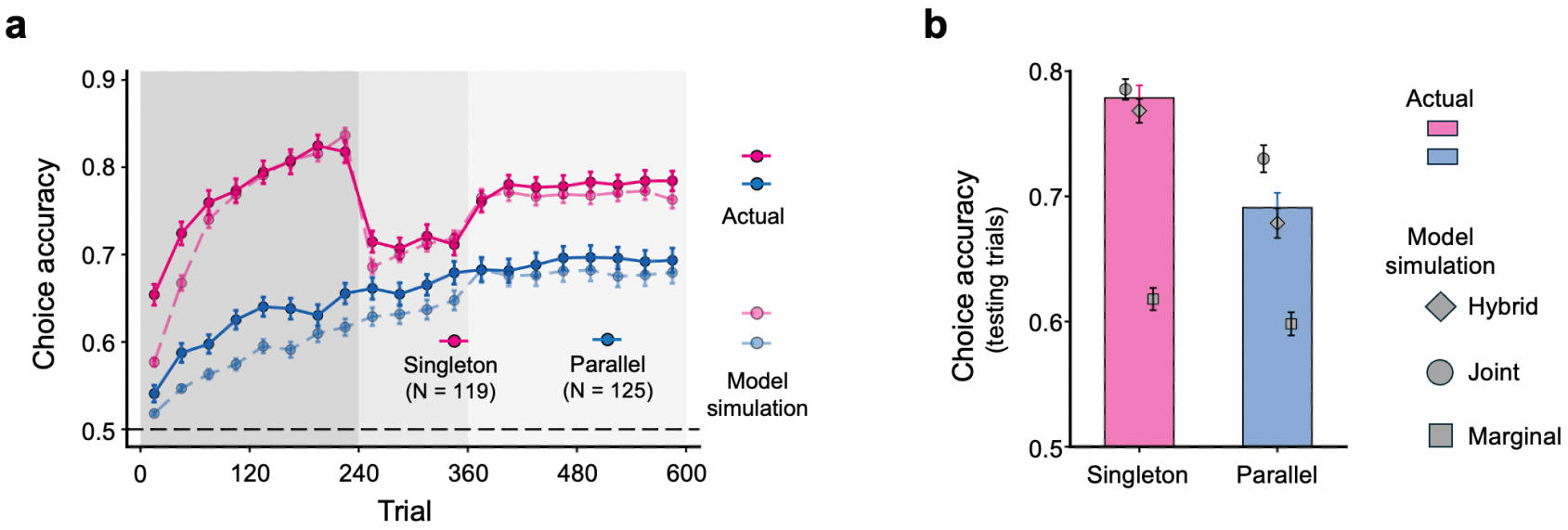

Fig. R2. Generative performance of the models. (**a**) Simulated versus observed choice accuracy across trials. Dark solid lines show pooled accuracy from Exp. 1–3. Light dashed lines show generative performance from the hybrid SD model. (**b**) Model simulated choice accuracy in the testing trials for the three models.

Revised Results (lines 218-220):

> …Light dashed lines show predictive performance from the hybrid SD model and similar patterns were observed when the model predictions were simulated from scratch without using participants' actual choices[37].

2. Scope of model recovery analyses. The model and parameter recovery analyses are a very welcome addition, but I wondered why recovery was demonstrated for only three of the five models listed in Table R1. In particular, it could be informative to explicitly test distinguishability between e.g., the hybrid SD model and the hybrid constant model. Similarly, extending recovery analyses to the alternative models incorporating learning rate decay or attentional weights could further strengthen claims about model distinguishability and ability to rule out corresponding alternative hypotheses. That said, I recognize the practical constraints involved, and this comment is intended as a suggestion rather than a requirement.

Reply: Thank you for this comment. The primary goal of our recovery analyses was related to the central theoretical claim of the paper, that is participants rely on a hybrid learning mechanism rather than a single updating strategy. Accordingly, we focused recovery analyses on the models that speak most directly to this model identification—namely, the hybrid SD model and the two single-strategy models—demonstrating that the hybrid account is both identifiable and distinguishable from purely marginal or purely joint updating. We did not perform recovery analyses for every model variant, because other models differ only in auxiliary features (e.g., fixed vs. adaptive weighting, learning-rate decay, or attentional weights) rather than in their core learning mechanism. Our main conclusions do not hinge on discriminating among these closely related variants, but on establishing that a hybrid strategy outperforms single-strategy alternatives. We have explained this point in the revised manuscript.

Importantly, even without full recovery for all variants, our conclusions are supported by converging evidence: formal model comparison, parameter recovery for the hybrid SD model, robustness checks with alternative mechanisms, and successful out-of-sample predictions. Together, these results provide strong support for the hybrid learning framework and our ability to rule out the main competing explanations.

Revised Legend of Fig. S8 (page 50):

> **Fig. S8. Model and parameter recovery.** (**a**) Model recovery. The model recovery focuses on the hybrid SD model, pure marginal and pure joint updating models without relevant extensions (e.g., learning rate decay and attentional bias), since the central theoretical claim of this study is that participants rely on a hybrid learning mechanism rather than a single

updating strategy. Specifically, we aimed to examine whether the hybrid updating model could be reliably distinguished from the single-strategy alternatives.

3. Interpretation of joint updating in the parallel condition. One result that I found somewhat counterintuitive concerns Figure R7a, where the hybrid SD model appears to infer fewer trials with joint updating in the parallel condition than in the singleton condition, despite participants benefiting more from joint updating in the parallel condition. I may be missing some detail, but a brief explanation of how this pattern should be interpreted would likely help both reviewers and readers.

   Reply: Thank you for pointing out this potential confusion. The key to interpreting the observed pattern lies in the trade-off between learning benefit and cognitive cost in our hybrid framework. While joint updating can improve learning precision when multiple cues are present, it is also more cognitively demanding than marginal updating. As a result, participants are unlikely to adopt joint updating unless it offers a clear advantage over marginal updating.

   In our model, the learning benefit of joint updating depends on how well participants can already distinguish the WOE values of the present cues. This benefit/gain is approximated by the standard deviation of the learned cue weights (WOEs) before a given trial: higher variability indicates that cues are more distinguishable, making joint updating more worthwhile. Early in parallel training, participants have limited knowledge of individual cue values and thus low variability across cues. In this regime, joint updating offers little immediate benefit, and participants tend to rely on the less demanding marginal strategy.

   By contrast, singleton training allows participants to learn cue values more accurately early on, increasing their ability to distinguish cues when they later encounter multi-cue trials. As a result, joint updating becomes more beneficial and is used more often in the singleton condition. Thus, the lower prevalence of joint updating in the parallel condition reflects not its lower potential usefulness, but participants' limited ability to exploit it given their less precise cue representations early in learning.

   Per the reviewer's suggestion, we have now added a brief explanation of why participants have fewer trials with joint updating under parallel condition in the main text, as replicated below.

   Revised Results (lines 301-306):

   > …This proportion increased gradually in both conditions but was consistently lower in the parallel condition (**Fig. 3g**; see **Fig. S11** for example participants). This lower prevalence of joint updating in the parallel condition is expected, as joint updating is more cognitively demanding and is therefore adopted primarily when cues are sufficiently distinguishable—a criterion more readily met after singleton training than during early parallel training.

4. Model comparison criteria. The rebuttal primarily reports AIC- and NLL-based model comparisons. Given the richness of the modeling effort, it might be helpful to briefly justify the choice of AIC over alternatives such as BIC, particularly with respect to model differentiability (e.g., does AIC provide better model differentiability than BIC?; see Wilson & Collins, 2019 in eLife). Even a short note indicating that the main conclusions are robust across criteria would be reassuring.

   Reply: Thank you for this helpful comment regarding (i) the justification of the use of AIC value and (ii) the robustness of our conclusion using other criteria (e.g., BIC). We initially chose AIC for model comparison for two main reasons. First, AIC has been suggested to provide a better model

discriminability, particularly when comparing closely related models (Collins, 2025). Second, BIC imposes a stronger penalty on model complexity that scales with sample size, which can overly penalize richer models, such as our hybrid learning model, given the large number of trials per participant (≥600).

To ensure robustness, we also computed BIC for the three main models for each experiment (see the table below). The results mirrored those obtained with AIC: the hybrid learning model consistently outperformed single-strategy alternatives across experiments, reflected by the lower BIC values, although effect sizes were smaller under BIC. Thus, our central conclusion that a hybrid learning model better accounts for participants' behaviour does not depend on the choice of model comparison criteria.

ΔBIC value (mean ± SEM)
relative to the hybrid SD model

	Marginal	Joint
Exp. 1 (*N* = 109)	33.99 ± 4.11	5.60 ± 1.94
Exp. 2 (*N* = 83)	50.44 ± 5.70	12.06 ± 2.67
Exp. 3 (*N* = 52)	35.30 ± 5.80	4.41 ± 1.67
Exp. 4 (*N* = 86)	22.97 ± 4.04	13.89 ± 2.63
Exp. 5 (*N* = 52)	17.11 ± 4.51	12.36 ± 2.84
Exp. 6 (*N* = 192)	36.68 ± 3.18	5.47 ± 1.21

We have revised the manuscript to incorporate these points accordingly.

Revised Results (lines 282-284):

> …Notably, the hybrid SD model continued to outperform single-strategy alternatives even when model complexity was more strongly penalized using the Bayesian Information Criterion (BIC).

Revised Methods (lines 1230-1233):

> …We also compared models using the Bayesian Information Criterion (BIC), which more strongly penalizes model complexity. Specifically, for each model,
> $$BIC = -2 \times LL + K \times \ln(n)$$
> where n is the total trial number for each participant.

- Collins, A. G. E. (2025). A habit and working memory model as an alternative account of human reward-based learning. *Nature Human Behaviour*. https://doi.org/10.1038/s41562-025-02340-0

5. Reporting fitted parameter distributions. It would be useful to report the distributions of fitted parameters for the final selected model, perhaps in the supplementary materials. This would allow

readers to verify that parameters such as α⁺ and α⁻ lie well within their predefined bounds, such as the [0, 1] interval, and would further rule out potential fitting pathologies that are not uncommon in models of this kind.

Reply: Thank you for this great suggestion. We have now replaced the original bar plots (mean±SEM) with the violin plots to additionally show the distribution of the fitted parameters in the hybrid learning model across all participants (**Figs. S9 a-d**).

The fitted parameters of α_+, β_1 and θ lie well within the predefined bound. However, we notice that the fitted α_- for most participants is close to 0 although the predefined bound for α_- is [0, 1]. This observation is consistent with previous studies (Farashahi et al., 2019; Palminteri & Lebreton, 2022) and suggests a relatively small influence of a negative outcome on learning, possibly related to the positivity bias.

Revised Figs. S9 a-d (page 52):

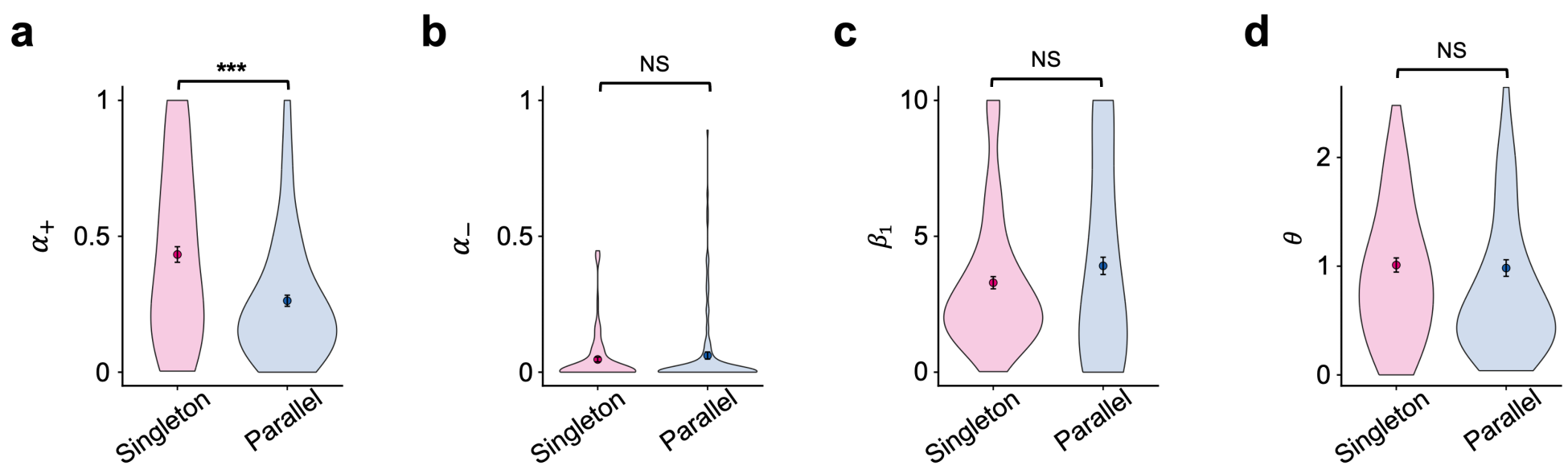

Fig. S9. Estimated parameters of hybrid SD model. (**a–d**) Violin plots show the distributions of the four free parameters across participants, plotted separately for the singleton and parallel conditions. Dots and error bars indicate the mean and SEM within each condition.

- Farashahi, S., Donahue, C. H., Hayden, B. Y., Lee, D., & Soltani, A. (2019). Flexible combination of reward information across primates. *Nature Human Behaviour*, 3(11), 1215–1224. https://doi.org/10.1038/s41562-019-0714-3
- Palminteri, S., & Lebreton, M. I. (2022). The computational roots of positivity and confirmation biases in reinforcement learning. *Trends in Cognitive Sciences*, 26(7), 607–621. https://doi.org/10.1016/j.tics.2022.04.005

6. Across-experiment comparisons. Finally, since models were fit across multiple experiments (assuming data was pooled for model comparison), I was curious whether different models might better explain data from different experiments (e.g., whether Experiment 1 favors a different winning model than Experiment 2). This comment is offered primarily out of my own interest, and I do not expect additional analyses unless such results are already available.

Reply: Thank you for this question. We found that hybrid model performed better in fitting participants' choices than single strategy models across all experiments in the current study, including Exp. 1-3 (see **Fig. R3** below).

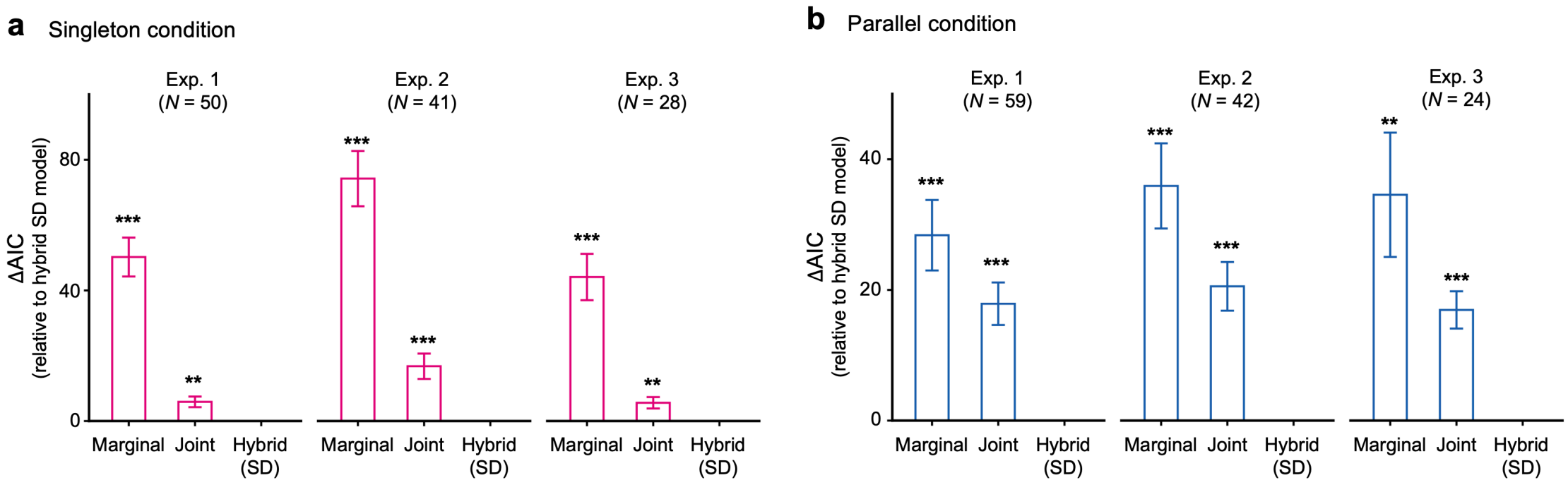

Fig. R3. Mean ΔAIC values are shown for each model and condition, plotted separately for each experiment.

Accordingly, we have now added one sentence in the main text to indicate the robustness of model fitting across Exp. 1-3.

Revised Results (lines 215-216):

> …Notably, the hybrid model consistently yields lower AIC values than single-strategy models across Exp. 1-3.

Editorial remarks:

For the paper to move forward, it is critical that you address the remaining concerns by the reviewers in full (especially Reviewer #1). In particular, we ask you to be transparent about the difference between the high-level theory and the specific model used. Please clarify lines 347-351 on what exactly "Out-of-sample model simulations using the hybrid SD model and parameters derived from Exp. 1–3 predicted" refers to.

Reply: Thank you for this important clarification. We agree that it is essential to clearly distinguish between the high-level theoretical framework and the specific computational model used to evaluate it. In response, we have revised the manuscript throughout, particularly in the Abstract, Results, and Discussion, to make this distinction explicit and consistent.

Regarding former lines 347-351, we have rewritten the passage to clarify what we mean by “out-of-sample model simulations.” In brief: the predictions being tested come from the central claim of our theoretical framework–namely, that learners rely on both marginal and joint updating under cognitive constraints. The hybrid SD model is then used to formalize this idea and quantify its consequences by simulating behavior under new curricula using parameters independently estimated from Exp. 1-3. We refer to these simulations as “out-of-sample” because both the model and the parameter values were estimated from different participants and training conditions rather than those used in the follow-up experiments. We now make clear that these simulations served to test whether the theoretical account was consistent with behavior in new settings, rather than to independently generate novel theoretical predictions.

We greatly appreciate the editor’s guidance on this issue and believe the revised manuscript now presents a transparent and accurate account of the relationship between the theoretical framework and the specific model used.

Reviewer #1 (Remarks to the Author):

I thank the authors for being responsive to the reviews, and most points have been well addressed, the study is well done and results are interesting.

I am still not fully comfortable with the general framing. My previous point 6 on claims around modeling is still not fully addressed. Despite the two edits in the result, mutliple places in the paper still overstate the claims of prediction by the *cognitive model* as opposed to the broader theory.

For example, the abstract still over claims predictiveness of the model; however, it is not the cognitive model, as stated, that was used to accelerate human learning. The authors use “framework” in their answer to my previous point 6, and adequately revised the results section, but not the abstract.

Same, the paragraph starting l 337 is still misleading. e.g . L347-351 implies simulations from the presented cognitive model generated the predictions, which is not the case. Again, there should be no confusion between the high level theory (which made accurate predictions) and the quantitative cognitive model presented in the paper (which was not predictive, and is instead postdictive). Same l 397, l433, l449 etc. I can’t list them all, but this should be adjusted throughout.

I agree with the authors that in the end what matters is that the theory predicts the results; but the results as framed overstate the methodological use of the quantitative model simulations for generating predictions, which is an unsupported claim in this paper. I do not see this as a minor language point, but an important point that overstates the contributions of the paper.

Reply: Thank you for the positive words on our revisions. We greatly appreciate the reviewer for the careful follow-up on this point. We agree that it is crucial to distinguish clearly between predictions derived from the broader theoretical framework and results supported by the quantitative model.

To address this, we went back through the Abstract, Results, and Discussion and revised the framing throughout. The text now makes clear that the predictions tested in the follow-up experiments come from the central claim of the theoretical framework: learners do not

rely exclusively on joint updating, but also adopt a simpler marginal updating strategy under cognitive constraints. The computational model is presented as a way to formalize this hypothesis and quantify its implications, rather than as the source of prospective predictions.

Concretely, we have revised the Abstract and the passages noted by the reviewer (including the sections around former lines 337, 347-351, 397, 433, and 449), as well as other related text, to remove any implication that simulations from the fitted model independently generated the predictions. The revised manuscript now consistently states that the theoretical account motivated the experimental tests, and the model was used to evaluate and interpret the resulting behavior.

Reviewer #3 (Remarks to the Author):

I commend the authors for their thorough second revision. They have addressed all of my previous concerns, and I have no substantive issues remaining. The only minor point is that the statement "joint updating is more cognitively demanding" might be phrased more cautiously. Given the lack of definitive evidence, it would be preferable to frame this as a hypothesis, e.g., "joint updating is assumed to be more cognitively demanding", rather than as an established fact. With this final clarification, I believe the manuscript makes a valuable contribution and is ready for publication.

Reply: Thank you for this helpful suggestion. We agree that the wording was too strong given the available evidence. We have updated the manuscript to frame this as an assumption, replacing “joint updating is more cognitively demanding” with “joint updating is assumed to be more cognitively demanding.” This keeps the intended logic of the framework while being more appropriately cautious.
